# Mapping DEHP-induced metabolic dysregulation through multi-omics and systems biology: an adverse outcome pathway network approach

**DOI:** 10.3389/ftox.2026.1930835

**Published:** 2026-09-30

**Authors:** Elisavet Renieri, Achilleas Karakoltzidis, Nafsika Papaioannou, Dayna R. Schultz, Thanasis Papageorgiou, Catherine Gabriel, Ilias S. Frydas, Kévin Bernal, Xavier Coumoul, Etienne B. Blanc, Normand Podechard, Hélène Le Mentec, Charbel Touma, Sophie Langouët, Talía Boronat-Belda, Hilda Ferrero, Paloma Alonso-Magdalena, Karine Audouze, Spyros Karakitsios, Dimosthenis A. Sarigiannis

**Affiliations:** 1 Environmental Engineering Laboratory, Department of Chemical Engineering, Aristotle University of Thessaloniki, Thessaloniki, Greece; 2 HERACLES Research Center on the Exposome and Health, Center for Interdisciplinary Research and Innovation, Balkan Center, Thessaloniki, Greece; 3 National Hellenic Research Foundation, Athens, Greece; 4 Université Paris Cité, Inserm, Health and Functional Exposomics (HealthFex), UMR‐S 1124, Paris, France; 5 University Rennes, Inserm, EHESP, Irset (Institut de recherche en santé, environnement et travail) - UMR_S 1085, Rennes, France; 6 Instituto de Investigación, Desarrollo e Innovación en Biotecnología Sanitaria de Elche (IDiBE), Universidad Miguel Hernández de Elche, Elche, Spain; 7 Departamento de Fisiología, Genética y Microbiología, Universidad de Alicante, Alicante, Spain; 8 CIBER de Diabetes y Enfermedades Metabólicas Asociadas (CIBERDEM), Instituto de Salud Carlos III, Madrid, Spain; 9 Science, Technology and Society Department, Environmental Health Engineering, School for Advanced Study (IUSS), Pavia, Italy; 10 School of Medicine, Brady Urological Institute, Johns Hopkins University, Baltimore, MD, United States; 11 Yale School of Public Health, Yale University, New Haven, CT, United States

**Keywords:** adverse outcome pathway network (AOPN), DEHP (di(2-ethylhexyl) phthalate), metabolic disruption, multi-omics, systems toxicology

## Abstract

Di(2-ethylhexyl) phthalate (DEHP) is a widespread endocrine-disrupting plasticizer associated with metabolic disorders. To elucidate their mechanistic basis, we developed an Adverse Outcome Pathway Network (AOPN) informed by DEHP-induced perturbations, by integrating multi-omics data, systems biology modeling, and targeted literature evidence. Multi-omics data were generated in 3D human hepatic cells, pancreatic β-cells, and zebrafish larvae exposed to DEHP across biologically relevant concentration ranges. Transcriptomic and metabolomic analyses revealed perturbations in lipid metabolism, mitochondrial function and oxidative stress pathways, including altered sphingolipid signaling and dysregulation of sphingosine-1-phosphate (S1P). Systems biology modeling contextualized these perturbations within metabolic pathways and generated dose-dependent predictions for key intermediates, highlighting potential reinforcing feedback mechanisms such as ATP–ROS interactions that may further destabilize metabolic homeostasis. Integrating experimental and literature evidence identified key event relationships proposed to link early molecular perturbations to downstream adverse metabolic outcomes, including hepatic steatosis, insulin resistance, non-alcoholic fatty liver disease (NAFLD), and obesity. Central mechanistic features of the network converge on lipid remodeling, mitochondrial dysfunction, oxidative stress, serine/S1P pathway disruption, and impaired insulin signaling. Because the individual omics contrasts are based on three replicates per condition, statistical support in this work is evaluated at pathway rather than single-feature level: under Benjamini-Hochberg control almost no individual transcript is significant, whereas the pathways carrying the mitochondrial and lipid-handling key events remain significant in competitive gene-set tests. Cross-system comparison showed that suppression of oxidative-energy metabolism is the only module conserved across all three systems, whereas PPAR signalling and fatty-acid handling are liver-specific and non-monotonic. Together, these data support a coherent, evidence-integrating mechanistic hypothesis linking early molecular events to adverse metabolic outcomes across multiple levels of biological organization, rather than a quantitatively validated causal pathway. To our knowledge, this study provides a novel integrative AOPN framework combining multi-omics data across liver and pancreatic models and reveals previously underappreciated metabolic cross-talk, including involvement of serine and S1P pathways. The proposed framework is explicitly hypothesis-generating and evidence-integrating: it provides a transparent, FDR-audited basis for future quantitative AOP development and supports mechanistically informed chemical risk assessment by identifying early key events, potential biomarkers, and intervention points relevant to human metabolic health.

## 1 Introduction

Di(2-ethylhexyl) phthalate (DEHP) is extensively used as a plasticizer in numerous consumer products, leading to pervasive human exposure (Net et al., 2015; Wang et al., 2019; Mansuri et al., 2025). Despite regulatory restrictions in several applications, DEHP continues to be detected in environmental matrices and human biomonitoring studies (Jurewicz and Hanke, 2011; Katsikantami et al., 2016; Wang et al., 2019) and is widely used as a prototypical compound to investigate mechanisms of phthalate-induced metabolic disruption. Recognized for its endocrine-disrupting properties (Ri et al., 2024; Moghazy et al., 2025), DEHP has been associated with metabolic disorders such as obesity (Radke et al., 2019; Heindel et al., 2022; Perez-Diaz et al., 2023) and non-alcoholic fatty liver disease (NAFLD; recently re-termed metabolic dysfunction-associated steatotic liver disease, MASLD). The NAFLD terminology is retained throughout this work to ensure consistency with the existing AOP framework and the legacy literature (Chen et al., 2022; Chen et al., 2023; Rinella et al., 2023; Sun et al., 2024). While DEHP’s effects on metabolic pathways have been investigated, a unified mechanistic picture, connecting molecular changes to adverse metabolic outcomes, has been lacking. Understanding the mechanistic pathways through which DEHP exerts its toxic effects is essential for accurate risk assessment and regulatory decision-making (Zare Jeddi et al., 2021).

DEHP contributes to metabolic disruption particularly by altering lipid metabolism, through nuclear receptor pathways (e.g., LXR/SREBP and PPARα/γ), while also inducing oxidative stress via AhR-dependent reactive oxygen species generation and Nrf2 activation, promoting inflammatory responses, and disrupting insulin signaling through the PI3K/Akt pathway across multiple experimental models (Rusyn et al., 2006; Rengarajan et al., 2007; Pan et al., 2022; Chen et al., 2023; Zhang et al., 2023; Lee et al., 2024).

Given the complexity of these interactions, a systems-level approach is required to capture the cascade of molecular events leading to adverse health outcomes (Sturla et al., 2014; Sarigiannis and Salifoglou, 2016; Thomas et al., 2017). Systems biology approaches can combine high-throughput multi-omics (e.g., transcriptomics and metabolomics) with computational modeling to reconstruct biological networks and quantify perturbations (Bhattacharya et al., 2011; Canzler et al., 2020; Valls-Margarit et al., 2023). These data-driven models support a mechanistic understanding of chemical toxicity and its links to human health outcomes (Bhattacharya et al., 2012; Sturla et al., 2014). Multi-omics methodologies, however, while powerful in revealing disrupted nodes within biological pathways, may overlook subtle or statistically insignificant changes, and present challenges in interpretating functional relevance due to the sheer volume of data and temporal complexity (Braeuning et al., 2018; Song et al., 2018; Tarazona et al., 2021). Nevertheless, such low-level perturbations can cumulatively drive adverse health outcomes. By integrating diverse omics layers with computational modeling, systems biology helps to bridge this gap, capturing latent or sub-threshold effects within the broader network context and improving confidence in pathway response detection (Zhang et al., 2018; Canzler et al., 2020; Perkins et al., 2022).

The Adverse Outcome Pathway (AOP) framework, when coupled with systems biology, provides a structured means to integrate mechanistic knowledge across biological levels (from molecules to organs) (Villeneuve et al., 2014a; Perkins et al., 2022; Saarimäki et al., 2023). Traditionally linear, the AOP framework can be expanded through systems modeling to incorporate feedback and feedforward loops, making it more reflective of real-world biological complexity and dynamic interactions (Conolly et al., 2017; Cronin and Richarz, 2017; Leist et al., 2017; Ankley and Edwards, 2018; Aguayo-Orozco et al., 2019). Within this context, although AOPs are conceptually stressor-agnostic, prototypical stressors are commonly used to anchor and inform mechanistic linkages within a network (Leist et al., 2017; Ankley and Edwards, 2018). Prior AOP frameworks and network case studies, such as the AOP network for metabolic disorders involving steatosis (Knapen et al., 2018) and recent efforts mapping DEHP to AOP networks for other endpoints such as female reproductive toxicity (Pogrmic-Majkic et al., 2022), demonstrate the utility of linking pathways. However, a comprehensive AOP network using DEHP-relevant evidence has not yet been established. Here, we address this gap by leveraging DEHP as an evidence-rich prototypical stressor within a multi-omics and systems biology framework to construct an Adverse Outcome Pathway Network that elucidates DEHP’s metabolic toxicity.

In this work, we applied an integrated methodology combining multi-omics data and systems biology to elucidate DEHP-induced toxicity mechanisms. DEHP served as a case study within the OBERON project (https://oberon-4eu.com/), using data from *in vitro* (HepaRG liver cells cultivated in 3D, EndoC-βH1 pancreatic β-cells), *in vivo* (zebrafish), and the literature. By linking exposure, internal concentrations, and molecular responses across biological scales, we constructed an adverse outcome pathway network capturing the interconnected molecular events and systemic effects of long-term DEHP exposure. This approach enhances our mechanistic understanding of DEHP-induced metabolic disruption using an integrated, systems-level framework.

## 2 Methods

For the identification of KEs and AOs, a multi-step approach was employed. First, transcriptomic and metabolomic data were integrated into a multi-omics analysis in order to identify molecular signatures of DEHP and corresponding perturbed pathways. These analyses were performed across various models and organisms, including pancreatic (EndoC-βH1 pancreatic β-cells) and liver (3D HepaRG culture) cells, as well as zebrafish (*Danio rerio)* larvae, to gain a holistic view of effects across multiple lines of evidence. The three systems are complementary rather than redundant, and each was selected for a capability the others lack. The human 3D HepaRG spheroid provides a human-relevant hepatic system with retained xenobiotic metabolism, which is essential because DEHP acts largely through its metabolite MEHP; the human EndoC-βH1 line provides the pancreatic β-cell compartment, which is entirely absent from a hepatic model and cannot be resolved in whole-larva zebrafish homogenates; and zebrafish larvae provide whole-organism integration with intact inter-organ signalling and an *in vivo* internal-dose context. A zebrafish-only design would have been unable to attribute a response to the hepatocyte or the β-cell, since the omics were performed on whole-larva homogenates, and would have used a non-human receptor and metabolic complement. Conversely, an in vitro-only design could not test whether the responses persist when tissues can compensate for one another. The cross-system comparison this enables is reported in Section 3.4 and Figure 9. Next, a dynamic systems biology model was employed to simulate the interactions of molecular entities within pathways (Ashworth et al., 2016). Dose-response modeling, using experimental data from HepaRG *in vitro* experiments described in this work, was used to parameterize model inputs and support mapping of key events and their progression towards adverse outcomes. Additional evidence generated within the OBERON project using HepaRG hepatic models, EndoC-βH1 pancreatic β-cells, and zebrafish was incorporated to support the evaluation of the identified KEs and AOs. Finally, a comprehensive literature review was conducted to identify molecular targets and pathways affected by DEHP to further bolster findings from experimental and computational analyses. Detailed experimental protocols and analytical parameters are provided in Supplementary Material S2 (SM2).

### 2.1 Cell cultures and zebrafish husbandry

EndoC-βH1 pancreatic β-cells were cultured under standard conditions (37 °C, 5% CO_2_) in complete culture medium as previously described. For chemical treatment, cells were exposed to 1, 10, and 100 nM DEHP for 7 days in phenol red–free medium supplemented with charcoal dextran-treated FBS. These concentrations were chosen based on prior OBERON project findings showing that these low DEHP doses reduce insulin secretion and insulin content in pancreatic β-cells (Al-Abdulla et al., 2022). Further details on EndoC-βH1 maintenance and exposure conditions are provided in Supplementary Material S2 (SM2).

For human liver 3D cultures, the Hepoid® model was used to generate differentiated HepaRG spheroids, as previously described (Rose et al., 2021; 2022). HepaRG spheroids were exposed to DEHP at 100 pM, 100 nM, and 10 μM (vehicle: 0.1% DMSO) for 14 days, beginning on the fifth day of culture. Detailed culture conditions and quality control steps are provided in Supplementary Material S2 (SM2).

In zebrafish larvae, DEHP exposure was performed from 3 to 5 days post-fertilization (dpf) at non-toxic concentrations. DEHP was dissolved in DMSO (final concentration <0.03%) or water. Additional husbandry and exposure details are reported (Le Mentec et al., 2023). A concise summary of exposure concentrations (0.025 and 10 μΜ) and experimental design is provided in SM2. All zebrafish transcriptomic and metabolomic data reported in this work derive from embryo-larval stages, with exposure from 3 to 5 dpf and sampling at 5 dpf; no adult fish were used. Under Directive 2010/63/EU, zebrafish are not protected until the onset of independent feeding (approximately 5 dpf), so this design additionally minimises regulated animal use, consistent with the ethics statement.

Exposure concentrations were not fully harmonised across the three experimental systems, and this was a deliberate consequence of the OBERON design rather than an oversight. Each system was anchored to concentrations previously shown to be sub-cytotoxic and functionally active in that specific model: 1–100 nM in EndoC-βH1 cells, where these low doses reduce insulin secretion and insulin content; 100 pM–10 µM in Hepoid® HepaRG spheroids, spanning human-relevant internal concentrations up to a positive-control level for lipid accumulation; and 0.025 and 10 µM in zebrafish larvae, bracketing the maximum non-teratogenic concentration in that model. The ranges overlap in the 100 nM–10 µM window, which is where cross-system comparison is made (Figure 9). Full harmonisation would have required exposing at least one model outside its viable or mechanistically informative range, which would have confounded the comparison it was intended to enable.

### 2.2 Transcriptomics–Metabolomics

Transcriptomic, metabolomic, and integrated multi-omics profiling was performed on samples obtained from EndoC-βH1 pancreatic β-cells and 3D human liver Hepoid® cultures and 5-dpf zebrafish larvae.

#### 2.2.1 Transcriptomics

With regard to *in vitro* experiments, cell line samples and RNA extracts were received from partner institutes. Transcriptomic profiling was performed using Agilent one-colour microarrays, as described in detail in Supplementary Material S2 (SM 2).

Briefly, RNA samples were labelled and hybridized according to the manufacturer’s one-colour microarray protocol (Agilent Technologies). Quality control criteria for labelling efficiency, yield, and hybridization performance were met for all samples. Full experimental protocols, reagents, and quality control metrics are reported in SM2.

##### 2.2.1.1 Bioinformatics analysis of transcriptomics data using R/limma

Results from *in vitro* and *in vivo* systems were processed using the same methods. Scan results were subjected to quality control with Feature Extraction Software (Agilent v.11.5.1.1). Raw data were exported as.txt files and imported into R (R Core Team, 2016). Data preprocessing and differential expression analysis were conducted using the limma package, following workflows optimized for one-colour Agilent microarrays.

Raw intensities were identified and background corrected (“normexp”, offset = 50) and normalized using “quantile” normalization. Replicate probes mapping to the same gene were averaged. Batch effects were assessed using principal component analysis and hierarchical clustering, and array quality weights were applied where appropriate to account for residual variability.

Statistical analysis was conducted using the limma moderated t-test, and multiple-testing correction was performed using the Benjamini-Hochberg method. Genes were considered differentially expressed based on a log_2_ fold-change threshold (|log_2_FC| ≥ 0.58 for cell lines; ≥ 1.3 for zebrafish) and/or statistical significance (adjusted *p* ≤ 0.05), as described below under Statistical and Biological Relevance Criteria. Annotated gene identifiers were mapped to Entrez IDs using org.Hs.eg.db.

##### 2.2.1.2 Bioinformatics analysis of transcriptomics data using Agilent GeneSpring™

In parallel to the R/Bioconductor-based workflow, transcriptomics data from *in vitro* and *in vivo* systems were also processed using Agilent GeneSpring™ (v14.9) to support quality control, batch correction, and confirmation of differential expression results. Raw intensity files generated with Feature Extraction Software (Agilent) were imported into GeneSpring™, normalized, and batch-corrected using standard workflows, including ComBat-based correction and quantile normalization.

Quality control and outlier assessment were performed using principal component analysis, sample correlation analysis, and probe detection flags. Differential expression analysis was carried out using moderated statistical testing with multiple testing correction. Fold-change and adjusted *p*-value thresholds were harmonized with those applied in the R/limma-based analysis, ensuring consistency in feature selection across analytical environments. Detailed preprocessing parameters and quality control metrics are reported in SM2.

#### 2.2.2 Metabolomics

##### 2.2.2.1 Sample preparation

Metabolomic profiling was performed on *in vitro* and *in vivo* samples using a UHPLC–HRMS (QTOF) platform. Polar and non-polar metabolites were extracted using matrix-adapted workflows, and pooled quality control (QC) samples were included to monitor analytical performance and enable correction of systematic variability. Detailed extraction procedures, solvents, internal standards, and sample preparation workflows are provided in Supplementary Material S2 (SM2).

##### 2.2.2.2 Chromatographic separation and mass spectrometry

Chromatographic separation was performed using reversed-phase and HILIC methods, and metabolite detection was carried out in both positive and negative ionisation modes under full-scan acquisition conditions. Instrument configurations, chromatographic gradients, and acquisition parameters are detailed in SM2.

##### 2.2.2.3 *Data preprocessing* and metabolite identification

Raw data were converted to mzML format and processed in R using established metabolomics workflows (XCMS and CAMERA) for peak detection, retention-time alignment, feature grouping, and annotation of isotopes and adducts. Data quality was assessed using QC-based diagnostics and batch-effect evaluation. Detailed preprocessing parameters are provided in SM2. Low-quality features and features with excessive missing values were removed prior to statistical analysis. Data were log-transformed, normalized, and batch-corrected using QC-based approaches to minimize analytical variability. Putative metabolite annotation was performed using xMSAnnotator with reference to HMDB, KEGG, and LipidMaps. Selected metabolites were confirmed using authentic standards or MS/MS spectral matching where available. Full identification workflows and confidence criteria are reported in SM2.

##### 2.2.2.4 Statistical analysis

The significantly differential metabolites were determined by using the ANOVA unequal variance test in R followed by the Benjamini-Hochberg false discovery rate (FDR) correction for multiple comparisons to minimize false positives. An adjusted *p*-value cut-off of 0.05 was considered. The fold-change was calculated as the ratio between exposed and control samples. The resulting metabolites were filtered considering a fold-change cut-off of 2.0.

#### 2.2.3 Statistical and Biological Relevance Criteria

For both transcriptomics and metabolomics analyses, features were filtered for biological relevance using a dual-criterion approach. Specifically, features were retained if they met either of the following conditions: (i) a strong magnitude of response combined with statistical significance (adjusted p ≤ 0.05), or (ii) a strong magnitude of response alone, even if statistical significance was not achieved. Magnitude thresholds were defined as |log_2_FC| ≥ 0.58 for *in vitro* cell models and |log_2_FC| ≥ 1.3 for zebrafish.

This approach was adopted to capture potentially meaningful biological perturbations that may not reach formal statistical significance at n = 3 replicates per condition, a common constraint in multi-model omics studies. We emphasise that criterion (ii) is a hypothesis-generating filter and not a claim of statistical significance; features admitted on magnitude alone are candidate perturbations whose role in the network rests on convergent evidence from the second omics layer, the other experimental systems, and the literature, rather than on the individual measurement. All transcriptomic and metabolomic features discussed in the Results meet at least the magnitude-based relevance threshold, ensuring consistency and transparency in feature selection.

#### 2.2.4 Sensitivity analysis under strict FDR control

Sensitivity analysis under strict FDR control. To quantify how far the network depends on this relaxed criterion, we repeated the entire differential-expression analysis under Benjamini-Hochberg control and report the outcome transparently. Raw per-sample expression matrices for all three models (deposited at https://doi.org/10.5281/zenodo.20376941) were refitted in *limma* with robust empirical-Bayes moderation and intensity trend, with experimental replicate included as a blocking factor, and BH-adjusted p-values were computed within each contrast. The full refitted statistics (per-gene logFC, p and BH-FDR for all contrasts), the CAMERA gene-set results and the attrition counts are provided in Supplementary Material S3.

Two methodological points are recorded here so that the reanalysis in Supplementary Material S3 can be reproduced exactly. First, control and treated arms were processed as matched experimental replicates, and Supplementary Material S2 identifies experimental replicate as the dominant component of variance in these datasets; replicate was therefore included as a blocking factor. Omitting it leaves replicate-level offsets in the residual variance and, in the zebrafish data in particular, yields large numbers of nominally FDR-significant transcripts at negligible effect sizes. Second, the gene-set definitions used for the competitive tests reported in Supplementary Material S3 were taken from the MSigDB distribution of KEGG rather than retrieved from KEGG directly; the two differ slightly in pathway membership and naming, so individual adjusted p-values differ in detail while the pattern of which pathways are significant, in which systems and in which direction, is unchanged.

### 2.3 Multi-omics

For the integration of annotated genes and metabolites, a structured workflow was employed in which transcriptomics and metabolomics data were collected from DEHP-exposed samples at concentrations of 25 nM and 10 μM in 5 dpf zebrafish larvae, 100 pM, 100 nM, and 10 μM in Hepoid® HepaRG cultures, and 1, 10 and 100 nM in EndoC-βH1 pancreatic β-cells. Exact exposure conditions and experimental designs for each model are detailed in Supplementary Material S2 (SM2). Data were normalized and log-transformed prior to multi-omics analyses to reduce heteroscedasticity and scale differences, minimize batch effects, and ensure comparability across omics layers.

The DIABLO method, included in the mixOmics R package (Singh et al., 2019), was used to integrate annotated genes and metabolites in a supervised manner, with the aim of identifying correlated multi-omics features associated with DEHP exposure. Joint pathway analysis was subsequently performed using MetaboAnalystR v4.0 (Pang et al., 2024) incorporating both biologically relevant features identified by DIABLO, and statistically significant differentially expressed genes (DEGs) and differentially expressed metabolites (DEMs) using a *p*-value threshold of < 0.05, along with fold change cut-offs of > 1.5 for genes and > 2 for metabolites.

Entrez ID and HMDB ID were selected as identifiers for the imported genes and metabolites. After data import, the genes and metabolites were mapped to KEGG metabolic pathways for enrichment analysis using Fisher’s Exact Test and for pathway topology analysis, with betweenness centrality as the topology measure. The results from the joint pathway analysis were summarized in scatter plots and in summary tables containing statistics for all pathways, including the number and names of matched genes and metabolites, *p*-values, and impact factors.

### 2.4 Incorporation of *in vitro* and *in vivo* data generated within the OBERON project

To support the mechanistic interpretation derived from the multi-omics and systems biology analyses, additional *in vitro* and *in vivo* evidence generated within the OBERON project was incorporated. These experimental datasets targeted key metabolic endpoints relevant to DEHP-induced metabolic disruption across complementary biological systems.

In Hepoid® HepaRG 3D hepatic cellular models, the effects of DEHP on lipid metabolism were assessed by quantifying total triglycerides concentration and lipid droplet accumulation (Bernal et al., 2024). For the KER weight-of-evidence (WoE) evaluation (SM1), complementary evidence from HepaRG 2D hepatic cellular models was also considered, including lipid accumulation, fatty acid oxidation and expression of lipid metabolism–related genes (Bernal et al., 2024). Carbohydrate metabolism was evaluated through measurements of glucose export, glucose consumption, and glycogen content, while cellular energy metabolism was assessed using Seahorse technology (Bernal et al., 2026).

In pancreatic cellular models, β-cell functional responses to DEHP exposure were assessed, including insulin secretion, content, electrical activity, viability and the expression of genes related to β-cell function and identity, as reported elsewhere (Al-Abdulla et al., 2022).

Finally, *in vivo* models were used to evaluate systemic and steatogenic effects of DEHP exposure, as described in detail in Le Mentec et al. (Le Mentec et al., 2023).

### 2.5 Systems biology modelling

The systems biology model applied here is the deterministic, dynamic ordinary differential equation (ODE) model of hepatic carbohydrate and lipid metabolism developed by (Ashworth et al., 2016) was employed to contextualise the experimentally observed lipid perturbation and examine its predicted downstream metabolic network effects of DEHP exposure. It is a kinetic, mass-balance model rather than a statistical or graph-theoretic one: state variables are metabolite concentrations, and each reaction is represented by an explicit rate law (Michaelis-Menten or mass-action) with literature-derived kinetic parameters. Numerical integration therefore produces continuous concentration-time trajectories for every metabolite in every compartment, and DEHP is represented not as a node in a network but as a multiplicative modifier of specific rate constants.

The core structural innovation of this model is its treatment of the porto-central sinusoid axis, rather than the individual hepatocyte, as the fundamental repeating unit of the liver. The sinusoid was discretized into eight serial compartments to explicitly represent the metabolic zonation from the periportal (PP) to the pericentral (PC) region. Each compartment contained a detailed representation of key metabolic pathways, including glycolysis, gluconeogenesis, glycogen synthesis/degradation, fatty acid β-oxidation, *de novo* lipogenesis, triglyceride synthesis, and oxidative phosphorylation. Crucially, the expression and activity of enzymes governing these processes were zonated based on experimental literature, with specific periportal-to-pericentral ratios applied to rate constants (e.g., higher β-oxidation in the PP region, higher lipogenesis in the PC). The model also incorporated hormonal regulation by insulin and glucagon, secreted by pancreatic β- and α-cells, respectively, with their concentrations dynamically affecting hepatocytes along the sinusoid. Substrate inputs (glucose, free fatty acids) were provided to a separate “body” compartment in a spiked pattern to simulate postprandial and interprandial states.

Computationally, the model comprises 8 sinusoidal compartments plus a systemic “body” compartment, giving a coupled system of ordinary differential equations of the form dC/dt = S·v(C,p), where C is the vector of metabolite concentrations, S the stoichiometric matrix, v the vector of reaction rates and p the parameter vector. The system was integrated numerically with a stiff solver (LSODA) over a 24 h feeding-fasting cycle until a stable limit cycle was reached; reported values are taken from the final cycle. This is a genuine dynamic simulation, not a static network-connectivity analysis. The system is of modest size by ODE standards (tens of state variables per compartment) and integrates in seconds, so computational cost is not limiting.

Metabolic “flux” in this work therefore refers to model-computed reaction rates v(C,p), reported in µM h^-1^, and not to isotopically measured flux. DEHP effects were introduced by scaling the *de novo* lipogenesis rate constant, with the scaling factor for each concentration obtained by fitting the measured palmitate dose-response (Figure 3) to the model’s lipogenic output. Acetyl-CoA and ATP trajectories (Figure 4) are then predictions of the model given that single perturbation, not independent fits. The measured palmitate dose–response was the only experimental dataset used to modify a model parameter directly. Other transcriptomic and metabolomic findings were used for mechanistic contextualisation and comparison with the predicted pathway responses but were not individually mapped onto model parameters (Karakoltzidis, 2026). Because no ^13^C tracer or Seahorse flux data were used to constrain these rate constants in the present study, the simulated fluxes should be read as mechanistic hypotheses consistent with the measured metabolite levels, not as measured flux values.

### 2.6 Literature search

Supplementary evidence was curated from published literature to complement and contextualize the experimental data generated within this study and to support the development of the DEHP Adverse Outcome Pathway Network (AOPN).

Literature searches were conducted using PubMed and Web of Science, following a KE- and KER-driven strategy (Villeneuve et al., 2014a; 2014b). Rather than applying a single predefined search string, literature searches were performed separately for each Key Event (KE) and Key Event Relationship (KER) included in the network (Wu et al., 2021). Search terms were therefore adapted to the specific biological process, molecular target, pathway, or adverse outcome under consideration (e.g., lipid metabolism, mitochondrial dysfunction, oxidative stress, insulin signaling).

This targeted and iterative approach reflects standard practice in AOP development and was necessary to ensure mechanistic specificity and biological relevance across different levels of biological organization (Watanabe et al., 2011). Retrieved studies were screened for relevance to DEHP exposure and for their ability to support biological plausibility, empirical evidence, or consistency of individual KEs and KERs (Villeneuve et al., 2014b; Pittman et al., 2018).

The curated literature was used to (i) support the interpretation of experimental findings, (ii) strengthen Weight-of-Evidence considerations for individual KEs and KERs, and (iii) ensure coherence of the proposed mechanistic network. Given the event-specific nature of the searches, exhaustive reporting of individual search strings was not considered feasible or informative.

## 3 Results

The results presented here delineate a mechanistically coherent sequence of events linking DEHP exposure to metabolic dysfunction, reconstructed through an integrative Adverse Outcome Pathway Network (AOPN) approach. This network synthesis is based on the combined interpretation of experimental data generated in this study (transcriptomic and metabolomic analyses), complementary systems biology modelling, and targeted integration of supporting *in vitro* and *in vivo* evidence from the OBERON project and the published literature.

To facilitate interpretation of the complex datasets, the AOP network is presented upfront as a top-down integrative framework that combines the experimental multi-omics and systems biology findings with established mechanistic evidence from the literature, thereby providing a structured context for the detailed results presented below.

Integration of multi-omics data across the investigated models enabled the reconstruction of a detailed molecular network, in which free fatty acid (FFA) dysregulation, mitochondrial dysfunction, serine/sphingosine-1-phosphate (S1P) pathway disruption and impaired insulin signaling emerged as central processes associated with metabolic toxicity (Figure 1). Complementary systems biology modelling further contextualized these proposed mechanistic relationships by mapping the omics-derived perturbations within metabolic pathways and characterizing the dose–response behavior of key metabolic intermediates.

**FIGURE 1:**
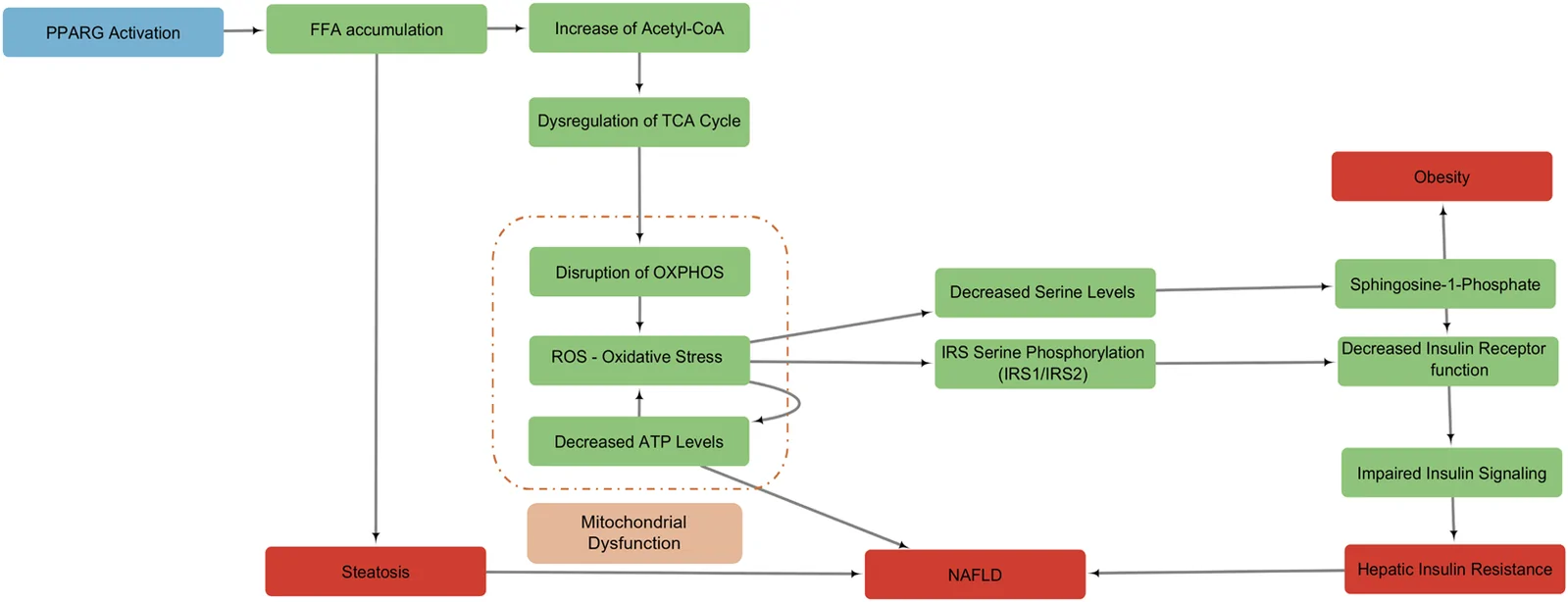
Adverse Outcome Pathway Network (AOPN) of DEHP-induced metabolic disruption, integrating multi-omics data and systems biology modeling. Central mechanistic clusters include mitochondrial dysfunction, oxidative stress, impaired insulin signaling, and serine/S1P dysregulation, proposed to contribute to steatosis, hepatic insulin resistance, NAFLD, and obesity. [Color coding indicates event type: molecular initiating event (MIE, blue), key events (KEs, green), and adverse outcomes (AOs, red).] Feedback loops are represented as directed cycles formed by multiple KERs; individual arrows indicate the direction of each pairwise relationship.

Detailed descriptions of individual Key Events (KEs), Key Event Relationships (KERs), associated Weight-of-Evidence (WoE) evaluations, and the underlying experimental and literature support are provided in Supplementary Material S1. Following OECD guidance (OECD, 2018), biological plausibility reflected the extent to which each proposed relationship was supported by established mechanistic knowledge. Empirical support was evaluated through dose–response and temporal concordance, while consistency reflected agreement across omics layers, experimental models, and independent studies; divergent findings were considered sources of uncertainty. Essentiality was assessed from available evidence that modification of the upstream KE altered the downstream response. In the network representation, each arrow denotes a directional relationship from an upstream KE to a downstream KE. Feedback loops are represented as directed cycles formed by multiple KERs rather than as single bidirectional relationships. Individual KERs therefore retain their upstream-to-downstream interpretation, while the complete network captures how downstream events may subsequently reinforce earlier events and amplify or sustain the response.

### 3.1 Multi-omics integration and identification of key events

To investigate the mechanistic basis of DEHP-induced metabolic disruption, we employed a multi-omics approach integrating untargeted transcriptomic and metabolomic analyses. Transcriptomic profiling revealed coordinated modulation of genes regulating lipid metabolism, specifically free fatty acid (FFA) handling, including PPARG and multiple PPARG-associated downstream targets such as, PPARGC1A, CIDEB/CIDEC, and CPT1A, consistent with altered hepatic lipid handling characterized by increased lipolysis, impaired lipid storage, and enhanced β-oxidation in HepaRG models. These coordinated changes are interpreted as reflecting increased intracellular FFA availability (KE1) together with compensatory mitochondrial fatty-acid utilization leading to increased acetyl-CoA (KE2). In this context, KE1 refers to increased intracellular FFA availability (pool size and/or flux), which may co-occur with enhanced β-oxidation as an adaptive response to elevated lipid load.

Mitochondrial function-related pathways were markedly affected, with DEHP exposure inducing expression of mitochondrial UCP3 and promoting mitochondrial ROS generation, accompanied by ROS-mediated activation of Nrf2-dependent stress responses and by increased expression of antioxidant enzymes such as *gpx4a* in zebrafish and GSTA2 in HepaRG 3D cells, aligning with oxidative stress (KE5) and mitochondrial dysfunction (KE7).

Metabolomic analysis revealed perturbation of TCA cycle intermediates such as citrate, succinate, and oxaloacetate (KE3). Transcriptomic analysis further identified reduced expression of the Complex I subunit NDUFA6 and the ATP-synthase subunit ATP5MC3 in EndoC-βH1 cells, providing molecular evidence consistent with disruption of mitochondrial energy metabolism and OXPHOS capacity (KE4).

Pathway analysis confirmed dysregulation of mitochondrial metabolism, with notable perturbations in the TCA cycle (KE3), indicated by altered levels of citrate and succinate, and fatty acid oxidation pathways (KE2). These multi-omics data layers were systematically mapped to key metabolic pathways, enabling the development of a proposed mechanistic sequence connecting these perturbations with adverse metabolic outcomes.

#### 3.1.1 FDR-controlled sensitivity analysis of transcript- and pathway-level findings

At the individual-gene level across the eight DEHP contrasts, between 125 and 3,497 transcripts pass the magnitude threshold, 47 to 572 additionally pass a nominal p < 0.05, and only 3 transcripts in the entire study (all in HepaRG at 100 pM and 100 nM) survive BH-FDR < 0.05. These three are CYP1A1, CYP1A2 and CYP1B1, xenobiotic-metabolising enzymes rather than members of any key-event pathway. None of the individual genes named in the Results, including PPARG, PPARGC1A, CIDEB/CIDEC, the perilipins, CS, FH, SDHC, IRS1 and UCP3, reaches gene-level FDR significance; the smallest adjusted p-value among them is 0.67. This pattern reflects a three-to four-replicate design in a 15,000–22,000-dimensional testing problem.

Set-level testing aggregates evidence across the genes constituting each pathway and is better powered at this replicate number. Competitive gene-set tests (CAMERA, inter-gene correlation 0.01) against KEGG pathway definitions retained 24 of 72 model-by-dose-by-pathway tests at BH-FDR < 0.05 within contrast, and 23 when correction is applied globally across the whole family of tests. Six of the nine pathways carrying key events are significant in at least one system after FDR correction: oxidative phosphorylation (minimum FDR 4.3 × 10^−10^), fatty acid metabolism (7.3 × 10^−8^), PPAR signalling (6.2 × 10^−6^), the citrate (TCA) cycle (3.5 × 10^−3^), glycolysis/gluconeogenesis (3.5 × 10^−3^) and glycine/serine/threonine metabolism (1.9 × 10^−3^). Together these carry the MIE and key events KE1 to KE8 and KE14, that is the mitochondrial and lipid-handling core of the network, and each is significant in three to five independent contrasts rather than in a single one. Three pathways do not reach the corrected threshold: sphingolipid metabolism shows consistent low-dose hepatic induction but attains only FDR = 0.15, the insulin signalling pathway falls just short at FDR = 0.053 with a coherent zebrafish suppression at both concentrations, and type II diabetes mellitus, a composite disease annotation rather than a mechanistic pathway, attains FDR = 0.37. Support for the S1P and insulin key events (KE10 to KE12) therefore rests on the directly measured metabolomic evidence and on the insulin-secretory endpoints measured within OBERON rather than on transcriptomic set-level significance, and Supplementary Material S1 flags them accordingly. At the level of the joint pathway analysis reported in the Results, 48 of 408 enriched pathways retain FDR < 0.05, including the zebrafish TCA cycle (FDR = 0.021) and oxidative phosphorylation (FDR = 3.1 × 10^−23^).

These findings indicate that individual transcripts and metabolites should be interpreted as illustrative annotations of pathway-level signals rather than as independently significant findings. Accordingly, individual log_2_FC values are reported to document response direction and magnitude but are not interpreted as statistically significant in isolation. Support for the network KEs and KERs is evaluated primarily at pathway or gene-set level. The full refitted statistics (per-gene logFC, p and BH-FDR for all contrasts), the CAMERA gene-set results and the attrition counts are summarised in Figure 2.

**FIGURE 2:**
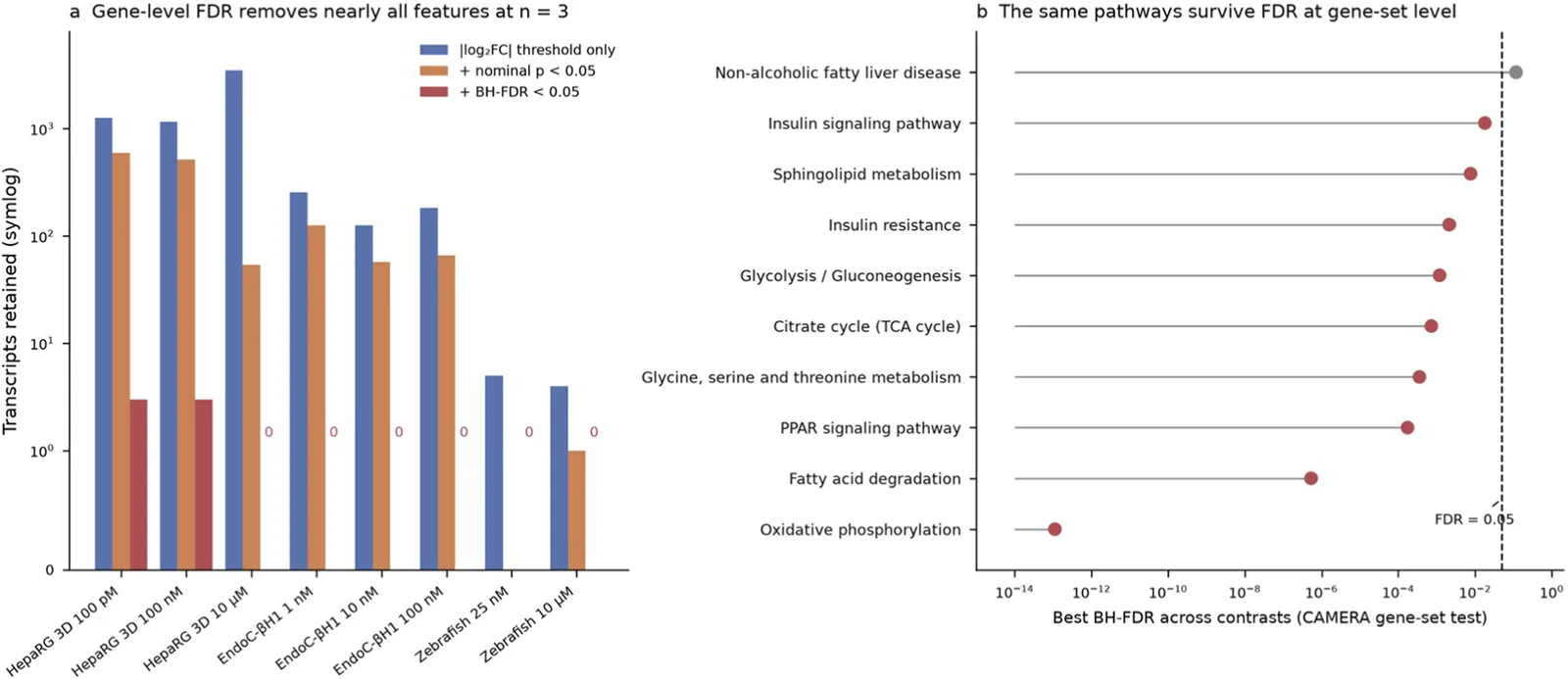
Sensitivity of feature selection to false-discovery-rate control. **(a)** Transcripts retained in each DEHP contrast under three successively stricter criteria: the magnitude threshold alone (|log_2_FC| ≥ 0.58 for cell models, ≥ 1.3 for zebrafish), magnitude plus nominal p < 0.05, and magnitude plus Benjamini-Hochberg FDR < 0.05; counts above the third bar give the number surviving FDR. The y-axis is symmetrical-log to keep zero counts visible. **(b)** Best BH-adjusted p-value achieved by each key-event pathway across all contrasts in competitive gene-set testing (CAMERA); the dashed line marks FDR = 0.05 and the x-axis is logarithmic. n = 3 biological replicates per condition in the human cell systems and n = 4 in zebrafish. Individual transcripts almost never survive FDR at this sample size, whereas the pathways carrying the network’s key events do, which is why key events are supported at pathway rather than single-gene level.

### 3.2 Systems biology modeling and network inference

To contextualize the omics-derived molecular perturbations within a mechanistic framework (Karakoltzidis, 2026), we employed an established computational systems biology model of hepatic carbohydrate and lipid metabolism. Specifically, the experimentally measured changes in palmitate levels in HepaRG 3D cells, following DEHP exposure (Figure 3) were used to parameterize the systems biology model, capturing changes in fatty-acid handling rather than total lipid content. The observed palmitate depletion was considered consistent with the redistribution of fatty-acid flux toward ceramides and related lipotoxic lipid species, as captured in KER2 and KER11 of the AOP network, while sustained fatty-acid pressure at the network level may contribute to downstream steatotic outcomes.

**FIGURE 3:**
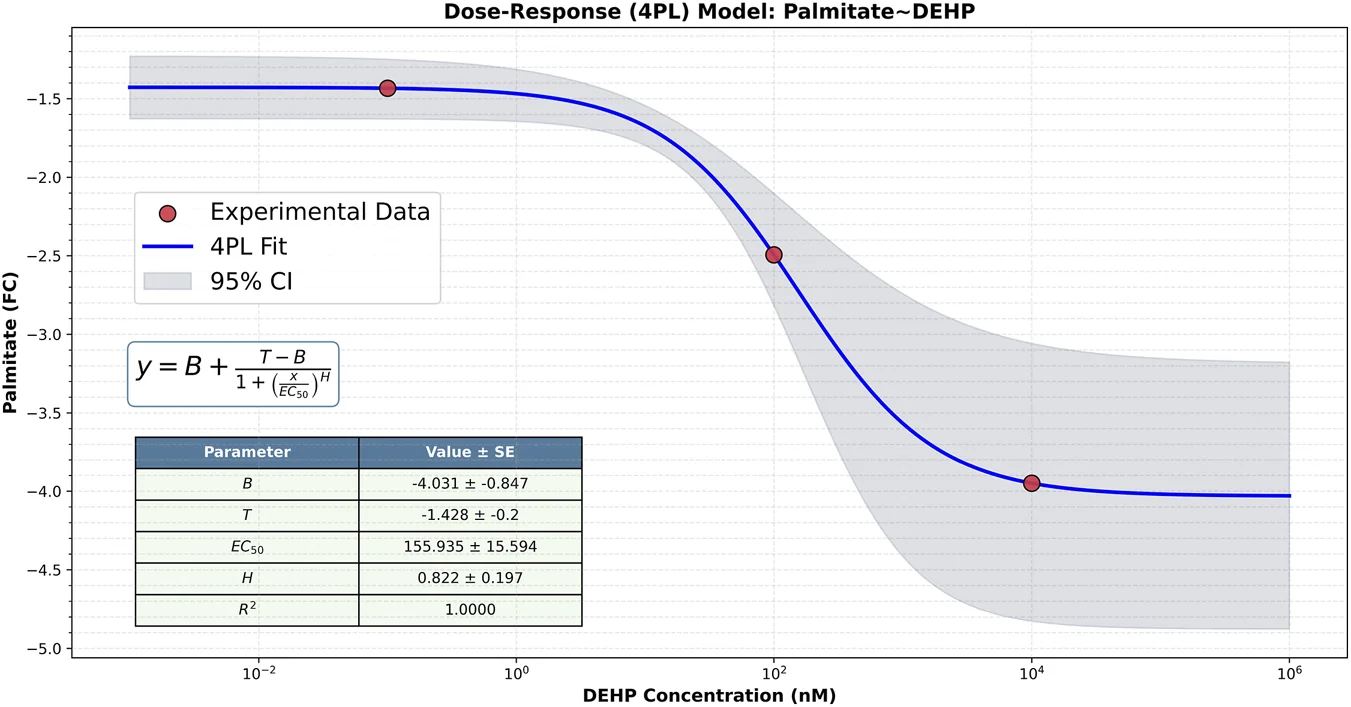
Dose–response relationship between DEHP concentration and palmitate levels. Palmitate concentrations measured by metabolomics in HepaRG cells following DEHP exposure (red circles) were fitted with a four-parameter logistic (4PL) model (blue curve) with 95% confidence intervals (gray shading). B represents the bottom asymptote, T the top asymptote, EC_50_ the concentration producing a half-maximal response, and H the Hill slope.

Following reparameterisation using the experimentally observed fatty-acid alterations, the model predicted an increase in acetyl-CoA concentration (KE2; Figure 4), consistent with a shift toward β-oxidation under excess fatty-acid availability. This model-predicted increase in acetyl-CoA was associated with increased metabolic pressure on the TCA cycle (KE3); however, rather than increasing flux, this state is characterized by a functional TCA bottleneck in which accumulation of reducing equivalents and electron pressure limits efficient oxidative phosphorylation. This interpretation was considered together with the observed downregulation of rate-limiting enzymes, including citrate synthase, fumarate hydratase, and succinate dehydrogenase, observed across the experimental models. Such restriction in cycle capacity is consistent with elevated mitochondrial redox pressure, promoting electron leakage and reactive oxygen species production. Aligned with this mitochondrial stress state, the model predicted a reduction in ATP concentration (KE6; Figure 4). This prediction was consistent with the downregulation of ATP5MC3 captured in the omics data and consistent with downstream oxidative stress/ROS responses captured by the omics data (KE5).

**FIGURE 4:**
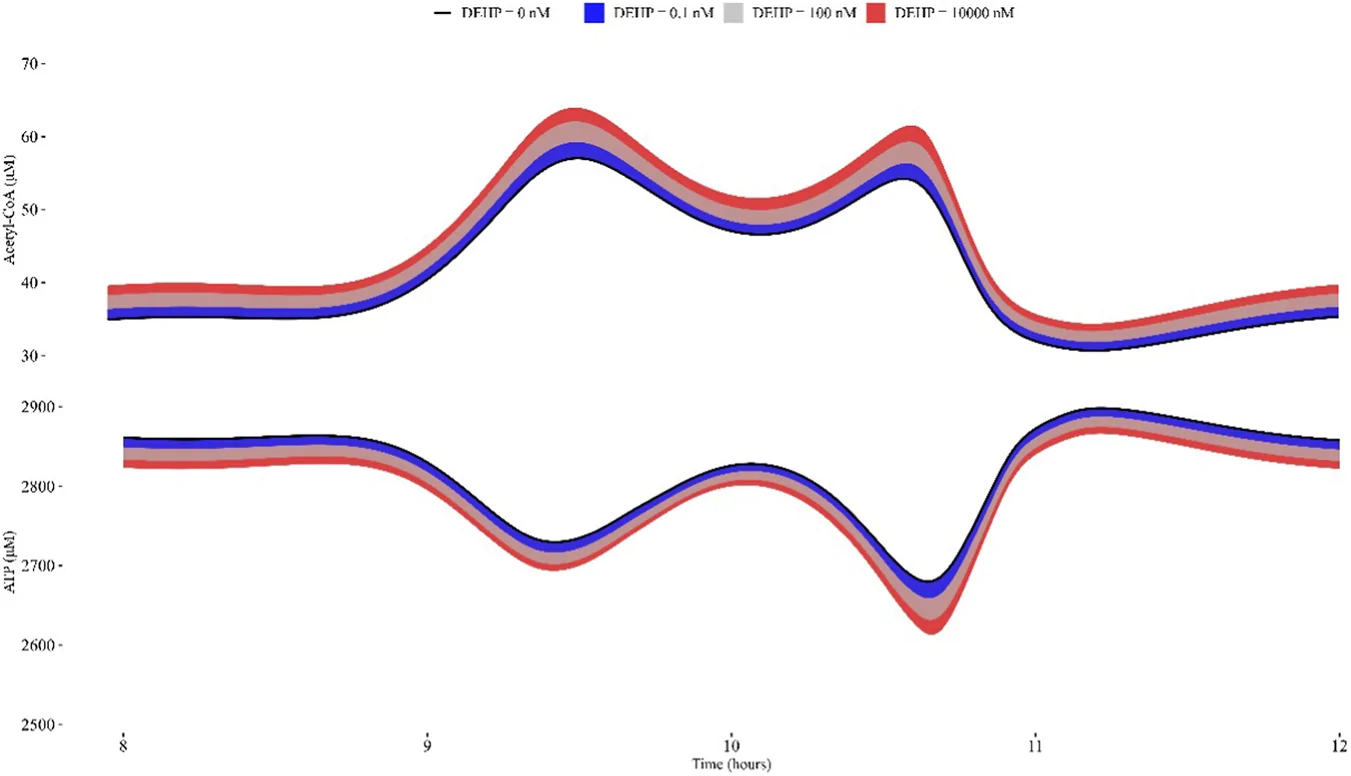
Model-predicted acetyl-CoA and ATP concentrations following DEHP exposure. Curves are simulated concentration–time trajectories from the systems biology model, in which each DEHP concentration is represented by a scaling of the *de novo* lipogenesis rate constant fitted to the measured palmitate dose–response (Figure 3); band width denotes the periportal-to-pericentral range across the eight sinusoidal compartments. Colours denote DEHP concentration: black 0 nM, blue 0.1 nM, grey 100 nM, red 10,000 nM. The 8–12 h window is not an experimental exposure duration and does not indicate a 4 h treatment: it is the interval of the simulated 24 h feeding–fasting cycle spanning one postprandial substrate spike and the subsequent interprandial phase. It is shown because the separation between exposure levels is greatest under the metabolic load imposed by a feeding transient and is negligible at steady state, so this window is the most informative view of the predicted effect. Read at any fixed time point within it, the curves are ordered monotonically by DEHP concentration, with predicted acetyl-CoA increasing and predicted ATP decreasing as concentration rises, and the separation between exposure levels maximal at the postprandial peak. These are model predictions constrained by a single fitted perturbation; acetyl-CoA and ATP were not measured experimentally in this study, and the absolute values should not be interpreted as measurements.

Excess ROS is in turn expected to engage oxidative stress response pathways such as the Nrf2-Keap1 axis, and the observed induction of antioxidant enzymes (gpx4a, GSTs), is consistent with this (KE5). Sustained oxidative pressure together with ATP depletion is proposed to lead to mitochondrial dysfunction (KE7), reinforcing ROS generation in a self-sustaining feedback loop consistent with KER4 (TCA overload → disruption of OXPHOS), KER5 (disruption of OXPHOS → ATP depletion), and KER7 (ATP depletion → ROS). In this cycle, the individual KERs retain their directional interpretation, while completion of the cycle allows energetic and oxidative stress to reinforce earlier mitochondrial events. Together, these simulations are directionally concordant with the omics data and illustrate how early metabolic disruptions could propagate toward downstream cellular dysfunction; because acetyl-CoA and ATP were not measured, this concordance is interpreted as mechanistic support rather than quantitative validation. The evidence from the systems biology model is summarized in Figure 4.

### 3.3 Mechanistic progression of key events leading to metabolic dysfunction

#### 3.3.1 PPARγ activation and lipid accumulation

Exposure to DEHP was associated with upregulation of PPARγ, a nuclear receptor regulating lipid metabolism, in HepaRG cells at low concentrations (log_2_FC0.1nM_LU_ = 0.589, log2FC0.1nM_GU_ = 0.705). Concurrent suppression of its co-activator PPARGC1A, which supports mitochondrial biogenesis and fatty acid oxidation, was consistent with a shift in hepatic lipid handling toward storage-dominant metabolism relative to coordinated mitochondrial oxidation, despite evidence of compensatory β-oxidation under excess fatty-acid availability. This coordinated transcriptional response was interpreted as being consistent with an increased intracellular free fatty acid (FFA) pool, marking the initiation of KE1 (FFA accumulation) (Figure 5).

**FIGURE 5:**
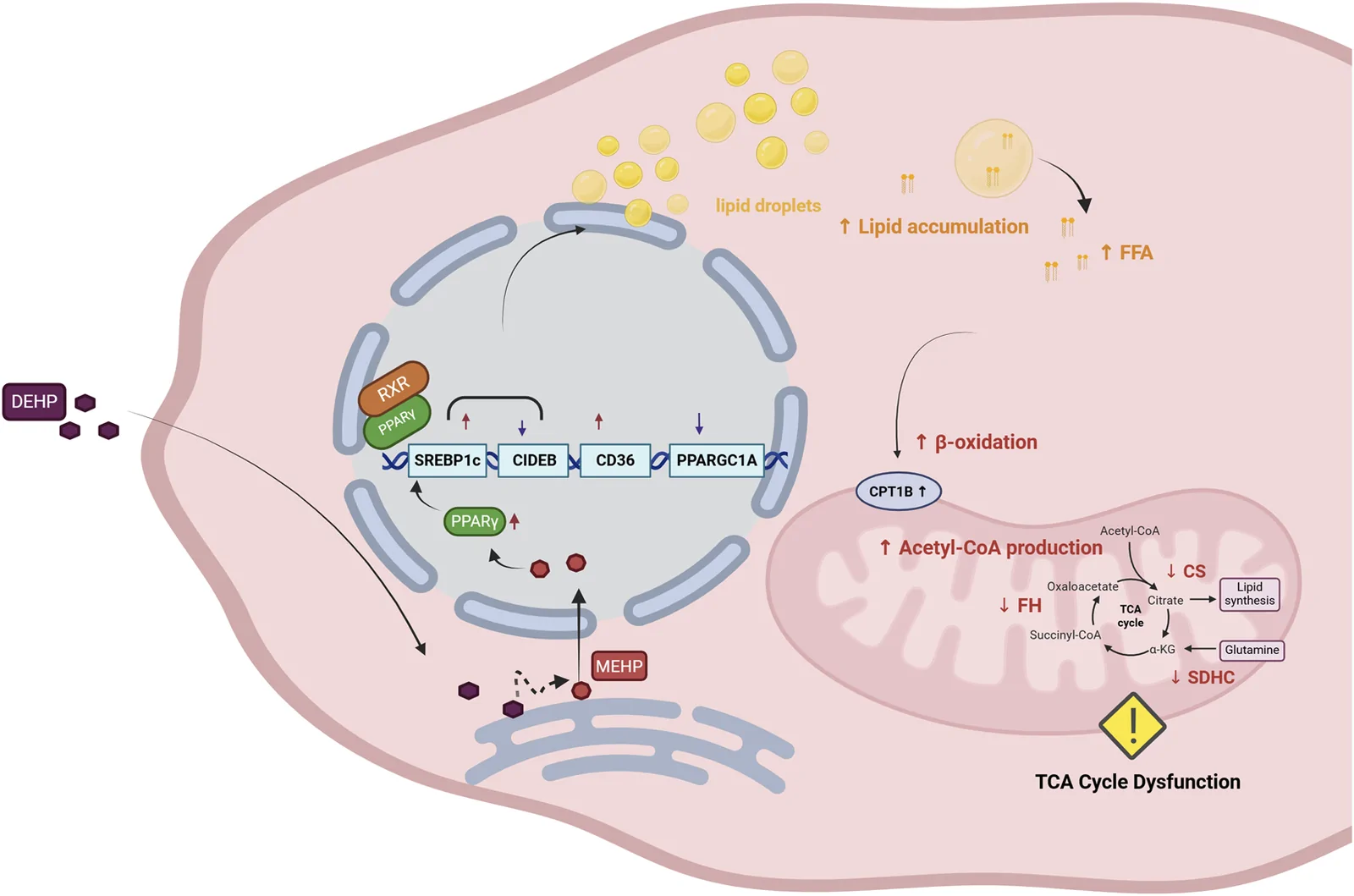
Early Molecular Events Induced by DEHP in HepaRG Cells. DEHP activates PPARγ/RXR signaling, altering lipid-regulatory and lipid droplet–associated genes expression (including SREBP1c, CD36, CIDEB/CIDEC). The suppression of PPARGC1A, combined with the downregulation of droplet stabilizers like CIDEB/CIDEC and perilipins (PLIN1/2), is consistent with impaired triglyceride storage and increased intracellular FFA availability (KE1). This mismatch between lipid influx and storage sequestration reflects destabilization of lipid droplets and enhanced mobilization of FFAs into the cytoplasm. These FFAs subsequently enter the mitochondria via CPT1B as a compensatory response, enhancing β-oxidation and acetyl-CoA production (KE2). This substrate surge is associated with a functional TCA bottleneck (KE3), supported by reduced expression of CS, FH, and SDHC, which marks the onset of early mitochondrial dysfunction. Abbreviations: DEHP, Di(2-ethylhexyl) phthalate; PPARγ, peroxisome proliferator-activated receptor gamma; RXR, retinoid X receptor; FFA, free fatty acid; CPT1B, carnitine palmitoyltransferase 1B; CS, citrate synthase; FH, fumarate hydratase; SDHC, succinate dehydrogenase complex subunit C; KE, key event; KER, key event relationship.

Transcriptomic data further showed downregulation of lipid droplet regulators CIDEB and CIDEC (FSP27) (log2FC10µM = −0.71 and −0.82), consistent with reduced lipid-droplet stability and altered neutral-lipid storage, primarily triglycerides, within the droplets. Concurrently, perilipins (PLIN1, PLIN2), essential for lipid droplet stability, were also reduced (log2FC10µM = −0.68 and −1.15), collectively facilitating enhanced lipolysis. This enzymatic mobilization of neutral lipids by upregulated key lipolytic enzymes such as adipose triglyceride lipase (ATGL, log2FC100nM = 0.61) and hormone-sensitive lipase (HSL, log2FC10µM = 1.02), were interpreted as being consistent with the expansion of the intracellular free fatty acid pool, even as the concurrent downregulation of monoglyceride lipase (log2FC10µM = −1.47) points to a specific imbalance in the final stage of lipid turnover. This coordinated shift in droplet stability and lipolytic enzyme expression suggests increased FFA availability, supplying the substrates for subsequent β-oxidation and acetyl-CoA generation.

Thus, the combined changes in PPARG, PPARGC1A, and lipid-droplet-related genes were interpreted as supporting a proposed sequence involving FFA accumulation (KE1), supplying substrates for β-oxidation and acetyl-CoA generation (KE2) and propagating downstream metabolic stress in hepatocytes. In this context, the model-predicted increase in acetyl-CoA is interpreted as reflecting a substrate-driven metabolic overload arising from excess free-fatty-acid availability and compensatory β-oxidation following PPARγ activation, rather than reflecting a primary consequence of reduced ATP production; when acetyl-CoA generation exceeds TCA cycle and OXPHOS processing capacity, mitochondrial stress ensues, leading secondarily to impaired oxidative phosphorylation and ATP depletion (Figure 4).

#### 3.3.2 TCA cycle overload and mitochondrial dysfunction

Following FFA accumulation, an increase in β-oxidation (KER2) is proposed to raise acetyl-CoA production (KE2). While initially compensatory, acetyl-CoA generation exceeding the processing capacity of the tricarboxylic acid (TCA) cycle would culminate in TCA cycle overload (KE3); acetyl-CoA was not measured in this study and this step rests on the model prediction together with the transcriptional evidence below. Rather than a restorative regulatory shift, the resulting reduction in the expression of rate-limiting enzymes, including citrate synthase (CS), fumarate hydratase (FH), and succinate dehydrogenase complex subunit C (SDHC), as shown in HepaRG cells (Figure 6) and zebrafish larvae, was interpreted as being consistent with restricted TCA-cycle capacity. Within the proposed network, this downregulation may contribute to a self-reinforcing cycle, in which accumulation of acetyl-CoA, mitochondrial protein hyperacetylation and feedback inhibition of dehydrogenases further constrict the cycle’s processing capacity. This feed-forward loop exacerbates the initial metabolic bottleneck, driving the system toward the sustained mitochondrial stress and energetic imbalance.

**FIGURE 6:**
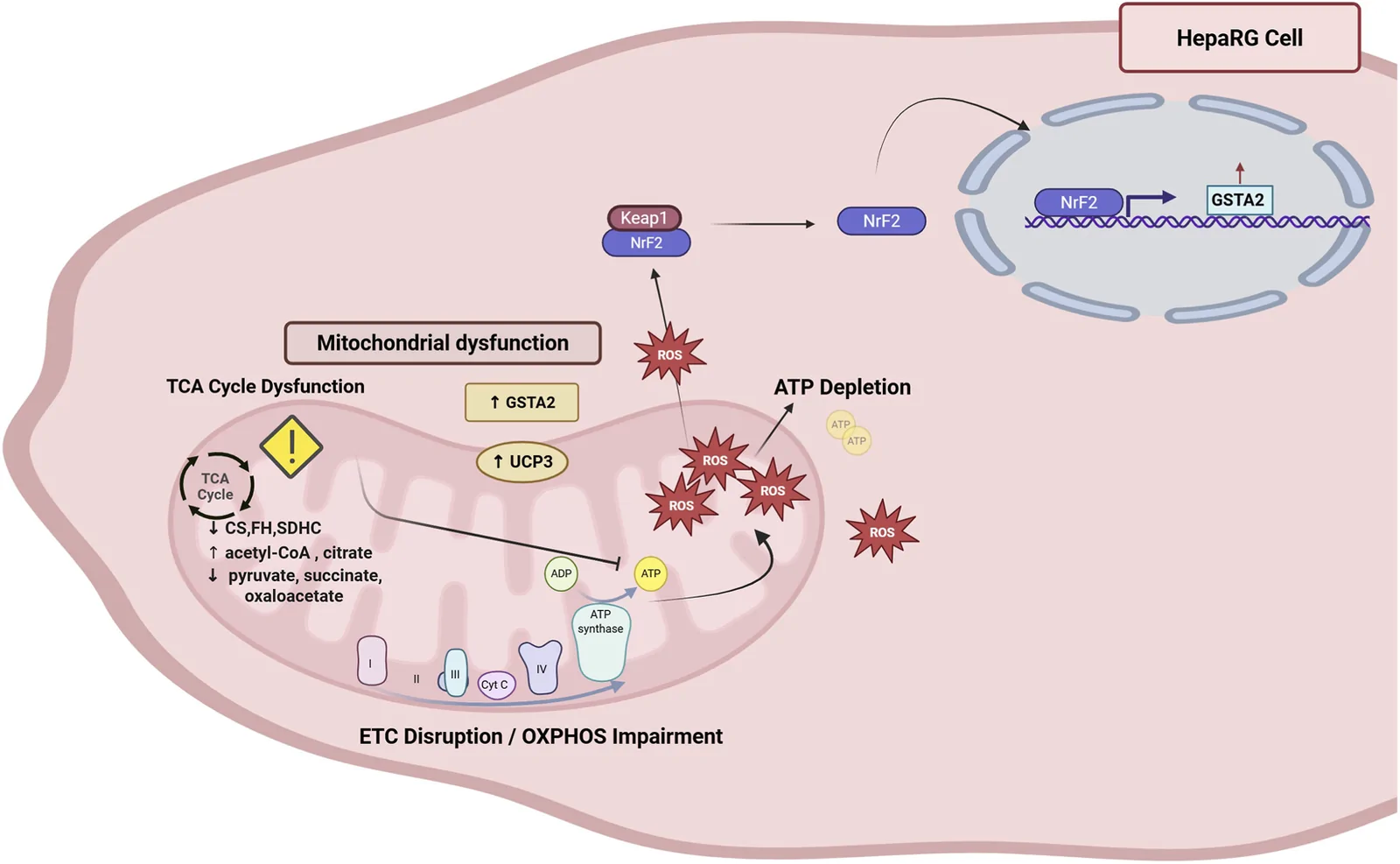
Mitochondrial dysfunction and Nrf2-mediated stress response in HepaRG cells. DEHP-induced metabolic perturbations (KE3) are associated with impaired oxidative phosphorylation (KE4) and constrained electron transport chain (ETC) function, promoting increased mitochondrial ROS generation (KE5). Elevated ROS activates the Keap1–Nrf2 pathway, resulting in induction of antioxidant genes (GSTA2; KE5). Concomitantly, disruption of OXPHOS efficiency (KER5) is associated with reduced ATP synthesis (KE6). Increased ROS further contributes to mitochondrial energetic stress (KER6), while ATP depletion may reinforce ROS production and mitochondrial dysfunction (KER7), forming a self-amplifying stress loop. This figure illustrates the interconnected mitochondrial stress module downstream of early metabolic disturbances. Gene symbols are shown only where supported by human 3D HepaRG data; zebrafish-specific markers (e.g., *gpx4a*) are integrated at the key-event level and are detailed in Supplementary Material S1. Abbreviations: DEHP, di(2-ethylhexyl) phthalate; ETC, electron transport chain; OXPHOS, oxidative phosphorylation; ROS, reactive oxygen species; Keap1, Kelch-like ECH-associated protein 1; Nrf2, nuclear factor erythroid 2-related factor 2; GSTA2, glutathione S-transferase alpha 2; UCP3, uncoupling protein 3; ATP, adenosine triphosphate; KE, key event; KER, key event relationship.

Metabolomic profiling identified altered TCA-cycle intermediates, such as decreased oxaloacetate, pyruvate, and succinate, while systems biology modeling predicted increased acetyl-CoA. This pattern is consistent with altered mitochondrial redox balance and redox pressure on the electron transport chain, thereby promoting electron leakage and enhanced ROS production. In parallel, transcriptomic data indicated activation of stress-responsive signaling pathways, including protein kinase C (PKC) (log2FC10µM = 0.60) and NADPH oxidase (NOX) (log2FC10µM = 0.80), which may contribute to ROS amplification. Electron leakage is interpreted as contributing to reactive oxygen species (ROS) production (KE5) (Figure 6); mitochondrial ROS were not measured directly in this study. Mitochondrial stress responses were engaged as reflected by upregulation of antioxidant enzymes such as *gpx4a* in zebrafish and GSTA2 in HepaRG cells, consistent with activation of the Nrf2-mediated oxidative stress response (KE5). Transcriptomic data further revealed upregulation of UCP3 in HepaRG cells, suggesting a compensatory mitochondrial uncoupling response aimed at limiting excessive ROS formation under conditions of OXPHOS stress. Although such adaptive responses may mitigate mitochondrial damage, they were insufficient to prevent sustained oxidative pressure and progressive mitochondrial dysfunction (KE7).

#### 3.3.3 Disrupted insulin signaling and glucose metabolism

DEHP exposure was associated with tissue-specific disruption of insulin signaling potentially through a coordinated cascade of oxidative stress–driven metabolic disturbances. Elevated ROS generated downstream of mitochondrial dysfunction (KER5–KER7) is proposed to inhibit the serine biosynthesis pathway by suppressing phosphoglycerate dehydrogenase (PHGDH), leading to reduced L-serine availability (KER9) (SM1). L-serine depletion, observed consistently in both HepaRG cells (log2FC0.1nMU = −1.102, log2FC100nMU = −1.372) and zebrafish larvae (log2FC10uMU = −1.729, log2FC25nMU = −1.777) may be responsible for decreased sphingolipid biosynthesis, and a potential contributor to dysregulation of sphingosine-1-phosphate (S1P) homeostasis (KER11) (Figure 7).

**FIGURE 7:**
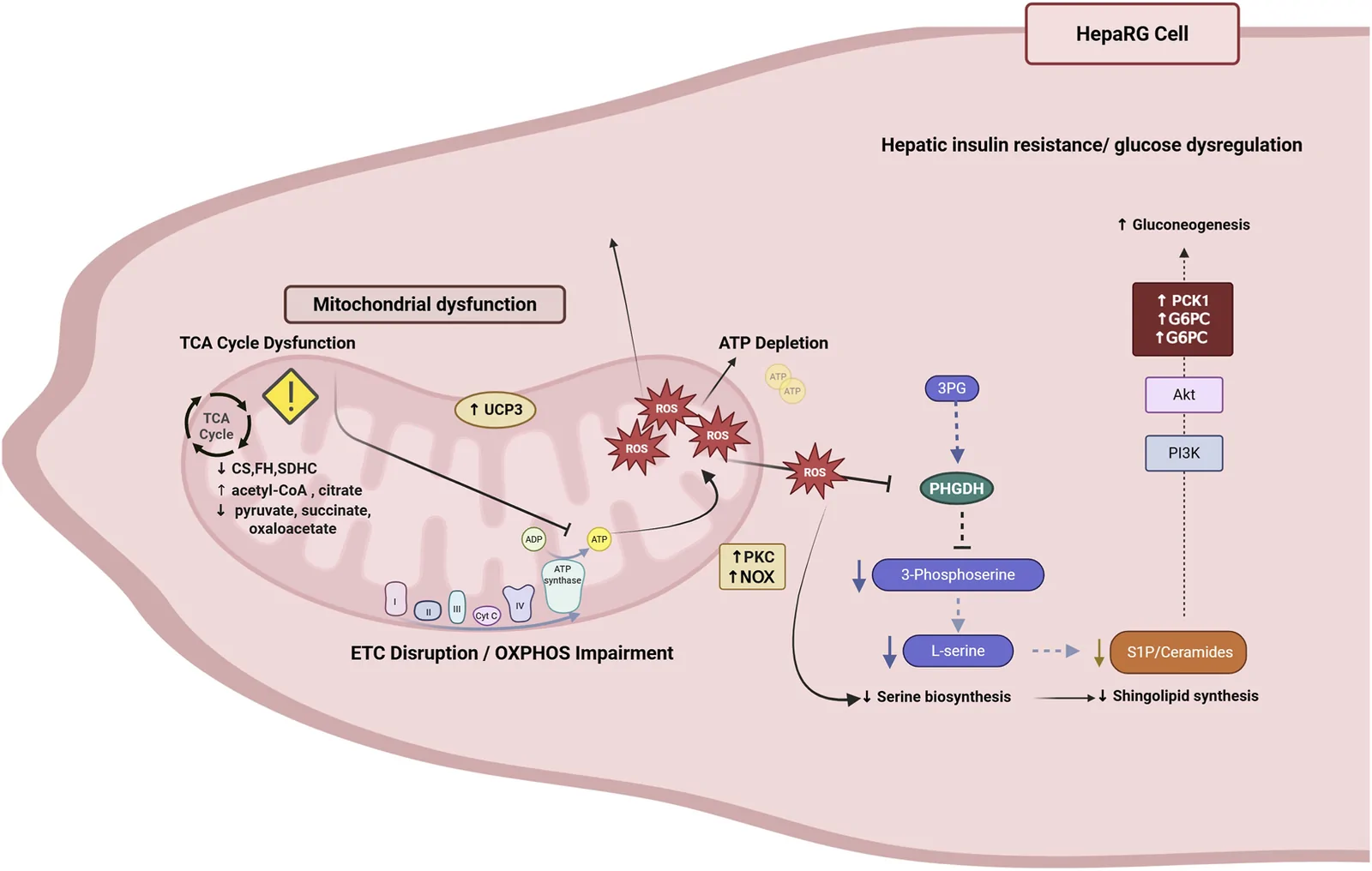
Hepatic metabolic perturbations linking mitochondrial stress, altered serine–sphingolipid metabolism, and gluconeogenic gene induction in HepaRG cells. In HepaRG 3D cells, DEHP exposure induces mitochondrial dysfunction and ETC/OXPHOS impairment (KE4), leading to increased reactive oxygen species (ROS; KE5) and ATP depletion (KE6). Oxidative stress is associated with inhibition of PHGDH and reduced serine biosynthesis, reflected by decreased 3-phosphoserine and L-serine levels (KE8), accompanied by altered sphingolipid metabolism, including changes in S1P species and increased ceramides (KE11). These lipid alterations are linked to increased expression of gluconeogenesis-related genes (PCK1, G6PC), contributing to hepatic insulin resistance (KE14) and glucose dysregulation. Impairment of PI3K/Akt signaling is shown as a mechanistically supported link (KER) connecting sphingolipid imbalance to dysregulated gluconeogenic control. Abbreviations: DEHP, di(2-ethylhexyl) phthalate; ETC, electron transport chain; OXPHOS, oxidative phosphorylation; ROS, reactive oxygen species; ATP, adenosine triphosphate; 3PG, 3-phosphoglycerate; PHGDH, phosphoglycerate dehydrogenase; S1P, sphingosine-1-phosphate; PCK1, phosphoenolpyruvate carboxykinase 1; G6PC, glucose-6-phosphatase catalytic subunit; PI3K, phosphoinositide 3-kinase; Akt, protein kinase B; KE, key event; KER, key event relationship.

S1P profiling in 3D HepaRG cells showed a reduction in the C16 S1P species (log2FC100pMLU = −4.22, log2FC100nMLU = −3.75, log2FC10uMLU = −2.47) and an increase in the C19 species (log2FC100pMLU = 0.253, log2FC100nMLU = 1.72, log2FC10uMLU = 1.11) following DEHP exposure, indicating chain-length-specific remodelling of the S1P profile rather than a uniform increase or decrease in total S1P. Considered together with the reductions in serine and palmitate and the increases in several long-chain ceramide species, this pattern supports redistribution of the sphingolipid pool under DEHP exposure. Such remodelling is biologically relevant because sphingolipid balance contributes to membrane organisation, inflammatory signalling, insulin signalling, and metabolic homeostasis. Accordingly, KE11 is defined as S1P disruption rather than increased or decreased total S1P. Altered S1P homeostasis may contribute to inflammatory signalling and impaired insulin-receptor function, as described in the weight-of-evidence assessment for KER13 (SM1), although the specific downstream functions of the individual C16 and C19 species were not directly assessed.

In parallel, oxidative and inflammatory stress arising from mitochondrial dysfunction, as described above, are known to induce serine phosphorylation of IRS1/IRS2 (KER10), a well-established mechanism of insulin resistance in insulin-responsive tissues such as liver, muscle, and adipose tissue. This post-translational modification is expected to impair autocrine insulin receptor downstream signaling (KER12: IRS serine phosphorylation → Decreased insulin receptor function). In parallel, a modest reduction in IRS1 expression was observed in pancreatic β‐cells (log2FC −0.27). Experimental data from the OBERON project (Al-Abdulla et al., 2022) further demonstrated that DEHP exposure led to decreased insulin secretion and insulin content, consistent with β-cell dysfunction. It should be noted that, unlike classical insulin-responsive tissues, these effects in β-cells primarily impact β-cell function and survival rather than systemic insulin resistance (Figure 8).

**FIGURE 8:**
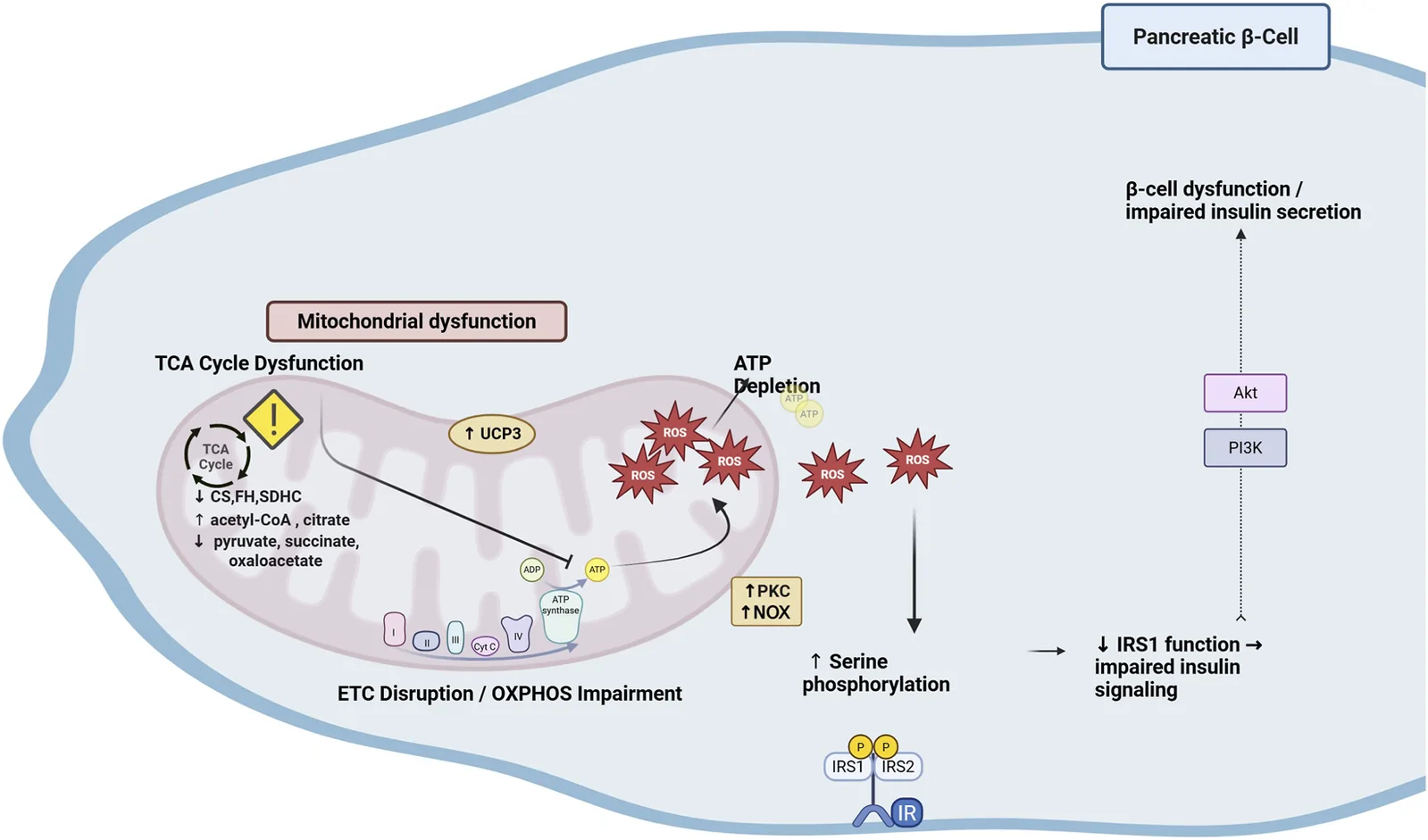
Mitochondrial stress–associated β-cell dysfunction. DEHP induces mitochondrial dysfunction and ETC/OXPHOS impairment (KE4), resulting in increased ROS (KE5) and ATP depletion (KE6). Oxidative stress activates stress-responsive serine/threonine kinases, resulting in inhibitory serine phosphorylation of insulin receptor substrates (IRS1/IRS2) (KE9). This attenuates downstream IRS–PI3K/Akt signaling, which in β-cells is involved in the regulation of β-cell function, stress responses, and cell survival, ultimately contributing to impaired insulin secretion and β-cell dysfunction. Abbreviations: IRS1/2, insulin receptor substrates 1 and 2; PI3K–phosphoinositide 3-kinase; Akt–protein kinase B; ETC, electron transport chain; OXPHOS, oxidative phosphorylation.

The combined impact of IRS1/IRS2 dysregulation (Figure 8) and S1P imbalance (Figure 7) potentially resulted in decreased insulin receptor function (KE10), disrupting the PI3K/Akt pathway which may be critical for β-cell autocrine signaling and insulin secretion. Importantly, transcriptomic data from Hepoid with HepaRG 3D cells showed increased expression of gluconeogenic regulators PCK1, G6PC, and FOXO6, indicating a metabolic shift toward enhanced hepatic glucose production (Figure 7). This mechanistic progression is consistent with KER15, where decreased insulin receptor activity leads to impaired insulin signaling. Within the proposed AOP network, this illustrates how integration of pathway-level evidence across systems can support a mechanistic sequence leading to systemic glucose intolerance.

As a consequence of these upstream disturbances, hepatocytes displayed increased gluconeogenic gene expression, consistent with impaired hepatic insulin signaling (KER17) (Figure 6) and contributing to systemic glucose intolerance. Collectively, these findings support a proposed convergence of mitochondrial dysfunction, oxidative stress, serine depletion, and S1P dysregulation on pathways relevant to impaired insulin signaling and promote metabolic dysfunction relevant to NAFLD and type 2 diabetes.

#### 3.3.4 Hepatic steatosis and non-alcoholic fatty liver disease

Hepatic lipid accumulation emerged as a central early event following DEHP exposure, driven by a mismatch between lipid influx and storage capacity. Excess free fatty acids (FFAs), proposed to arise through PPARγ-driven lipid remodeling (KE1), are expected to enter a compensatory β-oxidation pathway. Once acetyl-CoA generation exceeds the TCA cycle’s processing capacity (KE3), the surplus may be increasingly diverted toward triglyceride esterification, resulting in hepatocellular steatosis (KE13). This transition is consistent with KER16 (FFA Accumulation → Steatosis) and was supported by multi-omics evidence: DEHP-exposed HepaRG cells displayed marked downregulation of lipid droplet stabilizers CIDEB and CIDEC/FSP27, as well as perilipins PLIN1 and PLIN2, indicating impaired lipid droplet integrity and reduced capacity to buffer excess fatty acids, promoting a constant FFA spillover into the cytoplasm that maintains high levels of lipotoxic stress alongside TG accumulation. Steatosis may sensitize hepatocytes to oxidative and inflammatory stress, creating conditions favorable for disease progression. Elevated ROS levels, together with impaired mitochondrial function, enhanced lipid peroxidation and activated NF-κB signaling, molecular processes that may serve as parallel ‘hits’ driving the progression toward advanced NAFLD stages. These changes align with the notion that steatosis is necessary but not sufficient for progression of NAFLD, requiring additional “second hits” such as oxidative stress and inflammatory activation.

Persistent steatosis, coupled with this “leaky” storage and mitochondrial stress, was associated with hepatic insulin signaling dysfunction (KE14), as reflected by reduced insulin-mediated suppression of gluconeogenic programs. Consistent with KER17, this loss of coordinated insulin signaling occurred alongside activation of lipogenic regulators (SREBP-1c and PPARγ), contributing to further hepatic lipid accumulation and progression toward NAFLD.

The transition from steatosis to later stages of NAFLD (KE15) was further reinforced by DEHP-mediated activation of lipogenic and inflammatory pathways. Transcriptional evidence of LXR/SREBP-1c and NF-κB axis engagement and of PPARGC1A downregulation is consistent with enhancement of both lipid accumulation and inflammatory signaling. These mechanistic changes are consistent with KER19 (Steatosis → NAFLD) and with literature demonstrating DEHP-induced steatosis, inflammation, and early fibrotic changes in rodent and *in vitro* models (Chen et al., 2016; Bernal et al., 2024; Liang et al., 2024).

Together, these findings support a biologically coherent progression from FFA accumulation to steatosis and ultimately to NAFLD, driven by converging mechanisms of metabolic overload, oxidative stress, inflammation, and impaired insulin signaling.

#### 3.3.5 Obesity and metabolic outcomes

DEHP exposure was associated with lipid-metabolism perturbations relevant to pathways implicated in obesity (KE16). Within the AOPN structure, S1P disruption is proposed to contribute to obesity through KER14 (S1P Disruption → Obesity), whereby disruptions in sphingolipid signaling induced by reduced serine availability (KE8; KER11) contribute to metabolic inflexibility and shifts in energy homeostasis. In our multi-omics dataset, altered S1P species profiles, including decreased C16 S1P and elevated C19 S1P, reflect perturbations consistent with impaired lipid signaling dynamics. Such disturbances are known to influence systemic metabolic regulation and pathways relevant to obesity development, aligning with the role of S1P signaling in the development of obesity.

While obesity is mechanistically linked to S1P disruption in the AOPN, additional metabolic disturbances observed in DEHP-exposed models, such as PPARγ activation, impaired β-oxidation, and altered insulin responsiveness, provide a plausible mechanistic basis for how metabolic reprogramming may accompany or exacerbate obesity-related outcomes. Lipid accumulation, disrupted mitochondrial function, inflammatory signaling and decreased pancreatic β-cell function represent co-occurring features that contribute to an obesogenic metabolic environment, even though they do not constitute direct causal KERs leading to obesity in the present network. Collectively, these findings support the inclusion of altered sphingolipid signalling as a candidate upstream mechanism relevant to obesity (KE11), while broader DEHP-mediated metabolic disruptions create conditions that may potentiate an obesogenic metabolic environment and systemic metabolic dysfunction.

### 3.4 Conserved and divergent responses across the three experimental systems

Because the network draws on hepatic, pancreatic and whole-organism evidence, we assessed directly which responses are conserved across systems and which are model-specific. For each KEGG pathway carrying a key event, a directional pathway score was computed as the standardised mean moderated t-statistic of its member genes within each model-by-dose contrast, and significance was assessed by competitive gene-set testing with BH correction (Figure 9; full statistics in Supplementary Material S3).

**FIGURE 9:**
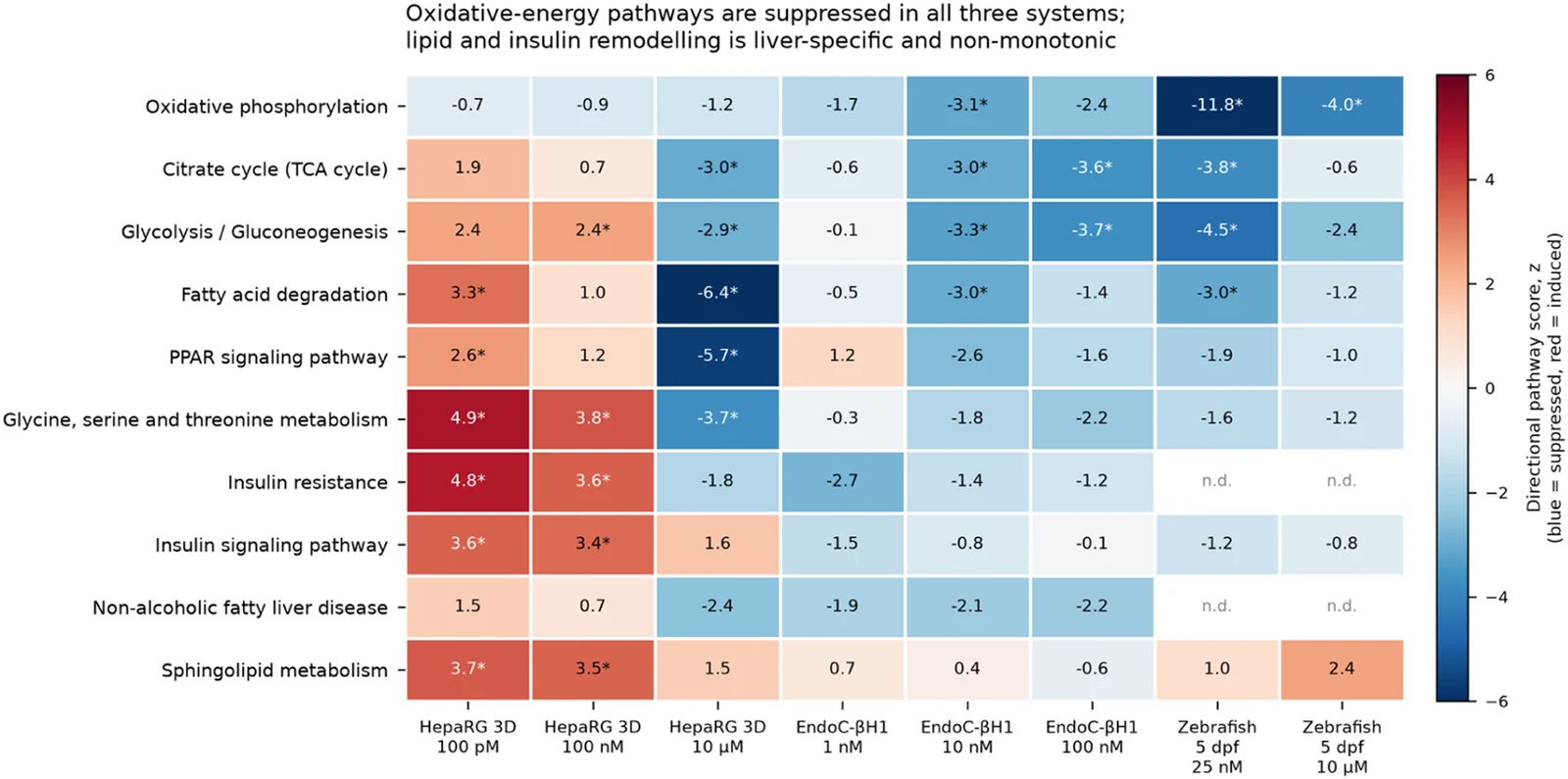
Conserved versus divergent pathway responses across the three experimental systems. Cell values are directional pathway scores (z), computed as the standardised mean moderated t-statistic of KEGG pathway member genes for each model-by-concentration contrast; blue denotes coordinated suppression and red coordinated induction, with the colour scale centred at z = 0 (no directional shift). Asterisks mark contrasts significant at BH-FDR < 0.05 in competitive gene-set testing (CAMERA, inter-gene correlation 0.01). n = 3 biological replicates per condition in the human cell systems and n = 4 in zebrafish. Oxidative-energy pathways (top three rows) are suppressed across all three systems, whereas PPAR signalling, fatty-acid metabolism and the serine/sphingolipid arms are liver-dominant and non-monotonic with concentration.

Three patterns emerge. First, suppression of oxidative-energy metabolism is the only module conserved across all three systems: oxidative phosphorylation is suppressed in every non-hepatic contrast and reaches FDR significance in zebrafish at both concentrations (z = −9.1 at 25 nM and −10.9 at 10 µM) and in EndoC-βH1 cells at 10 nM, while the TCA cycle and glycolysis/gluconeogenesis are suppressed and FDR-significant in HepaRG at 10 μM, in EndoC-βH1 at 10 and 100 nM and in zebrafish at both concentrations. The mitochondrial core of the network, KE3 through KE7, is therefore supported by concordant evidence across all three levels of biological organization.

Second, PPAR signalling and fatty-acid handling are essentially liver-specific and, importantly, non-monotonic. In HepaRG cells these pathways are induced at 100 pM (PPAR z = +3.5, fatty-acid metabolism z = +4.9, the latter FDR-significant) and strongly suppressed at 10 µM (z = −5.6 and −6.5, FDR = 6.2 × 10^−6^ and 7.3 × 10^−8^), consistent with receptor-mediated activation at environmentally relevant low concentrations giving way to a distinct, potentially adaptive or stress-related response at the highest concentration. The reversal of sign between the lowest and the highest concentration is the statistically supported non-monotonicity referred to in the Discussion. The same pathways show no coherent induction in zebrafish, which is expected given that the whole-larva homogenate dilutes a hepatic signal, and no induction in β-cells. The hepatic *in vitro* system captures the lipid-handling arm that the *in vivo* system does not resolve, so the two are not substitutable.

Third, the serine/S1P and insulin arms are detected principally in the hepatic and pancreatic *in vitro* systems. Glycine, serine and threonine metabolism is significantly induced in HepaRG at 100 pM and 100 nM (z = +5.3 and +4.1, both FDR-significant). Sphingolipid metabolism shows the same direction and concentration pattern (z = +2.8 at both 100 pM and 100 nM) but does not survive FDR correction, and the insulin signalling pathway shows a low-dose hepatic induction and a coherent zebrafish suppression that likewise falls just short of the corrected threshold; neither is treated here as a significant result. The evidence for the S1P key event is metabolomic rather than transcriptomic, resting on the directly measured S1P and ceramide species reported in Section 3.3.3, and the evidence for the β-cell insulin events rests on the reduced IRS1 expression and the reduced insulin secretion and content measured within OBERON. The corresponding zebrafish scores do not reach significance, which we attribute to tissue dilution and the shorter 2-day exposure rather than to absence of the mechanism.

Taken together, this comparison argues against replacing the *in vivo* arm with *in vitro* assays alone, and equally against relying on the *in vivo* arm alone. The *in vitro* systems are where the tissue-resolved upstream events, PPARγ-driven lipid remodelling, serine/S1P disruption and β-cell insulin signalling, are detectable at all; the zebrafish system is where the conserved oxidative-energy response is strongest and where whole-organism integration is available. For future testing of DEHP-adjacent structures, the practical implication is that a tiered design would be efficient: hepatic and β-cell *in vitro* assays to resolve the tissue-specific upstream key events, followed by a targeted *in vivo* confirmation restricted to the conserved mitochondrial module and the apical outcomes, rather than parallel full-omics profiling in all three systems.

## 4 Discussion

The development of the Adverse Outcome Pathway Network (AOPN) for DEHP exposure was based on an integrative systems biology approach combining multi-omics data (transcriptomics and metabolomics), computational modeling, and additional experimental evidence from *in vitro* and *in vivo* assays within the OBERON project, supported by mechanistic evidence from the literature. This integrative strategy enabled the reconstruction of a coherent sequence of molecular perturbations, illustrating how DEHP - associated changes may involve lipid metabolism, mitochondrial function, redox homeostasis, the serine/S1P axis, and insulin signaling. Together, these interconnected alterations illustrate the potential systemic implications of DEHP on metabolic homeostasis and provide a mechanistic basis for the proposed downstream outcomes of hepatic steatosis, progression of NAFLD, hepatic insulin resistance, and obesity.

By integrating high-throughput omics data, the study identified coordinated perturbations across key regulatory pathways involved in DEHP-induced metabolic dysfunction. Central molecular events, including PPARγ activation, FFA accumulation, TCA cycle overload, mitochondrial dysfunction, oxidative stress, serine depletion, S1P disruption, and impaired insulin signaling, were sequentially mapped and contextualized within the AOPN. This data-driven structure also highlighted reinforcing mechanisms such as the ATP–ROS feedback loop and the convergence of oxidative, inflammatory, and lipid-handling disruptions on hepatic insulin resistance. Computational modeling complemented these findings by placing the omics-derived perturbations into a mechanistic context, supporting dose-dependent shifts in acetyl-CoA and ATP levels. By linking experimentally observed perturbations to modelled dose–response relationships, this work provides dose–response descriptions that could be used to parameterise selected key event relationships in future work. These outputs are not interpreted as a validated quantitative basis for qAOP development. The palmitate 4PL fit (Figure 3) is constrained by three concentrations and one control, so its EC_50_ and Hill slope are descriptive summaries rather than identifiable parameters; the acetyl-CoA and ATP trajectories (Figure 4) are model predictions arising from a single fitted perturbation and were not independently validated against measured acetyl-CoA or ATP. Formal dose–response modelling was focused on palmitate because it provided the experimental lipid input used to parameterise the systems-biology model. Concentration-specific responses for the other key-event-associated metabolites are reported in the Results and Supplementary Material S1 and were interpreted as supporting evidence for the corresponding KEs rather than as independently fitted dose–response relationships. No formal identifiability, structural-uncertainty, parameter-sensitivity, or out-of-sample predictive analysis was performed. Establishing a genuine qAOP would require all four, together with response-response data at additional concentrations, and these requirements represent principal priorities for future work.

The analysis emphasized the interdependence of lipid metabolism, OXPHOS integrity, and insulin signaling, illustrating how discrete molecular disruptions associated with DEHP may propagate across biological systems. Similar systems-level approaches have proven valuable for refining predictive toxicology in other domains, such as developmental neurotoxicity (Spînu et al., 2021) and metal toxicity (Sahoo et al., 2024). Network-based studies have likewise linked phthalates, including DEHP, to metabolic and endocrine disturbances in humans and experimental models (Pogrmic-Majkic et al., 2022; Ravichandran et al., 2022; Zhang et al., 2022).

Traditional risk assessment approaches rely predominantly on apical endpoints, whereas the integration of multi-omics data and systems biology modeling enables a mechanistically anchored evaluation that captures early perturbations, pathway interactions, and system-level responses. By situating transcriptomic and metabolomic alterations within a structured network of KEs and KERs, the AOPN provides a structured basis for evaluating biological plausibility and supports inference across levels of organization. This enhanced mechanistic resolution advances the utility of AOP/AOPN frameworks within regulatory toxicology, complementing contemporary efforts to improve the mechanistic basis of chemical safety assessment (Villeneuve et al., 2018; Spinu et al., 2020). Importantly, the identification of early molecular events such as PPARγ activation, serine depletion, and sphingolipid imbalance highlights potential biomarkers of effect that could be leveraged in future screening, biomonitoring, or Integrated Approaches to Testing and Assessment (IATA) for metabolism-disrupting chemicals.

The regulatory value of the AOP network lies in translating chemical-specific multi-omics and dose–response findings into a structured set of directional KERs supported by explicit Weight-of-Evidence evaluations. The omics and dose-response analyses are chemical-specific: they describe the responses to DEHP in the investigated systems and concentration ranges. The AOP network is designed to organize these findings into a set of explicitly directional, individually falsifiable key event relationships, each supported by a documented weight-of-evidence evaluation. This structure makes the evidence, assumptions, and data gaps associated with each relationship transparent and allows individual KERs to be evaluated independently. Because AOPs are conceptually stressor-agnostic, the resulting mechanistic structure may also be relevant to other chemicals that perturb the same KEs, supporting its potential application in chemical grouping and read-across. The network additionally identifies measurable early events, including PPARγ activation, serine depletion and S1P imbalance, which may inform assay and biomarker prioritization and the development of Integrated Approaches to Testing and Assessment (IATA).

In its present form, the network is actionable for hypothesis generation, assay and biomarker prioritisation within an IATA, read-across justification and data gap identification, and it is suitable for submission to the OECD AOP Knowledge Base for community review, with the weight-of-evidence evaluations in Supplementary Material S1 presented in a format relevant to regulatory assessment. It is not yet suitable for quantitative point-of-departure derivation or for standalone risk assessment, which would require formal uncertainty analysis, parameter identifiability and sensitivity assessment, and validation against independent experimental data. Thus, the AOPN complements the DEHP-specific analyses by providing a transparent and potentially transferable framework for organizing their mechanistic implications. Moreover, because multiple environmental chemicals are known to converge on shared metabolic pathways, the AOPN presented here may serve as a template for evaluating cumulative or mixture effects when several agents trigger overlapping key events.

### 4.1 Measured, inferred and model-predicted endpoints

Because the network draws on several evidence types, we state explicitly which endpoints were measured in the present experiments, which were measured within OBERON and incorporated as supporting evidence, and which are inferred or model-predicted. Directly measured in this study were transcript abundance by microarray and metabolite abundance by UHPLC–HRMS, in all three systems. Measured within OBERON and used here as supporting evidence were triglyceride content and lipid-droplet accumulation in HepaRG 2D and 3D cultures, fatty-acid oxidation, glucose export, glucose consumption, glycogen content and Seahorse respirometry in hepatic models; insulin secretion, insulin content, electrical activity and viability in EndoC‐βH1 cells, together with steatogenic endpoints in zebrafish larvae. Not measured in any system reported here were acetyl-CoA, ATP, mitochondrial ROS, IRS1/IRS2 serine phosphorylation, PHGDH activity and metabolic flux; the corresponding key events (KE2, KE5, KE6 and KE9) are supported by transcriptional and model-based evidence and are flagged as inferred or model-predicted in Supplementary Material S1.

Taking the four disease-related endpoints in turn, steatosis (KE13) is the best supported, with directly measured triglyceride content and lipid-droplet accumulation in HepaRG cultures. Hepatic insulin resistance (KE14) was not measured by its defining criterion, since insulin-mediated suppression of hepatic glucose production was not assessed; it rests on transcript surrogates of the gluconeogenic programme (PCK1, G6PC) and an insulin-stimulated glucose-output assay is required to establish this key event experimentally. NAFLD (KE15) was not assessed histologically, biochemically or by imaging in any system, and the *in vitro* models cannot express its inflammatory and fibrotic components; it enters the network as a literature-supported apical outcome. Obesity (KE16) was likewise not measured, since no model used here can express whole-organism adiposity, and the essentiality call for KER14 rests on published pharmacological studies in rodents rather than on DEHP-specific data generated in this work.

A related distinction concerns what molecular abundance can demonstrate. Changes in the transcript levels of CS, FH, SDHC or ATP5MC3 constrain enzyme capacity but do not demonstrate altered flux, and metabolite pool sizes reflect the balance between production and consumption rather than either rate individually. The confirmatory experiments that would convert these interpretations into measurements are specific rather than generic: ^13^C flux analysis for the proposed TCA bottleneck, extracellular flux (Seahorse) analysis of basal, ATP-linked and maximal respiration for OXPHOS capacity, direct ROS and ATP quantification, and phospho-specific immunoblotting for IRS1/IRS2 serine phosphorylation.

Finally, the basis on which temporal and causal continuity is claimed across heterogeneous evidence should be stated plainly, because it cannot be asserted empirically within any single system used here. Exposure durations differ across the three models (14 days in HepaRG spheroids, 7 days in EndoC-βH1 cells, 2 days in zebrafish larvae), samples were taken at a single time point in each, and no attempt has been made to align them onto a common time axis. Continuity is therefore established at the level of biological plausibility and consistency, following OECD guidance: each KER is evaluated for temporal concordance using evidence internal to whichever system provides it, and agreement between systems is treated as evidence of conservation rather than as a shared time course. Concretely, the upstream lipid-handling and PPAR-related events are anchored in the hepatic system, the insulin-secretory events in the β-cell system, and the oxidative-energy events, the only module observed concordantly in all three systems (Figure 9), across all of them. Where an event is supported only by literature, this is stated in the weight-of-evidence evaluation and the essentiality call is downgraded accordingly. The network should thus be read as a mechanistic topology whose ordering is supported by plausibility and cross-system consistency, not as a validated temporal sequence within a single organism.

### 4.2 Species differences, *in vitro*–*in vivo* extrapolation and human exposure relevance

Because the network integrates evidence from two human cell systems and one non-mammalian *in vivo* model, the species and system differences between them constrain what can be extrapolated and are stated here explicitly. DEHP acts largely through its monoester metabolite MEHP, so the capacity of a system to hydrolyse the diester determines the response: differentiated 3D HepaRG spheroids retain carboxylesterase and cytochrome P450 activity and generate MEHP *in situ*, whereas EndoC-βH1 cells have limited xenobiotic-metabolising capacity, and zebrafish larvae carry a non-human esterase and receptor complement. Receptor pharmacology also differs: zebrafish possess duplicated PPAR paralogues (pparaa/pparab, ppardb, pparg) whose ligand-binding domains are not fully orthologous to human PPARγ, and MEHP potency at the zebrafish receptors is not established. The absence of a coherent PPAR signal in the zebrafish contrasts reported in Section 3.4 is therefore consistent with three non-exclusive explanations that the present design cannot separate: receptor divergence, dilution of a hepatic signal in whole-larva homogenate, and the shorter 2-day exposure relative to 14 days in HepaRG. We have accordingly restricted cross-species inference to the oxidative-energy module, which is the only module concordant across all three systems and whose constituent enzymes are highly conserved, and we treat the liver-specific and β-cell-specific arms as human-cell-derived evidence that the zebrafish data neither corroborate nor contradict.

Quantitative in vitro–in vivo extrapolation was not performed and the present data do not support it. The nominal concentrations applied *in vitro* are not free concentrations: DEHP is highly lipophilic (log Kow ≈ 7.6) and binds extensively to plastic labware, serum protein and the collagen matrix of the spheroid culture, so the freely dissolved fraction driving receptor occupancy is expected to be substantially lower than nominal and was not measured. No exposure medium or intracellular concentrations were determined in any system, and no PBK model linking external dose to target-tissue concentration was applied to the datasets reported here. Consequently, the concentration axis of Figure 9 should be read as a nominal-dose axis within each system rather than as a common internal-dose axis across systems, and the non-monotonic hepatic PPAR response is established with respect to nominal concentration only. Measurement of free and cell-associated MEHP alongside a PBK-based reverse-dosimetry step is required before the concentration-response relationships reported here can be placed on a human-equivalent dose scale, and we identify this as a prerequisite for any future quantitative use of the network.

The relevance of the tested range to human exposure should likewise be stated with care. Human biomonitoring indicates that general-population internal exposure to DEHP metabolites is low: serum concentrations of MEHP reported in population surveys are typically in the low ng mL^-1^ range, corresponding to roughly single-digit to low tens of nanomolar, with higher burdens in occupationally exposed groups and in patients exposed through DEHP-containing medical devices such as during haemodialysis, transfusion or extracorporeal circulation (Katsikantami et al., 2016; Wang et al., 2019). On this basis the 1–100 nM exposures in EndoC-βH1 cells and the 100 pM and 100 nM exposures in HepaRG spheroids fall within or close to the range of internal concentrations plausibly encountered by the general and clinically exposed populations, and dose–internal concentration modelling within OBERON was used to support the selection of these levels. The 10 µM exposures used in HepaRG spheroids and zebrafish larvae exceed plausible human internal concentrations by two to three orders of magnitude and were included as a mechanistic upper anchor and positive control for lipid accumulation rather than as a human-relevant scenario. This distinction matters for interpretation, because the pathway responses at 10 µM in HepaRG are directionally opposite to those at 100 pM (Figure 9): the low-concentration induction of PPAR signalling and fatty-acid degradation is the response occurring in the human-relevant part of the range, whereas the high-concentration suppression is likely to reflect a distinct, possibly stress-related or cytostatic, mode of action. Extrapolation of the network to human risk should therefore rest on the low-nanomolar and sub-nanomolar findings, and the high-concentration data should not be used to support a monotonic read-across to human exposure levels.

### 4.3 Limitations and future directions

While our study presents a comprehensive AOPN for DEHP-induced metabolic dysregulation, several limitations should be acknowledged. First, although our systems biology model generated dose-dependent mechanistic predictions, data gaps in kinetic parameters remain a challenge. Further refinement through experimental kinetic measurement is needed to improve predictive accuracy, and it is now possible to say specifically which parameters would deliver the greatest improvement. Priority kinetic measurements include:(i) carnitine palmitoyltransferase 1 (CPT1A/CPT1B) Vmax and Km for palmitoyl-CoA, together with the malonyl-CoA inhibition constant, since CPT1 sets the β-oxidation entry rate that drives the entire acetyl-CoA branch of the network and is currently the least constrained parameter in the model; (ii) citrate synthase and succinate dehydrogenase Vmax under the exposure conditions, which determine whether the predicted TCA bottleneck actually forms; (iii) ATP synthase turnover and the proton-leak conductance including the UCP3 contribution, which jointly govern the predicted ATP depletion and its coupling to ROS; (iv) serine palmitoyltransferase and phosphoglycerate dehydrogenase kinetics, which control partitioning of palmitoyl-CoA between complete oxidation and ceramide/S1P synthesis and therefore the branch point that distinguishes our proposed serine/S1P mechanism from conventional lipotoxicity; and (v) the concentration-dependence of MEHP binding at PPARγ, which would convert the empirical DEHP scaling factor into a receptor-occupancy term. Extracellular flux (Seahorse) measurements of basal, ATP-linked and maximal respiration across the exposure range, and ^13^C-palmitate tracing of TCA entry, would constrain groups (i) to (iii) simultaneously and are the most efficient single experiment we can identify (Price et al., 2020; Jin et al., 2022). Nevertheless, the dose–response relationships generated for key intermediates such as palmitate, acetyl-CoA, and ATP provide a foundation for future parameterization of quantitative KERs within a qAOP framework.

A further limitation, made explicit here, is statistical power. With three biological replicates per condition in the human cell systems and four in zebrafish, the individual omics contrasts are underpowered for gene-level FDR control, and as reported in Sections 2.2.3, 3.1.1 almost no single transcript survives Benjamini-Hochberg correction. This is a real constraint on what the present data can establish: it means the network identifies which processes are perturbed with reasonable confidence, but cannot rank the contribution of individual genes within those processes. For this reason we describe the framework as hypothesis-generating and evidence-integrating rather than quantitatively validated. Replication at n ≥ 6 per condition, which would bring gene-level FDR within reach for effects of the observed magnitude, is the single most valuable addition to the experimental design for follow-up work.

Additionally, this study primarily focused on hepatic and pancreatic cellular models due to their central roles in metabolic regulation, capturing hepatic insulin resistance and pancreatic β-cell dysfunction. However, DEHP-induced metabolic dysfunction also involves adipose tissue, skeletal muscle, and neuroendocrine regulation. Future investigations should extend this approach to other metabolically active tissues, such as adipose and muscle, to construct a more holistic representation of systemic metabolic perturbations. Moreover, the relianceon established *in vitro* models does not fully recapitulate tissue heterogeneity and multicellular interactions present *in vivo*, which may influence the dynamics of certain key events.

Another limitation relates to differences between experimental exposure scenarios and human exposure patterns. While the applied *in vitro* and *in vivo* models capture mechanistic responses to DEHP, human exposure is typically chronic and involves complex chemical mixtures. Although dose–internal concentration modeling within OBERON was used to support biological relevance of the tested exposure ranges, uncertainty remains in extrapolating acute experimental responses to long-term human exposure scenarios. Integrating human biomonitoring data, real-life exposure profiles, and mixture effects would enhance the translational relevance of the AOPN. As set out in Section 4.2, free and cell-associated MEHP concentrations were not measured in any system and no PBK-based reverse dosimetry was applied to the present datasets, so the concentration-response relationships reported here are expressed on a nominal-dose basis and are not yet on a human-equivalent dose scale. Finally, emerging computational approaches, including deep learning, AI-assisted evidence extraction, and automated network inference, offer opportunities to refine AOPN construction and increase reproducibility. Recent applications of machine learning in AOP development, including automated evidence curation for cholestasis (van Ertvelde et al., 2023) and transcriptome-derived vascular AOP networks (Stanic et al., 2024) demonstrate the potential of these tools for scaling and validating AOPNs. Continued integration of probabilistic modeling and uncertainty quantification methods (Perkins et al., 2019; Zgheib et al., 2019; Spinu et al., 2020; Jin et al., 2022) will be essential for advancing qAOPs toward regulatory acceptance.

## 5 Conclusion

This work presents a mechanistically anchored Adverse Outcome Pathway Network (AOPN) for DEHP-induced metabolic disruption, developed through the integration of multi-omics data and systems biology modeling. The combined evidence supports a coherent sequence of molecular events proposed to link early lipid remodeling, mitochondrial dysfunction, oxidative stress, and serine/S1P pathway disruption to impaired insulin signaling, steatosis, hepatic insulin resistance, NAFLD, and obesity. By explicitly defining interconnected KEs and KERs and providing dose–response descriptions for selected relationships, this AOPN provides a starting point from which quantitative AOP (qAOP) development could proceed, without itself constituting a quantitatively validated qAOP. It should be read as a hypothesis-generating, evidence-integrating framework for understanding DEHP-induced metabolic disruption and supporting future risk assessment. Formal identifiability, uncertainty and sensitivity analyses, additional kinetic data and broader tissue-level validation are required before predictive use, this integrative approach demonstrates the value of systems biology in advancing mechanistically informed chemical safety assessment. Overall, the proposed AOPN offers a systems toxicology framework for evaluating the metabolic risks of DEHP and related environmental contaminants.

## Data Availability

The datasets generated and analyzed during the current study are publicly available in the Zenodo repository at https://doi.org/10.5281/zenodo.20376941 (concept DOI https://doi.org/10.5281/zenodo.20376940). The R code and full output of the FDR-controlled sensitivity reanalysis and the cross-system gene-set comparison performed in response to peer review, including per-gene refitted statistics for all eight contrasts and the complete CAMERA results, are provided in Supplementary Material 3 and deposited in the same repository.
