# Supplementary material for: Mapping DEHP-induced metabolic dysregulation through multi-omics and systems biology: an adverse outcome pathway network approach
Source: Front Toxicol. 2026 Sep 30;8:1930835. doi: 10.3389/ftox.2026.1930835 (PMC13661021; doi:10.3389/ftox.2026.1930835)
Supplement: Supplementary file 1 [file Supplementaryfile1.docx]

# Supplementary Material 1

## KEs, KERs and Supporting Evidence

This Supplementary Material summarizes all molecular and cellular Key Events (KEs) and Key Event Relationships (KERs) within the Adverse Outcome Network (AON) describing metabolic disruption induced by chronic DEHP exposure. Each KE section presents the supporting mechanistic and empirical evidence derived from multi-omics analyses, in vitro and in vivo experiments, and literature data.

For each KER, a Weight-of-Evidence (WoE) evaluation was performed following OECD guidance (OECD, 2018), addressing five criteria: biological plausibility, consistency, dose–response, temporal concordance, and essentiality of the upstream KE. The WoE evaluation integrates experimental findings, outputs from systems biology modeling performed within this study, and literature evidence to assess the overall strength of causal linkages within the network.

Detailed experimental protocols, transcriptomic and metabolomic workflows, supporting these findings are described in Supplementary Material 2.

MIE: PPARG Activation

Exposure to DEHP led to significant upregulation of PPARγ at low concentrations (log_2_FC0.1nM_LU_ =0.589, log2FC0.1nM_GU_= 0.705) in HepaRG liver cells, as identified by both Limma and GeneSpring bioinformatics methods. PPARγ is a nuclear receptor that regulates lipid metabolism, primarily promoting lipid uptake, storage, and adipogenic signaling in hepatocytes. Its activation is consistent with increased lipid droplet formation and lipid metabolic remodeling, a common response to environmental toxicants affecting metabolic regulation (Ding et al., 2021; Haiqin Fang et al., 2019; Hsu et al., 2020; Zeng et al., 2018; W. Zhang et al., 2017). The interaction between DEHP, its metabolite MEHP, and the PPAR family is extensively documented in human hepatic cells (Hurst & Waxman, 2003; Kocbach Bølling et al., 2012; Useini, Engelberger, Künze, & Sträter, 2023; Venkata et al., 2006).

Additionally, the RXRB gene, part of the NR2B nuclear receptor family, showed significant up-regulation at two DEHP concentrations in HepaRG cells. RXRB interacts with several nuclear receptors, including PPARs, LXRs, and VDRs (Chai & Chen, 2022; Dawson & Xia, 2012; Lefebvre, Benomar, & Staels, 2010). Given that these receptors were not identified in this study's HepaRG cells, the up-regulation is likely due to PPAR-gamma disruption. Furthermore, CASP3 (Caspase 3) was up-regulated at the same low DEHP concentrations as PPAR-gamma, an association supported by multiple studies (Rusyn, Peters, & Cunningham, 2006; Ryu et al., 2007). Therefore, experimental data and literature review suggest that DEHP exposure may induce PPAR-gamma binding and activation, although the exact mechanism remains unclear (Useini et al., 2023).

While many studies link PPAR family disruption to DEHP exposure in zebrafish, the data obtained for this study pertain to a 5-day exposure duration, limiting the evidence's robustness. Most literature suggests that a minimum of 10 days is needed to observe PPAR family disruption due to DEHP exposure (Buerger et al., 2019; Maradonna et al., 2013; C. G. Park, Sung, Ryu, & Kim, 2020; Uren-Webster, Lewis, Filby, Paull, & Santos, 2010).

Furthermore, PPARGC1A was significantly downregulated in HepaRG cells (logFC100pM = -1.03, logFC 100nM = -0.67). PPARGC1A plays a key role in mitochondrial biogenesis and fatty acid oxidation, facilitating the metabolic shift towards lipid breakdown and energy production. Its suppression shifts hepatic metabolism toward lipid accumulation rather than oxidation, reinforcing a metabolic imbalance where lipid uptake and storage are enhanced, while β-oxidation is delayed (Qian et al., 2024). This downregulation was accompanied by disruptions in phospholipid homeostasis, as evidenced by reductions in phosphatidylcholine (PC) and phosphatidylethanolamine (PE) levels, which are essential for membrane integrity and lipid trafficking. The combined PPARγ activation and PPARGC1A downregulation create a condition in which fatty acid influx (i.e. FFA pool/turnover) outpaces their utilization, predisposing hepatocytes to lipotoxic stress and metabolic overload.

KER1: PPARG Activation Leading to Free FA Accumulation

PPARγ activation in hepatocytes modulates lipid metabolism by promoting lipid uptake and triglyceride storage. However, in the context of PPARGC1A downregulation, the expected induction of fatty acid oxidation is compromised, leading to an imbalance between lipid accumulation and utilization.

Transcriptomic data revealed that CIDEB and CIDEC (FSP27), which are crucial regulators of lipid droplet stability, were significantly downregulated (log2FC10µM = -0.71 and -0.82 in HepaRG cells, respectively). These genes encode proteins which are essential for maintaining lipid droplet integrity and their downregulation enhances lipolysis, leading to increased release of FFAs into the cytoplasm (Slayton et al., 2019; Yu et al., 2003). Additionally, perilipins (PLIN1 and PLIN2), which help stabilize triglyceride (TAG) storage within lipid droplets, were also significantly downregulated(log2FC10µM = -0.68 and -1.15, respectively), potentially further destabilizing lipid droplets and facilitating FFA mobilization (Hansen, De Maré, Jones, Göransson, & Lindkvist-Petersson, 2017; Martinez-Botas et al., 2000; Souza et al., 2002; J. T. Tansey et al., 2001; John T. Tansey et al., 2003). Collectively, these transcriptional changes indicate a shift toward altered lipid handling that may increase intracellular FFA availability.

Additionally, increased expression of key lipolytic enzymes, such as adipose triglyceride lipase (ATGL, PNPLA2) (log2FC100nM = 0.61, log2FC10µM = 0.39) and hormone-sensitive lipase (HSL, LIPE) (log2FC10µM = 1.02), indicated an overall shift toward lipolysis-driven FFA release(Hao, Cheng, Xia, & Ma, 2012). Monoglyceride lipase (MGLL), responsible for hydrolyzing monoacylglycerols (MAGs) into FFAs and glycerol, was downregulated (log2FC10µM = -1.47), suggesting an incomplete breakdown of triglycerides and an imbalance in lipid turnover.

This stage represents an early accumulation of cytoplasmic FFAs, which primarily exceed mitochondrial oxidative capacity, reinforcing the delay in β-oxidation seen in early temporal stages. This mismatch between lipid influx and oxidation initiates a cascade of metabolic disruptions, driving hepatocytes toward lipotoxic stress and metabolic inflexibility.

**Weight-of-Evidence (KER1)**

- **Evidence and Plausibility:** Activation of PPARγ by DEHP or its active metabolite MEHP is biologically plausible and well supported. PPARγ is a nuclear receptor controlling adipogenic and lipogenic programs; its activation promotes lipid uptake and storage while influencing genes regulating lipid droplet dynamics. The observed FFA accumulation is consistent with PPARγ-driven modulation of lipid metabolism in hepatocytes and adipocytes (Feige et al., 2007; Hurst & Waxman, 2003; W. Zhang et al., 2017)
- **Dose–Response and Temporal Concordance:** Multiple studies, including the present work, show dose-dependent activation of PPARγ at low nanomolar concentrations (0.1–100 nM) with a plateau at higher micromolar doses, suggesting a threshold-type response consistent with receptor-mediated mechanisms. PPARγ activation occurs early following DEHP exposure and precedes measurable lipid accumulation. Longer in vivo exposures (≥10 days) are typically required for downstream lipid storage effects, supporting the temporal sequence of this KER.
- **Essentiality of the Upstream KE:** Antagonism or genetic silencing of PPARγ diminishes lipid accumulation and reduces triglyceride storage, confirming its essential role in initiating FFA buildup (Feige et al., 2007).

KE1: Free Fatty Acid (FA) Accumulation

The accumulation of free fatty acids (FFAs) in hepatocytes following DEHP exposure is primarily driven by increased TG lipolysis and impaired mitochondrial fatty acid oxidation. Hepatic lipid droplets, which normally serve as dynamic energy reservoirs, become destabilized due to the downregulation of CIDEB, CIDEC (FSP27), PLIN1, and PLIN2, leading to excessive lipid breakdown(Alves-Bezerra & Cohen, 2017; Walther & Farese, 2012; A. Yang & Mottillo, 2020). This was further supported by decreased phospholipid levels, particularly PC and PE, suggesting altered membrane lipid composition and disrupted lipid trafficking.

As a direct consequence of excess FFAs, the metabolic shift toward fatty acid oxidation becomes increasingly necessary. However, at this stage, β-oxidation is still limited, as indicated by the lack of significant upregulation of fatty acid oxidation enzymes. Instead, the accumulation of FFA-CoA intermediates creates metabolic bottlenecks that increase lipotoxic stress. The metabolomic profile of HepaRG cells exposed to DEHP revealed significant reductions in fatty acid degradation metabolites, including glutarate (log2FC100pM = -2.67, log2FC100nM = -2.67, log2FC10µM = -3.22) and palmitic acid (log2FC100pM = -0.52, log2FC100nM = -1.32, log2FC10µM = -1.98), suggesting impaired lipid breakdown pathways.

The reduction in palmitic acid is consistent with its diversion into alternative lipid pathways under lipotoxic stress. Palmitoyl-CoA derived from palmitic acid is the initiating substrate for ceramide biosynthesis, and increased flux through this pathway has been linked to steatosis and insulin resistance (Hajduch, Lachkar, Ferré, & Foufelle, 2021; Lipke, Kubis-Kubiak, & Piwowar, 2022; Summers, 2006). Consistent with diversion of saturated fatty acids into sphingolipids, several long-chain ceramide species were markedly increased in HepaRG cells following DEHP exposure, including Cer(d18:0/16:0) (log2FC100pM = 2.64, log2FC100nM =3.39, log2FC10µM = 4.43) and Cer(d18:0/18:0) (log2FC100pM = 4.11, log2FC100nM =4.68, log2FC10µM = 5.23) and, while the canonical C16 sphingosine-1-phosphate species (S1P d16:1) was strongly decreased, the atypical species S1P(d19:1-P) was increased. This pattern supports a lipotoxic rerouting of palmitoyl-CoA and other FFAs toward ceramide and non-canonical S1P species, rather than complete fatty-acid catabolism (Ashrap et al., 2021, 2022).

In parallel, incomplete fatty acid oxidation promotes the accumulation of long-chain acylcarnitines, including palmitoylcarnitine, which are established indicators of mitochondrial overload (Bjørndal et al., 2018; Sampey et al., 2012; Wajner & Amaral, 2016; X. Zhang, Zhang, Chen, Han, & Ji, 2014). These diversion routes explain why palmitic acid levels decrease even as the overall free fatty acid pool expands, thereby linking lipid accumulation with early mitochondrial dysfunction (Moliterni et al., 2024; Yoon, Shaw, Haigis, & Greka, 2021).

Supporting in vivo studies in rodents show elevated circulating FFAs and altered hepatic expression of lipolytic regulators after DEHP exposure (DONG et al., 2021; E. J. Lee, Hong, & Yang, 2024; Yuexia Zhang et al., 2020), while shorter exposures in zebrafish produce milder effects, consistent with time-dependent accumulation dynamics.

At this point, fatty acid oxidation has not yet fully compensated for the increased lipid influx, setting the stage for later metabolic shifts toward mitochondrial overload, oxidative stress, and dysregulated TCA cycle activity.

KER2: FFA accumulation Leads to an Increase of Acetyl-CoA

DEHP-induced FFA accumulation in hepatic cells triggers a metabolic shift toward β-oxidation, leading to increased acetyl-CoA production. Initially, lipolysis-driven FFA accumulation exceeds mitochondrial oxidation capacity, resulting in lipid accumulation. However, as metabolic compensation mechanisms activate, β-oxidation is upregulated, evidenced by increased CPT1B expression, which facilitates fatty acid transport into mitochondria (Y. H. Chen et al., 2020; Gu et al., 2022; Ide & Origuchi, 2020; Regnault et al., 2018).This response initially functions to mitigate FFA overload, it ultimately fails to restore metabolic balance. In this context, acetyl-CoA accumulation arises from substrate overload driven by excess FFA availability rather than from impaired ATP synthesis. When acetyl-CoA production exceeds the processing capacity of the tricarboxylic acid (TCA) cycle and downstream oxidative phosphorylation (Arnold & Finley, 2023; Pietrocola, Galluzzi, Bravo-San Pedro, Madeo, & Kroemer, 2015), redox imbalance (elevated NADH/NAD⁺ ratio), feedback inhibition of TCA dehydrogenases, and mitochondrial protein hyperacetylation ensue. These processes compromise mitochondrial efficiency and precede the downstream decline in ATP production.

**Weight-of-Evidence (KER2)**

- **Evidence and Plausibility:** Elevated acetyl-CoA levels have been consistently observed after phthalate exposure in liver cells and animal models, reflecting enhanced β-oxidation activity and metabolic stress(Hala et al., 2021). This outcome is biologically plausible given the central role of acetyl-CoA as a metabolic intermediate linking lipid catabolism to energy homeostasis and redox regulation. DEHP exposure has been shown to overcompensate for CoA trapping, leading to increased levels of free CoA and various CoA esters, indicative of altered oxidative metabolism (Hala et al., 2021).
- **Dose–Response and Temporal Concordance:** A correlation between intracellular FFA levels and acetyl-CoA production has been demonstrated in HepaRG and rodent hepatocytes. Higher DEHP concentrations result in proportionally greater acetyl-CoA responses, consistent with a substrate-driven process. Temporally, acetyl-CoA accumulation follows FFA buildup, confirming the expected sequence of events and supporting the causal directionality of this relationship.
- **Essentiality:** The generation of excess acetyl-CoA represents a pivotal metabolic juncture because it feeds into the tricarboxylic acid (TCA) cycle (Arnold & Finley, 2023; Pietrocola et al., 2015) and alternative pathways such as ketogenesis (Koves et al., 2023; Muyyarikkandy et al., 2020) and lipid synthesis (Kawano & Cohen, 2013; Rui, 2014; Shi & Tu, 2015) . Blocking β-oxidation (e.g., via CPT1 inhibition) prevents acetyl-CoA accumulation (Jung et al., 2024; van Eunen et al., 2013); in such cases, unoxidized FFAs would likely be re-esterified into triglycerides, potentially worsening steatosis (Kawano & Cohen, 2013; Rui, 2014). Conversely, when FFA influx is limited, acetyl-CoA overload and its consequences (TCA cycle stress, ROS generation) may not occur. Given these compensatory possibilities, acetyl-CoA increase shows moderate essentiality; it is a key step in propagating energy stress and signaling cascades (e.g., activation of protein kinase C via altered NADH/NAD⁺ ratio), yet alternate routes such as peroxisomal oxidation or lipid storage could partially buffer its impact. Nonetheless, experimental inhibition of β-oxidation enzymes such as CPT1A and HADHA prevents acetyl-CoA accumulation, and pharmacological stimulation enhances it, confirming the functional dependence of this KER on upstream lipid availability.

KΕ2: Increase of Acetyl-CoA

The increase of Acetyl-CoA, driven by the β-oxidation of fatty acids in the mitochondria, activates fatty acids via esterification with CoA, leading to their degradation through the TCA cycle for energy production and maintaining cellular energy balance (Schulz, 2013). The increased levels of Acetyl-CoA are a result of enhanced fatty acid oxidation within the mitochondria. This involves the activation of acyl-CoA synthetases, the L-carnitine system, and specific enzymes that facilitate the breakdown of fatty acids into Acetyl-CoA and shorter fatty acyl-CoA esters (Adeva-Andany, Carneiro-Freire, Seco-Filgueira, Fernández-Fernández, & Mouriño-Bayolo, 2019; Schulz, 2013). In the present study, acetyl-CoA was not measured experimentally. The systems biology model predicted an increase in hepatic acetyl-CoA concentration following reparameterisation using the experimental HepaRG metabolomics data. This model prediction was interpreted together with the broader pathway-level evidence and should not be considered a measured increase in acetyl-CoA.

KER3: Increase of Acetyl-CoA leads to Dysregulation of TCA cycle

Building on the acetyl-CoA increase described above, metabolomic profiling revealed reductions in phosphatidylcholine (PC) and phosphatidylethanolamine (PE), indicating a shift away from phospholipid synthesis toward oxidation. This shift generates excess acetyl-CoA, fueling the TCA cycle and ketogenesis (Adeva-Andany et al., 2019; Schulz, 2013). Elevated acetyl-CoA overloads mitochondrial capacity, creating a bottleneck in the TCA cycle and altering the NADH/NAD⁺ ratio(Arnold & Finley, 2023; Pietrocola et al., 2015). This triggers oxidative stress and activation of PKC (log₂FC₁₀ µM = 0.60) and NOX (log₂FC₁₀₀ pM = 0.99; log₂FC₁₀₀ nM = 1.07; log₂FC₁₀ µM = 0.80) in HepaRG cells, further exacerbating mitochondrial dysfunction (Begriche, Massart, Robin, Bonnet, & Fromenty, 2013; Galili et al., 2007).

**Weight-of-Evidence (KER3)**

- **Evidence and Plausibility**: When acetyl-CoA production exceeds TCA capacity, dehydrogenases are feedback-inhibited (Arnold & Finley, 2023) and mitochondrial protein hyperacetylation increases(Yu Zhang et al., 2021). Comparable mechanisms occur in NAFLD, where fatty-acid–derived acetyl-CoA is diverted to ketogenesis or accumulates as intermediates (Kawano & Cohen, 2013; Koves et al., 2023; Muyyarikkandy et al., 2020). Down-regulation of multiple TCA enzymes (CS, IDH2, FH, SDHC) and compensatory responses in HepaRG are consistent with this mechanism. Elevated acetyl-CoA levels directly influence histone acetylation, linking cellular metabolism to gene expression and potentially contributing to dysregulation (Galdieri & Vancura, 2012; Wellen et al., 2009)
- **Dose–Response and Temporal Concordance:** The degree of TCA-cycle disruption increases with acetyl-CoA levels (Arnold & Finley, 2023; Pietrocola et al., 2015). Zebrafish exposed to 10 µM DEHP exhibited stronger enzyme down-regulation and metabolite shifts than those at 25 nM. In HepaRG cells, pathway perturbation became evident only at higher nanomolar concentrations, suggesting a threshold effect. Temporally, TCA dysregulation follows acetyl-CoA accumulation and coincides with early mitochondrial stress observed within five days of exposure. The accumulation of acetyl-CoA and subsequent impact on the TCA cycle in response to toxicants highlight a clear temporal sequence (Koves et al., 2023)
- **Essentiality:** A functional TCA cycle is essential for maintaining ATP generation and redox balance. Preventing acetyl-CoA accumulation by diverting FFAs to storage or increasing its utilization reduces TCA impairment, whereas enhanced mitochondrial acetyl-CoA delivery exacerbates cycle stress, as shown in high-fat-diet models (Begriche et al., 2013). Essentiality is therefore judged moderate: TCA-cycle dysregulation is a critical driver of energy failure and oxidative stress, yet some downstream ROS formation may still occur through parallel mechanisms even if partial TCA activity is preserved.

KE3: Dysregulation of TCA Cycle

The multi-omics analysis performed on 5-day post-fertilization (dpf) zebrafish larvae exposed to DEHP (25 nM and 10 µM) revealed that the tricarboxylic acid (TCA) cycle was significantly disrupted (p = 0.0012; impact factor = 2.384), as illustrated in Figure S1.1.

Several key enzymes involved in the TCA cycle exhibited decreased expression after DEHP exposure, including isocitrate dehydrogenase (IDH2) (–2.05 at 25 nM; –2.24 at 10 µM), fumarate hydratase (FH), aconitase 2 (ACO2), dihydrolipoamide dehydrogenase (DLDH), dihydrolipoamide S-succinyltransferase (DLST), succinate-CoA ligase alpha subunit (SUCLG1), malate dehydrogenase 1Aa (MDH1AA), succinate dehydrogenase complex subunit C (SDHC), and citrate synthase (CS). The strongest decrease was observed for IDH2 (–2.05 at 25 nM and –2.24 at 10 µM).

Conversely, phosphoenolpyruvate carboxykinase 1 (PCK1) showed moderate up-regulation (+0.86 at 25 nM; +0.69 at 10 nM), suggesting redirection of TCA intermediates toward gluconeogenesis. Citric acid itself increased in abundance (+1.874 and +1.726 log₂FC at 25 nM and 10 µM, respectively), aligning with elevated acetyl-CoA levels observed in systems-biology modeling.

Further analysis in 3D HepaRG liver cells exposed to DEHP (0.1 and 100 nM) showed up-regulation of isocitrate dehydrogenase (NADP⁺) 2 (IDH2) (+1.03 and +1.11 log₂FC, respectively) and phosphoenolpyruvate carboxykinase 2 (PCK2) (+0.81 log₂FC at 100 nM). Despite these changes, the overall pathway perturbation in HepaRG cells was not statistically significant (p = 0.5942).

Overall, DEHP exposure induced TCA-cycle enzyme expression dysregulationin zebrafish and partial compensatory activation in HepaRG cells, consistent with dose- and model-dependent mitochondrial metabolic imbalance. The detailed pathway analysis and visualization of TCA-cycle dysregulation are presented in Figure S1.1.

*Figure S1.1. The plot on the left shows the summary of multi-omics pathway analysis using the differential expressed genes, and metabolites in zebrafish larvae of 5 dpf exposed to DEHP (25Nm, 10μΜ), using the MetaboAnalystR package. Pathways are plotted according to significance (y-axis) and pathway impact value (x-axis). Larger circles represent greater pathway enrichment and darker colours represent more significance. Most contributing pathways are in the top right corner. Glycolysis/Gluconeogenesis: p = 0.0035; impact = 1.208. Citrate cycle (TCA): p = 0.0012; impact = 2.384.*

*The plot on the right shows the expression of genes in the TCA cycle from KEGG pathway enrichment analysis. Down regulated genes or metabolites are noted with blue, while upregulated with red colour. CS; citrate synthase, DLDH; dihydrolipoamide dehydrogenase, DLST; dihydrolipoamide S-succinyltransferase, FH; fumarate hydratase, IDH2; isocitrate dehydrogenase (NADP(+)) 2, ACO2; aconitase 2, PCK1; phosphoenolpyruvate carboxykinase 1, and SDHC; Succinate-CoA Ligase Alpha Subunit, SUCLG1; malate dehydrogenase 1Aa, NAD (soluble), MDH1AA.*

*Right: KEGG TCA map with expression changes (blue = down; red = up). Abbreviations: CS, DLDH, DLST, FH, IDH2, ACO2, PCK1, SDHC, SUCLG1, MDH1AA.*

KER4: Dysregulation of TCA cycle leading to disruption of OXPHOS

Impairment of the TCA cycle inevitably affects the electron transport chain (ETC) and ATP synthesis, since the TCA cycle provides NADH/FADH₂ to fuel OXPHOS.
In DEHP exposure, we observe clear evidence of mitochondrial dysfunction manifesting as OXPHOS disruption across all models. Zebrafish larvae exposed to DEHP showed decreased expression of multiple ETC components. These changes coincide with the TCA-enzyme decreases noted above (KE3), indicating a consistent pattern of mitochondrial impairment. Mechanistically, when the TCA cycle is inefficient, electron donors (NADH) may become limited or imbalanced, and mitochondrial high-energy electron transfer can stall (Schulz, 2013). Additionally, excess acetyl-CoA and its by-products can damage mitochondria (e.g., via protein hyper-acetylation or over-activation of PKC), which interferes with ETC activity.

Our HepaRG data showed up-regulation of PKC and NADPH oxidase (KER 3) in concert with these events, further contributing to mitochondrial stress.

The plausibility of this KER is extremely high; it is well-established that mitochondrial overload and metabolic stress cause ETC uncoupling and reduced ATP production (K. Lee et al., 2018).

**Weight-of-Evidence (KER4)**

- **Evidence & Plausibility:** Strong across models (zebrafish, pancreatic β-cells, HepaRG). Impairment of the TCA cycle inevitably affects the electron transport chain and ATP synthesis, since the TCA cycle provides NADH/FADH₂ to fuel OXPHOS (Begriche et al., 2013). In DEHP exposure, we observe clear evidence of mitochondrial dysfunction manifesting as OXPHOS disruption. Zebrafish larvae exposed to DEHP showed decreased expression of multiple ETC components. These changes coincide with the TCA-enzyme decreases noted above, indicating a consistent pattern of mitochondrial impairment. Mechanistically, when the TCA cycle is inefficient, electron donors may become limited or imbalanced, and mitochondrial high-energy electron transfer can stall(San-Millán, 2023).
- **Dose–Response & Temporal Concordance:** OXPHOS disruption shows threshold behavior — clear at ≥10 µM DEHP; emerges after several days of exposure. These changes are consistent with DEHP-induced mitochondrial impairment affecting ETC activity (Y. Huang et al., 2019; E. J. Lee et al., 2024)
- **Essentiality:** Integrity of OXPHOS is crucial for cell viability. UCP3 up-regulation is adaptive but insufficient to prevent ATP loss and oxidative stress (R. Z. Zhao, Jiang, Zhang, & Yu, 2019). Hence this KER is highly essential for progression toward adverse outcomes. Mitochondrial dysfunction is a key hallmark of environmental insults and leads to critical adverse effects (Reddam, McLarnan, & Kupsco, 2022).

KE4: Disruption of OXPHOS

Disruption of oxidative phosphorylation (OXPHOS) represents a proposed downstream mitochondrial consequence of TCA-cycle dysregulation. Within the present omics datasets, OXPHOS disruption was inferred from coordinated transcriptional changes in pancreatic β-cells and zebrafish larvae rather than measured directly as respiratory flux.. In zebrafish larvae exposed to DEHP (25 nM and 10 µM, 5 dpf), the coordinated down-regulation of TCA enzymes (KE3) suggests impaired electron-donor supply to the respiratory chain.

Disruption is also highlighted by the downregulation of the Complex I subunit NDUFA6 (log₂FC = –0.74) and ATP-synthase subunit ATP5MC3 (log₂FC₁₀₀ nM = –0.60) were both reduced as shown by our transcriptomic data in pancreatic β-cells (EndoCβH1), pointing to decreased mitochondrial energy capacity (Angerer et al., 2014).

These transcriptional effects are consistent with diminished respiratory efficiency and are accompanied by compensatory up-regulation of UCP3 in HepaRG cells (log₂FC ≈ 2.7–3.3), indicating an adaptive attempt to limit excessive ROS generation under OXPHOS stress (R. Z. Zhao et al., 2019).

KER 5: Disruption of OXPHOS Leading to ROS Production

The impaired electron transfer within the ETC due to disrupted OXPHOS causes electron leakage and ROS generation. This is supported by upregulated oxidative stress markers, such as such as *gpx4a* (25 nM log2FC = -1.04, adjusted p-value = 0.006; 10.000 nM log2FC = -1.33, adjusted p-value = 0.0004) in DEHP-exposed zebrafish, and GSTS (including GST02 (log2FC = 0.7) and GSTA2 in DEHP-exposed HepaRG cells, as detected in our study. These markers collectively indicate an activation of oxidative stress pathways.. Increased *gpx4a* and GSTO2indicate oxidative stress. Upregulation of uncoupling proteins (including UCP3 detected in HepaRG cells). (log2FC = 3.214) is consistent with an adaptive mitochondrial stress response aimed at partially limiting ROS generation, rather than preventing oxidative stress outright (R. Z. Zhao et al., 2019).

**Weight-of-Evidence (KER5)**

- **Evidence & Plausibility:** The impaired electron transfer within the ETC due to disrupted OXPHOS causes electron leakage and ROS generation (R. Z. Zhao et al., 2019). ETC leakage is a well-established source of ROS, and environmental toxicants like DEHP can contribute to this process (Reddam et al., 2022; Yazıcı & Sezer, 2017). The observed up-regulation of antioxidant defenses (*gpx4a*, GSTO2, GSTA2) and UCP3 from our results is consistent with compensatory responses to oxidative pressure.
- **Dose–Response & Temporal Concordance:** Oxidative stress markers increase with higher DEHP dose and after prolonged exposure, following OXPHOS impairment temporally(Y. Huang et al., 2019; E. J. Lee et al., 2024).
- **Essentiality:** ROS generation is a critical intermediate step leading to cellular injury and lipid peroxidation. Antioxidant enzyme induction suggests partial protection but does not prevent downstream damage, confirming high essentiality of this KER for progression to oxidative stress and metabolic dysfunction (San-Millán, 2023). ROS production by mitochondria can be both a function and dysfunction, playing a role in signaling but also in damage (Palma et al., 2023).

KE5: Reactive Oxygen Species (ROS) Oxidative Stress

Oxidative stress, a condition characterized by an imbalance between the generation of reactive oxygen species (ROS) and the cellular antioxidant defense mechanisms, results in the damage of cellular structures(San-Millán, 2023). ROS production typically stems from electron leakage in the electron transport chain (ETC), leading to the formation of harmful reactive molecules. In this context, exposure to DEHP showed to induce oxidative stress, confirmed by the upregulation of several key oxidative stress markers. In DEHP-exposed zebrafish, the antioxidant enzyme *gpx4a* displayed altered expression levels, with log2FC values of -1.039 at 25nM DEHP and -1.33 at 10,000nM DEHP. Similarly, in HepaRG liver cells, glutathione S-transferase enzymes (GSTs) were significantly upregulated, with GSTA2 showing log2FC values of 0.7 at 0.1nM and 0.6 at 100nM DEHP, and GSTO2 demonstrating a particularly pronounced upregulation with a log2FC value of 1.9 at 0.1nM. These findings align with the results from *in vitro* experiments conducted in OBERON which demonstrated that DEHP exposure induces oxidative stress in HepaRG cells, leading to the formation of lipid droplets—a marker of metabolic disruption (Bernal et al., 2024).

KER 6: ROS Production Leading to Decreased ATP Levels

Excessive ROS damages mitochondrial DNA and proteins, impairing OXPHOS and reducing ATP production(Maldonado et al., 2023). Disruption in OXPHOS leads to decreased ATP synthesis due to nitro-oxidative stress (Morris & Berk, 2015; Nakamura & Lipton, 2017). ROS-induced damage affects ATP synthase and other components, reducing ATP synthesis (Ebanks & Chakrabarti, 2022; Misrani, Tabassum, & Yang, 2021; Sabens Liedhegner, Gao, & Mieyal, 2012). In EndoC-βH1 pancreatic β-cells, down-regulation of ATP5MC3 reflects impaired ATP production, a finding supported by its known role in maintaining mitochondrial ATP synthesis (Murray-Cors et al., 2025). Reduced ATP levels diminish the ATP/ADP ratio, preventing closure of K_ATP_ channels, impairing membrane depolarization, and reducing Ca²⁺-mediated insulin secretion In pancreatic β-cells, ATP depletion can also result from other ROS-mediated impairments, such as loss of mitochondrial membrane potential (Gier et al., 2009; Vela-Guajardo, Garza-González, & García, 2020) or inhibition of additional ETC complexes(Sha, Hu, & Bu, 2020).

**Weight-of-Evidence (KER6)**

- **Evidence & Plausibility:** Strong mechanistic support from both our data (ATP5MC3 ↓) and literature; ROS damage to ETC proteins is well-established. Excessive ROS damages mitochondrial DNA and proteins, impairing OXPHOS and reducing ATP production(Maldonado et al., 2023; Murphy, 2009). The general plausibility of mitochondrial oxidative stress as a causative factor in cellular dysfunction, including impact on ATP production, is well-established (Giorgi et al., 2018; Kowalczyk et al., 2021). Disruption in OXPHOS leads to decreased ATP synthesis due to nitro-oxidative stress (García-Ruiz et al., 2014; Morris & Berk, 2015; Nakamura & Lipton, 2017). ROS-induced damage affects ATP synthase and other components, reducing ATP synthesis (Ebanks & Chakrabarti, 2022; Misrani et al., 2021; Sabens Liedhegner et al., 2012).
- **Dose–Response & Temporal Concordance:** As ROS accumulates, ATP decline tends to follow, potentially with a slight delay as damage accrues. In our conceptual model, once ROS surpass the buffering capacity of antioxidants, a rapid drop in ATP can ensue. There may be a dose-dependent threshold, mild ROS increases can be compensated, but beyond a certain point (e.g., at higher DEHP doses or longer exposures), ATP synthesis falls off sharply (Daussin, Boulanger, & Lancel, 2021). Temporally, decreased ATP is a downstream event manifesting after significant oxidative stress; it signals that mitochondrial function is failing (Nonarath et al., 2021; Z.-J. Yang et al., 2024). DEHP-induced ROS can lead to mitochondrial dysfunction and subsequent decrease in cellular ATP levels(Y. H. Chen et al., 2020; X. Li et al., 2014). In our zebrafish larvae, by 5 dpf exposure, signs of both ROS induction and ATP machinery downregulation were present, indicating these events occur roughly in the same window but with ROS likely initiating the ATP decline.
- **Essentiality:** Adequate ATP levels are absolutely essential for cell survival and function; thus, ATP depletion is a key step toward cell injury/death and organ dysfunction (Srivastava & Srivastava, 2017). If ROS generation is blocked or scavenged (thus preventing ATP loss), cells maintain their energy homeostasis and avoid triggering cell death pathways (Apostolova & Victor, 2015). Indeed, experiments in oxidative liver injury have shown that mitochondria-targeted antioxidants can restore ATP levels (J. Liu, Luo, Zhang, & Li, 2024; Yi et al., 2014) and prevent progression to liver injury (Hibino et al., 2023; Mukhopadhyay et al., 2012). Conversely, agents that uncouple OXPHOS (simulating ATP loss) cause liver damage even without an upstream toxicant. This underscores that ATP loss is a critical event on the route to adverse outcomes. We judge the essentiality of ROS → ATP decrease as high, without ATP depletion, the severity of downstream outcomes (like cell dysfunction, steatosis progression, etc.) would be significantly reduced.

KE6: Decreased ATP Levels

Decreased ATP levels are a direct consequence of impaired OXPHOS function. ATP concentration was not directly measured as a metabolic endpoint in the present study. Decreased ATP was predicted by the systems biology model and was supported indirectly by the downregulation of ATP5MC3 (log₂FC = −0.599) in EndoC-βH1 pancreatic β-cells. This transcriptional change is consistent with impaired ATP-synthesis capacity but does not constitute a direct measurement of ATP production. This finding is further supported by studies demonstrating that silencing or mutations affecting ATP synthase subunit c, which includes ATP5MC3, directly impair cellular ATP synthesis and overall mitochondrial function(Lovero et al., 2018).

KER 7: Decreased ATP Levels Leading to ROS Production / Oxidative Stress (Feedback Loop)

ATP depletion caused by mitochondrial dysfunction disrupts cellular processes, further impairing mitochondria and increasing ROS production. This establishes a vicious cycle, as lower ATP levels destabilize OXPHOS, promoting additional ROS production and oxidative stress (Brillo et al., 2021; Wen et al., 2025).

Indicators of impaired OXPHOS, reduced ATP synthesis, mitochondrial DNA damage, and inefficient recovery, are all associated with elevated redox state (Cichoz-Lach & Michalak, 2014; Koliaki & Roden, 2013). Additionally, inefficiency in mitochondrial OXPHOS is linked to higher ROS production, oxidative damage, and systemic inflammation (Koliaki et al., 2015).

In EndoC-βH1 pancreatic β-cells, ATP synthase (ATP5MC3) downregulation (log2FC = -0.599) suggests increased ROS and oxidative damage, reinforcing the evidence for oxidative stress due to DEHP exposure. Indeed, studies show that DEHP exposure can induce a significant increase in ROS in pancreatic β-cells (Pérez-Albaladejo, Solé, & Porte, 2020) which can contribute to mitochondrial dysfunction and impaired ATP production (Mukai, Fujimoto, & Inagaki, 2022). Furthermore, a reduction of ATP synthesis is typically accompanied by an increase in mitochondrial potential, which triggers excessive production of ROS and causes oxidative stress to further impair the survival and insulin secretion of islet β-cells (J. Li et al., 2022). In hepatocytes, DEHP-induced mitochondrial dysfunction and oxidative stress have been shown to impact ATP levels and contribute to liver damage(X. Pan et al., 2022; Sun et al., 2023; P. Xu, Su, Ling, Wang, & Zhang, 2024) .

These findings collectively demonstrate that energy failure promotes persistent oxidative stress, reinforcing mitochondrial dysfunction in a vicious cycle that sustains damage beyond the initial insult.

**Weight-of-Evidence (KER7)**

- **Evidence and Plausibility:** There is strong mechanistic and empirical support for the bidirectional coupling between ATP depletion and oxidative stress, verified in both in-vitro and in-vivo models. Once ATP declines, the mitochondrial membrane potential becomes unstable, increasing electron leakage and ROS formation (Palma et al., 2023; Wen et al., 2025). This mechanism is observed in various tissues, including the liver, where mitochondrial ROS generation triggers inflammatory responses and tissue injury (Mukhopadhyay et al., 2012).
- **Dose–Response and Temporal Concordance:** ROS increases persist once ATP levels drop below a functional threshold, producing a sustained feedback loop that is largely independent of further dose escalation. Temporally, this amplification follows earlier oxidative events and consolidates chronic mitochondrial stress (Wen et al., 2025).
- **Essentiality:** Breaking the feedback loop, by restoring ATP synthesis or suppressing ROS accumulation, interrupts mitochondrial deterioration, confirming the high essentiality of this link in maintaining energy homeostasis (Palma et al., 2023; Trushina, Nguyen, & Trushin, 2023). Indeed, experiments in oxidative liver injury have shown that mitochondria-targeted antioxidants can restore ATP levels and prevent progression to liver injury (J. J. W. Lee et al., 2024; Yi et al., 2014). The vicious cycle underscores why prolonged or severe DEHP exposure can lead to escalating mitochondrial damage even if initial insults were moderate (X. Pan et al., 2022; J. Xu et al., 2021; Haoyang Zhang et al., 2023).

KER 8: Decreased ATP Levels Leading to NAFLD

This Key Event Relationship (KER) links mitochondrial energy collapse to the manifestation of non-alcoholic fatty liver disease (NAFLD). In the Adverse Outcome Network (AON), decreased ATP represents a downstream event following the disruption of oxidative phosphorylation (OXPHOS) and a key transition point toward hepatic pathology.
The adverse outcome of interest in this branch is NAFLD, characterized by hepatic steatosis with progression to inflammation and injury. KER8 posits that sustained loss of ATP (energy failure) in hepatocytes leads to NAFLD development. There is a strong mechanistic basis for this: energy deficits impair all active cellular processes, including fatty-acid oxidation and export, leading to lipid accumulation and hepatocellular injury. Specifically, ATP depletion reduces β-oxidation capacity, induces endoplasmic-reticulum stress, and weakens antioxidant defenses, thereby driving the transition from steatosis to steatohepatitis (Begriche et al., 2013; García-Ruiz et al., 2014; Hughey, Puchalska, & Crawford, 2022; K. Lee et al., 2018; Shum, Ngo, Shirihai, & Liesa, 2021).

As demonstrated in KE4 (Disruption of OXPHOS), DEHP exposure caused down-regulation of mitochondrial respiratory chain components, indicating reduced energy output and ATP synthesis. This mitochondrial impairment limits fatty-acid oxidation and enhances lipid-droplet accumulation, consistent with early NAFLD phenotypes. In our integrated systems-biology model, ATP depletion coincided with the activation of fibrogenic and inflammatory signaling pathways, confirming a temporal association between energy collapse and disease initiation.

Although direct ATP quantification was not performed, the consistent pattern of OXPHOS subunit loss, oxidative stress, and lipid accumulation strongly infers mitochondrial energy failure. These findings align with literature showing that persistent ATP deficiency disrupts hepatic metabolic homeostasis and promotes lipid accumulation and cell injury (García-Ruiz et al., 2014; Hughey et al., 2022).

**Weight-of-Evidence (KER8)**

- **Evidence and Plausibility:** The mechanistic link between decreased ATP and NAFLD is biologically plausible and well supported. Reduced mitochondrial ATP generation limits the energy supply required for lipid oxidation, export, and detoxification (Hughey et al., 2022). The observed OXPHOS disruption under DEHP exposure mirrors mechanisms described in NAFLD and NASH (Begriche et al., 2013; K. Lee et al., 2018).
- **Consistency and Temporal Concordance:** The development of NAFLD is chronic and cumulative. Evidence from in vivo and in vitro systems indicates that mitochondrial dysfunction and ATP loss precede histological NAFLD, suggesting that energy depletion is an early and persisting driver (Abdelmalek et al., 2012; A. H. Lee et al., 2022; Teodoro, Rolo, Duarte, Simões, & Palmeira, 2008). The relationship is temporally consistent with the sequence of events observed in the AON, ATP depletion follows OXPHOS disruption but precedes overt liver injury, supporting its causal position. Mitochondrial dysfunction is a key driver in NAFLD progression (Legaki et al., 2022; Zheng, Wang, Wu, & Wang, 2023)
- **Essentiality:** Mitochondrial ATP generation is central to maintaining hepatic metabolic homeostasis. Restoration of mitochondrial function, through antioxidants, cofactors, or enhanced mitochondrial efficiency, can reverse steatotic changes (Grattagliano et al., 2019), while sustained ATP depletion accelerates lipid accumulation and oxidative injury(Abdelmalek et al., 2012; Myint et al., 2023). Therefore, the essentiality of this event for NAFLD progression is high, as mitochondrial dysfunction and energy collapse are recognized as key determinants of disease severity (Ajaz et al., 2021; Hughey et al., 2022).

### KE7: Mitochondrial Dysfunction

Mitochondrial dysfunction represents a central downstream key event in the DEHP-induced metabolic disruption network, integrating oxidative stress, energy imbalance, and loss of redox control. It acts as a convergence point of the preceding events: Disruption of OXPHOS (KE4), ROS production (KE5), and Decreased ATP levels (KE6), and reflects a systemic failure of mitochondrial homeostasis.

As demonstrated in KE4 and KE6, DEHP exposure significantly impairs mitochondrial respiration and ATP generation. These impairments disrupt electron flow through the respiratory chain, leading to electron leakage and excessive formation of reactive oxygen species (ROS). Because the electron transport chain (ETC) is tightly coupled to ATP turnover, any imbalance between electron transfer and ATP synthesis initiates mitochondrial stress and promotes further oxidative injury.

DEHP exposure perturbs OXPHOS function and mitochondrial regulatory systems, notably the uncoupling proteins (UCPs). Activation of UCP2 and UCP3 serves a compensatory role by lowering mitochondrial membrane potential and limiting ROS buildup, a widely recognized adaptive mechanism to oxidative stresss (R. Z. Zhao et al., 2019). In HepaRG cells, UCP3 showed significant upregulation at all three concentrations tested (log₂FC = 3.214, 3.255, and 2.669 at 100 pM, 100 nM, and 10 µM, respectively), consistent with a cellular attempt to mitigate ROS production.

Simultaneously, inflammatory and hypoxia-responsive pathways were activated, consistent with oxidative and nitrosative stress(Begriche et al., 2013). In HepaRG cells, TNFAIP1 increased at all concentrations (log₂FC₁₀₀ pM = 1.224; log₂FC₁₀₀ nM = 0.975; log₂FC₁₀ µM = 0.802); NOSIP was upregulated at 100 pM (log₂FC = 0.796); and HIF3A increased significantly at 10 µM (log₂FC = 1.47). Together, these findings indicate activation of TNF-α/iNOS and HIF-1α–mediated pathways, which further destabilize mitochondrial respiration and exacerbate ROS/RNS production (Begriche et al., 2013).

Moreover, ROS-induced mtDNA damage impairs ETC complex activity and ATP synthesis, reinforcing the oxidative stress cycle (Mukai et al., 2022; Palma et al., 2023; Wen et al., 2025). Decreased mitochondrial protein synthesis, OXPHOS inefficiency, and redox imbalance are all consistent with reduced oxidative capacity (Cichoz-Lach & Michalak, 2014; Iqbal et al., 2005; Mantena et al., 2008; C. Zhang et al., 2022). Altogether, these alterations depict mitochondrial dysfunction as both a downstream manifestation and an amplifier of metabolic stress.

KE 8: Decreased Serine Levels

L-serine, a critical amino acid involved in protein, nucleotide, and lipid synthesis, was found to be significantly downregulated in both zebrafish larvae and HepaRG cells exposed to DEHP. In zebrafish, exposure to 10μM and 25nM of DEHP led to notable decreases in L-serine levels, with log2FC values of -1.729 and -1.777, respectively. This downregulation suggests that DEHP interferes with serine biosynthesis or turnover, potentially disrupting metabolic pathways crucial for sphingolipid and cellular membrane integrity.

Similarly, in HepaRG cells, L-serine was downregulated with log2FC values of -1.102, -1.372, and -0.633 after exposure to 0.1nM, 100nM, and 10,000nM DEHP, respectively. This dose-dependent effect highlights DEHP's broad impact on amino acid homeostasis, which likely contributes to disruptions in sphingolipid metabolism and increases oxidative stress. The depletion of serine, a precursor in sphingolipid synthesis, may also affect the production of critical molecules like ceramides and S1P, exacerbating metabolic dysfunctions linked to DEHP exposure.

KER 9: Increased Oxidative stress Leads to Decreased Serine levels

This KER links oxidative stress (KE5) to decreased serine levels (KE8). Under oxidative conditions, elevated ROS disrupts the serine biosynthesis pathway by impairing the rate-limiting enzyme phosphoglycerate dehydrogenase (PHGDH), which catalyzes the conversion of 3-phosphoglycerate to serine (Mullarky & Cantley, 2015). Additionally, oxidative stress increases cellular demand for antioxidants like glutathione, which is synthesized from serine, further depleting serine levels (Forman, Zhang, & Rinna, 2009; T.-Y. Lin et al., 2016). These mechanisms jointly reduce L-serine availability, as also observed in both DEHP-exposed zebrafish and HepaRG cells, where oxidative stress coincided with decreased serine levels in a consistent, dose-related pattern.

**Weight-of-Evidence (KER9)**

- **Evidence and Plausibility:** The causal linkage between oxidative stress and serine depletion is biologically plausible and well supported. PHGDH is a key enzyme in serine biosynthesis, and its activity can be impacted by oxidative stress(A. M. Li & Ye, 2020; Mullarky & Cantley, 2015; Spillier et al., 2019). ROS inhibits PHGDH activity and diverts serine and glycine toward glutathione synthesis, reducing intracellular serine concentrations (Mullarky & Cantley, 2015; X. Zhao, Fu, Du, & Xu, 2020). This mechanism is widely described in the literature (Handzlik & Metallo, 2023; Holeček, 2022) and consistent with the decreases detected in both in vitro and in vivo DEHP models.
- **Dose–Response and Temporal Concordance:** Serine depletion follows the rise of oxidative stress markers, indicating clear temporal order. Higher DEHP concentrations, associated with stronger oxidative stress (E. J. Lee et al., 2024; X. Pan et al., 2022), corresponded to greater serine decreases, suggesting a threshold at which antioxidant defenses become saturated and serine utilization intensifies . The direct link between dose-dependent oxidative stress and subsequent serine depletion in response to DEHP is further supported by general mechanisms of oxidative stress impacting serine metabolism (Mullarky & Cantley, 2015).
- **Essentiality:** Maintaining serine homeostasis mitigates oxidative injury by sustaining glutathione and sphingolipid synthesis. Conversely, impaired serine metabolism amplifies oxidative stress and energy imbalance(Handzlik & Metallo, 2023; Shan & Hoffman, 2024). Although not sufficient alone to trigger an adverse outcome, this KER plays a facilitative role in sustaining redox and metabolic disruption. Serine supplementation or restoration of PHGDH activity has been shown to partially reverse oxidative damage (Goyal et al., 2025; L. He, Ding, Zhou, Li, & Yin, 2023; X. Liu et al., 2025). Essentiality is therefore assessed as **moderate**.

KER 10: Increased Oxidative stress Leads to phosphorylation of serine residues

Oxidative and inflammatory stress promotes inhibitory serine phosphorylation of insulin receptor substrates (IRS1/IRS2) through activation of stress-responsive kinases (e.g. JNK, IKKβ, PKC).(Hongjuan Fang et al., 2020; Feng et al., 2020; Tanti & Jager, 2009; Vinayagamoorthi, Bobby, & Sridhar, 2008). This post-translational modification attenuates downstream insulin receptor signaling and represents a key molecular mechanism linking cellular stress to impaired insulin signaling pathways.

**Weight-of-Evidence (KER10)**

- **Evidence and Plausibility:** Literature demonstrates that mitochondrial dysfunction, reactive oxygen species (ROS), lipid-induced stress, and inflammatory signaling converge on stress kinase activation, resulting in inhibitory serine phosphorylation of IRS proteins(Begriche et al., 2013; Hongjuan Fang et al., 2020; Tanti & Jager, 2009; Zheng et al., 2023). This mechanism is well established across metabolically relevant tissues, although its downstream functional consequences are tissue-specific.
- **In the present study:**

In pancreatic β-cells, DEHP exposure has been associated with increased oxidative stress, consistent with activation of serine kinases that promote inhibitory IRS phosphorylation. Consistently, reduced IRS1 expression (KE10) was observed in EndoC-βH1 pancreatic β-cells, suggesting attenuation of IRS-mediated signaling. Supporting these molecular alterations, data generated within the OBERON project indicate a reduction in insulin secretion following DEHP exposure in EndoC-βH1 pancreatic β-cells. Importantly, in pancreatic β-cells, IRS-dependent signaling primarily regulates insulin secretory function, stress responses, and cell survival rather than peripheral insulin sensitivity; therefore, the observed IRS alterations are unlikely to directly reflect classical systemic insulin resistance, but they may impair the β-cells’ ability to mount a compensatory response under conditions of increased insulin demand.

In HepaRG cells, DEHP exposure induced pronounced mitochondrial dysfunction, ROS generation, ATP depletion, and sphingolipid perturbations, including ceramide accumulation. Although inhibitory IRS serine phosphorylation was not directly measured in HepaRG cells, these molecular perturbations constitute well-established upstream drivers of IRS inhibition in hepatocytes, providing indirect but biologically coherent support for this KER in the hepatic context**.**

**Dose–Response and Temporal Concordance:** Across experimental models, oxidative stress and inflammatory signaling occurred downstream of early mitochondrial dysfunction and upstream of downstream insulin-signaling–related outcomes, supporting appropriate temporal ordering of this KER. Dose-dependent increases in ROS and lipid perturbations further support concordance with the proposed relationship. IRS phosphorylation follows oxidative stress, appearing after ROS and cytokine elevation (Tanti & Jager, 2009; Vinayagamoorthi et al., 2008). Temporal coherence is strong, as IRS modification occurs within hours to days post-exposure (Vinayagamoorthi et al., 2008). Studies show that increased ROS levels can precede the onset of insulin resistance (Deng et al., 2018; Hongjuan Fang et al., 2020)

- **Essentiality:** Blocking this phosphorylation (e.g., through JNK inhibition or antioxidants) restores insulin sensitivity in peripheral tissues, highlighting its essential role in metabolic disruption (Feng et al., 2020; Vinayagamoorthi et al., 2008). Essentiality: High.

KE 9: IRS Serine Phosphorylation (IRS1/IRS2)

The phosphorylation of serine residues on insulin receptors substrates (IRS1 and IRS2) is triggered by the activation of stress- and inflammation-responsive serine kinases (Gual, Grémeaux, Gonzalez, Le Marchand-Brustel, & Tanti, 2003; Morino et al., 2008; Tanti & Jager, 2009; Weigert et al., 2005). This phosphorylation is facilitated by the activation of inflammatory signaling pathways. Notably, consistent with inflammatory signaling activation, several TNF superfamily members were found to be upregulated in HepaRG experiments, including TNFRSF14 (log2FC0.1nM = 1.7), TNFRSF1B (log2FC0.1nM = 1.1), and TNFSF13 (log2FC0.1nM = 1.51 and log2FC100nM = 1.54) at least at one concentration. Additionally, fatty acid derivatives such as ceramides and diacylglycerol (statistically significantly upregulated at all concentrations in HepaRG), as well as increased levels of ROS, further promote this process (Tanti & Jager, 2009). These lipid changes are consistent with the ceramide accumulation described in KE1 and provide a mechanistic link between early FFA rerouting and impaired insulin signalling.

KE 10: Decreased Insulin Receptor Function

IRS1 expression was reduced in pancreatic β-cells treated with 1, 10, and 100nM of DEHP, with log2FC values of -0.3778, -0.2263, and -0.271, respectively. This reduction is anticipated to lead to impaired downstream insulin signaling, affecting pancreatic β-cell function (Kulkarni et al., 1999).

KE11: Sphingosine-1-Phosphate (S1P) disruption

Sphingosine-1-phosphate (S1P) is a key bioactive lipid regulating cell survival, proliferation, immune modulation, and vascular function through binding to G-protein–coupled receptors (S1P₁–S1P₅)(Ishii, Fukushima, Ye, & Chun, 2004). S1P homeostasis depends on balanced synthesis by sphingosine kinases (SphK1/2) and degradation via phosphatases and lyases, tightly linked to ceramide and sphinganine metabolism (Wigger, Schumacher, Schneider-Schaulies, & Kleuser, 2021).

DEHP exposure disrupted this balance in both zebrafish larvae and HepaRG cells. In zebrafish (5 dpf), S1P and its precursor sphinganine were significantly downregulated, suggesting impaired de novo sphingolipid synthesis and signaling (Hisano et al., 2015). Specifically, sphinganine levels were reduced with log2FC values of -3.962 at 10 μM DEHP and -2.232 at 25 nM, while S1P was downregulated with log2FC values of -1.746 and -1.267 at the same concentrations Conversely, in HepaRG cells, S1P species showed chain-length–dependent alterations: S1P (d19:1-P) was upregulated, with log2FC values of 0.253, 1.72, and 1.11 at DEHP concentrations of 0.1, 100, and 10,000 nM, respectively, whileS1P (d16:1) was significantly downregulated, with log2FC values of -4.22, -3.75, and -2.47 at the same concentrations, Such bidirectional modulation reflects adaptive versus pathological responses depending on cellular stress context, increased S1P may promote survival signaling, whereas depletion of canonical S1P species compromises lipid and hepatic insulin homeostasis. The S1P (d19:1-P) increase and S1P (d16:1) decrease is consistent with metabolic reprogramming under oxidative and inflammatory stress (Kleuser, 2018). Increases in specific ceramide species observed in HepaRG cells in our study further suggest a rerouting of de novo sphingolipid synthesis toward ceramide accumulation under DEHP-induced stress. This shift occurs in parallel with qualitative alterations in sphingosine-1-phosphate(Kasumov et al., 2015; Le Mentec, 2023; Zhou et al., 2018).

KER 11: Decreased serine levels Lead to S1P

Serine is a precursor in sphingolipid biosynthesis; its depletion limits formation of ceramides and sphingosine-1-phosphate (S1P), key mediators of lipid signaling and membrane stability(Green, Maceyka, Cowart, & Spiegel, 2021). In the present dataset, this is reflected by combined decreases in serine and palmitate together with altered S1P species and increased ceramides in HepaRG cells, consistent with a reprogrammed sphingolipid pool under DEHP-induced oxidative stress.

**Weight-of-Evidence (KER11)**

- **Evidence and Plausibility:** Serine combines with palmitoyl-CoA to produce sphingolipids; thus, reduced serine availability directly lowers S1P synthesis (Esch et al., 2020; Harrison, Dunn, & Campopiano, 2018; Mah, Febbraio, & Turpin-Nolan, 2021). In both zebrafish and liver cells, DEHP reduced serine and palmitate levels, impairing S1P production. The concurrent increase in hepatic ceramides supports the notion that limited serine availability constrains S1P synthesis while favouring accumulation of upstream sphingolipid intermediates, further reinforcing the mechanistic link between serine depletion and S1P disruption.
- **Dose–Response and Temporal Concordance:** The magnitude of S1P reduction paralleled the severity of serine depletion, with consistent effects across concentrations and models.
- **Essentiality:** Serine and S1P balance is central to maintaining membrane integrity and metabolic signaling. Restoration of serine reverses downstream lipid imbalance (Green et al., 2021) Essentiality: Moderate.

KER 12: IRS Serine Phosphorylation (IRS1/IRS2) leads to decreased insulin receptor-mediated signaling

This KER represents the inhibitory outcome of IRS serine phosphorylation on insulin receptor (IR) signaling mediated signal transduction. Serine-phosphorylated IRS1/2 exhibit reduced ability to propagate insulin receptor signaling through the IR–PI3K–Akt axis, resulting in impaired insulin signaling. The functional consequences of this disruption are tissue-specific, affecting hepatic glucose metabolic regulation in hepatocytes whereas in pancreatic β-cells, it compromises insulin signaling, which can negatively affect β-cell function, including insulin secretion, survival, and growth.

**Weight-of-Evidence (KER12)**

- **Evidence and Plausibility:**. Our pancreatic β-cell data show a decrease in IRS1 expression, which may be associated with signals that promote serine phosphorylation and a reduction in IR/PI3K pathway components. In this cellular context, disruption of IRS-mediated signaling primarily affects β-cell function rather than classical insulin resistance. Literature demonstrates that serine-phosphorylated IRS proteins have reduced capacity to interact with the insulin receptor and downstream effectors, thereby impairing insulin signal transduction (Boura-Halfon & Zick, 2009; Tanti & Jager, 2009). Furthermore, several studies have reported that DEHP exposure disrupts PI3K/Akt signaling in insulin-responsive tissues, including the liver in animal studies (Rajagopal, Bhaskaran, & Karundevi, 2019b; M. Wang et al., 2023)
- **Dose–Response and Temporal Concordance:** The inhibition of insulin signaling correlates with IRS phosphorylation and inflammatory activation, both of which increase with DEHP dose and exposure duration
- **Essentiality:** (Alonso-Magdalena et al., 2018; Wei et al., 2022). Disruption of IRS-mediated signaling is critical for metabolic outcomes. While our data are limited to pancreatic β-cells, literature evidence indicates that similar disruptions in insulin-responsive tissues, such as the liver, contribute to glucose intolerance and steatosis. Interventions that enhance insulin sensitivity (e.g., thiazolidinediones or antioxidants) may alleviate downstream liver fat accumulation (Al-Muzafar & Amin, 2018; Buckingham, 2005; Yki-Järvinen, 2009) and management of oxidative stress (e.g., with antioxidants) may help control the progression of DEHP-induced liver damage(E. J. Lee et al., 2024)Essentiality**:** High

KER 13: S1P leads to impaired insulin receptor signaling

Elevated sphingosine-1-phosphate (S1P) impairs insulin receptor signaling through activation of inflammatory pathways. S1P acting via receptors such as S1PR2 and S1PR3 can activate NF-κB and PI3K/Akt signaling, both recognized contributors to insulin resistance and impaired insulin receptor activity (Fayyaz, Henkel, et al., 2014; Hait & Maiti, 2017; Takahashi et al., 2017).
S1P-mediated inflammation has been linked specifically to hepatic insulin resistance and metabolic dysfunction (Fayyaz, Henkel, et al., 2014; Kleuser, 2018) .These mechanisms collectively support the causal link between altered S1P and decreased insulin receptor activity.

**Weight-of-Evidence (KER13)**

- **Evidence and Plausibility:** Multiple studies show that elevated S1P activates inflammatory pathways that impair insulin receptor signaling. This provides strong biological plausibility that S1P disrupts insulin receptor activity (Z. Fang, Pyne, & Pyne, 2019; Juchnicka, Kuźmicki, & Szamatowicz, 2021; Kitada et al., 2016). S1P and ceramides, derived from sphingolipids, play crucial roles in regulating insulin signaling pathways and glucose metabolism (Ali-Berrada et al., 2023; Guitton et al., 2020; Juchnicka et al., 2021)
- **Dose–Response and Temporal Concordance:** In DEHP-exposed liver cells, S1P dysregulation occurs within the same exposure window as inflammatory activation and downstream insulin signaling impairment, supporting the expected temporal order. Although quantitative dose response is limited, the directionality is consistent.
- **Essentiality:** S1P is not the sole determinant of insulin resistance but acts as an important amplifier of insulin receptor impairment through inflammation (Z. Fang et al., 2019; Fayyaz, Japtok, & Kleuser, 2014). Essentiality is therefore moderate.

### KER 14: S1P Disruption Leads to Obesity

Dysregulated sphingosine-1-phosphate (S1P) signaling contributes to adiposity and systemic metabolic dysfunction. S1P acts through five receptors (S1PR1–S1PR5), with S1PR2 generally promoting adipogenesis and insulin resistance, whereas S1PR1 activation exerts protective, anti-obesogenic effects (Kajita et al., 2024; Kitada et al., 2016). Elevated S1P levels have been associated with increased adipocyte hypertrophy, impaired glucose tolerance, and inflammation in both animal and human studies (W. Chen, Lu, Yang, Xiang, & Peng, 2016; Kowalski, Carey, Selathurai, Kingwell, & Bruce, 2013). Pharmacological modulation supports this directionality: S1PR2 antagonism (JTE-013) or S1PR1 agonism (SEW-2871) reduces fat accumulation and improves glucose tolerance in obese mice (Asano et al., 2023; Kitada et al., 2016). S1P also influences adipogenesis by regulating PPARγ expression through S1PR3 (Chakrabarty et al., 2022), and bioactive lipids derived from ceramides, including S1P, modulate insulin signaling and glucose metabolism (Guitton et al., 2020; Juchnicka et al., 2021).

In the context of DEHP exposure, in our study, S1P dysregulation occurred in both zebrafish and HepaRG cells, with some species (e.g., d19:1-S1P) upregulated and others (e.g., d16:1-S1P) downregulated, indicating perturbation of sphingolipid metabolism.In accordance to our results, S1P dysregulation, including altered sphingolipid metabolic enzymes, has been observed in rat liver and Daphnia magna (Cho et al., 2022; Jo et al., 2011). Phthalates have also been linked to shifts in lipidomic markers such as ceramides and other sphingolipids (Ashrap et al., 2022), providing a biologically coherent link between DEHP exposure and S1P-mediated metabolic dysfunction.

**Weight-of-Evidence (KER14)**

- **Evidence and Plausibility** There is strong mechanistic plausibility that altered S1P signaling promotes adipogenesis and obesity. S1P–S1PR2 signaling drives adipocyte hypertrophy and insulin resistance, whereas S1PR1 activity protects against fat accumulation (Asano et al., 2023; W. Chen et al., 2016; Kitada et al., 2016). Elevated circulating S1P levels correlate with BMI, total body fat, waist circumference, fasting insulin, HOMA-IR, and HbA1c in humans (Kowalski et al., 2013). S1P, ceramide, and related sphingolipids regulate key metabolic pathways, including lipid storage, insulin sensitivity, and inflammatory signaling (Guitton et al., 2020; Juchnicka et al., 2021; Kajita et al., 2024)
- **Dose–Response and Temporal Concordance** In experimental models, higher S1P increases adiposity, while S1PR2 blockade or S1PR1 activation reverses obesity and metabolic dysfunction (Asano et al., 2023; Kitada et al., 2016; S. Y. Park et al., 2012). These interventions demonstrate dose-responsiveness and temporal alignment: modifying S1P signaling changes fat accumulation and glucose tolerance over time.
- **Essentiality** Pharmacological inhibition of S1PR2 or activation of S1PR1 reduces obesity and improves metabolic outcomes, indicating essentiality of S1P signaling in driving adiposity (Asano et al., 2023; Kitada et al., 2016)While direct DEHP-specific tests are limited, DEHP-induced increases in adiposity and adipocyte dysfunction (Klöting et al., 2015) combined with sphingolipid alterations support that S1P acts as a critical mediator of this metabolic outcome. Moderate to strong

KE 12: Impaired Insulin Signaling

Impaired insulin signaling, marked by reduced cellular response to insulin, disrupts glucose homeostasis and contributes to insulin resistance, especially in hepatic cells (Biddinger & Kahn, 2006). These disruptions affect pathways downstream of Akt activation, including GSK3 and mTORC1. In liver cells exposed to 0.1nM DEHP, in our study, mTOR was significantly upregulated (log2FC = 0.829), while FOXO6, a critical glucose regulator, was also upregulated (log2FC = 0.899). mTOR upregulation in response to DEHP exposure, has been previously reported as well (Huff, Da Silveira, Carnevali, Renaud, & Hardiman, 2018; Klöting et al., 2015). Dysregulated FOXO1 influenced by DEHP exposure, promotes hepatic gluconeogenesis, leading to insulin resistance (Rajagopal et al., 2019b; M. Wang et al., 2023). The impaired insulin signaling pathways contribute to metabolic dysfunctions such as hepatic steatosis and glucose intolerance, exacerbating conditions like type 2 diabetes (Mu et al., 2019; Niranjan, 2023; Sajan, Ivey, & Farese, 2015; Scoditti, Sabatini, Carli, & Gastaldelli, 2024; Titchenell et al., 2016). Lipid metabolites like non-esterified fatty acids (NEFA) further aggravate insulin resistance by generating diacylglycerol (DAG) and ceramides, which disrupt insulin receptor activity via protein kinase C ε (PKCε) activation and inhibit Akt through protein phosphatase 2A (PP2A) (DeFronzo, 2010; Jornayvaz & Shulman, 2012; Petersen & Shulman, 2017; Wigger et al., 2021). The role of sphingosine-1-phosphate (S1P) is complex; SphK2 activity and Sph levels impair insulin signaling, while stimulation of S1P1/S1P3 enhances insulin sensitivity, particularly when bound to apoM. In contrast, S1PR2 stimulation via S1P bound to albumin reduces insulin signaling (Wigger et al., 2021). The functional consequences of impaired insulin signaling are tissue-specific, affecting gluconeogenic control in hepatocytes and insulin secretion capacity in pancreatic β-cells.

KER 15: Decreased Insulin Receptor Function Leads to Impaired insulin signaling

A reduction in insulin receptor (INSR) availability or function diminishes the capacity of cells to respond to insulin, directly impairing the insulin signaling cascade(Biddinger & Kahn, 2006; Rajagopal, Bhaskaran, & Karundevi, 2019a; Rengarajan, Parthasarathy, Anitha, & Balasubramanian, 2007). This disruption limits PI3K/Akt activation, and alters downstream metabolic regulators such as GSK3, mTORC1, and FOXO transcription factors. These changes contribute to dysregulated glucose metabolism and overall insulin responsiveness, key features of metabolic dysfunction and type 2 diabetes. This relationship is biologically coherent with established mechanisms showing that reduced INSR/IRS1 interaction, whether via receptor downregulation, inhibitory serine phosphorylation, or lipid-mediated interference, leads to impaired insulin signaling (Biddinger & Kahn, 2006; Chandrasekaran & Weiskirchen, 2024). Additional lipotoxic mediators such as DAG and ceramides further disrupt receptor function through PKCε activation and PP2A-mediated inhibition of Akt (Jornayvaz & Shulman, 2012; Petersen & Shulman, 2017; Zabielski et al., 2018), while sphingolipid mediators like S1P can either impair or enhance insulin signaling depending on the receptor subtype involved (Wigger et al., 2021).

**Weight-of-Evidence (KER 15)**

- **Evidence and Plausibility** The causal link between decreased insulin receptor function and impaired insulin signaling is well established. Literature consistently demonstrates that reductions in INSR/IRS1 availability or functionality diminish the initiation of insulin signaling and promote metabolic dysfunction(Biddinger & Kahn, 2006; Hongjuan Fang et al., 2020; Rajagopal et al., 2019a; Rengarajan et al., 2007). Mechanistically, inflammatory signaling, serine phosphorylation of IRS proteins(Tanti & Jager, 2009), lipotoxic metabolites such as DAG and ceramides(Jornayvaz & Shulman, 2012; Petersen & Shulman, 2017), and S1P receptor–mediated pathways (Wigger et al., 2021)all interfere with receptor activity or downstream signal transduction. This mechanistic convergence provides strong plausibility.
- **Dose–Response and Temporal Concordance** Across experimental systems and published studies, decreased insulin receptor/IRS1 function occurs following upstream stressors such as oxidative stress, inflammatory signaling, and sphingolipid imbalance, and precedes measurable impairments in insulin signaling. Literature reports dose-dependent effects of endocrine disruptors on INSR/IRS pathways, matching the expected sequence of events(Rajagopal et al., 2019a; Rengarajan et al., 2007; Tanti & Jager, 2009; M. Wang et al., 2023) . Temporal concordance is therefore supported by published studies.
- **Essentiality**. Functional insulin receptors are required to activate PI3K/Akt, regulate glucose metabolism, and maintain metabolic homeostasis. Pharmacological or genetic restoration of IRS/INSR function improves insulin signaling and reduces downstream metabolic dysfunction in multiple models (Biddinger & Kahn, 2006; Sargis & Simmons, 2019) DEHP and related EDCs have been shown to impair both hepatic and pancreatic β-cell function (Al-Abdulla, Ferrero, Soriano, Boronat-Belda, & Alonso-Magdalena, 2022)), reinforcing this KER’s critical role in the AON. Essentiality is high

KER 16: FFA accumulation leads to Steatosis

Accumulation of free fatty acids (FFAs) within hepatocytes is a direct and well-established driver of steatosis. Excess FFAs are converted to triglycerides and stored as lipid droplets, while concurrently activating lipogenic regulators such as PPARγ and SREBP-1c that further enhance lipid synthesis (Geisler & Renquist, 2017; Gluchowski et al., 2019). Under DEHP exposure, lipid metabolism becomes dysregulated—mitochondrial β-oxidation is impaired, oxidative stress increases, and PPAR pathways become overactivated—amplifying FFA accumulation and accelerating steatosis (Bernal et al., 2024; Y. Q. Huang et al., 2022). FFAs also trigger NF-κB–mediated inflammatory responses, which aggravate lipid overload and contribute to liver injury (Bernal et al., 2024; Geisler & Renquist, 2017; Y. Q. Huang et al., 2022; Liang, Liang, Zhang, Yan, & Luan, 2024). In line with this, the HepaRG metabolomics data showed that FFA overload under DEHP exposure is accompanied by consistent increases in several ceramide species, supporting a lipotoxic shift from simple FFA storage toward sphingolipid-mediated triglyceride accumulation and steatosis.

**Weight-of-Evidence (KER 16)**

- **Evidence and Plausibility** FFAs serve as substrates for triglyceride formation, and their intracellular surplus is a canonical cause of hepatic steatosis. Lipotoxic signaling via PPARγ/SREBP-1c promotes de novo lipogenesis, while impaired oxidation under toxicant exposure further increases lipid burden (Bernal et al., 2024; Gluchowski et al., 2019)
- **Dose–Response and Temporal Concordance** Steatosis consistently follows periods of elevated FFA availability across models. In DEHP studies, lipid accumulation occurs after upstream disturbances in lipid uptake, oxidation, and signaling, reflecting the expected temporal ordering(Angrish, Kaiser, McQueen, & Chorley, 2016; Liang et al., 2024).
- **Essentiality** Reducing FFA supply or enhancing β-oxidation prevents steatosis, while experimentally increasing FFAs reliably induces lipid droplet formation—demonstrating essentiality of this relationship(Gluchowski et al., 2019). DEHP-related mitochondrial dysfunction and lipogenic activation reinforce this causal link . Essentiality is high.

KER 17: Impaired insulin signaling leads to hepatic insulin resistance

Impaired insulin in hepatocytes disrupts normal metabolic regulation, leading to hepatic insulin resistance. Specifically, reduced insulin receptor and IRS-mediated signaling limits downstream PI3K/Akt activation, resulting in impaired suppression of gluconeogenic pathways and dysregulated hepatic glucose metabolism. This signaling disruption contributes to increased expression of gluconeogenic regulators such as PCK1 and G6PC and promotes excessive hepatic glucose production, a hallmark of hepatic insulin resistance. (Santoleri & Titchenell, 2019; S. Yu, Meng, Xiang, & Ma, 2021).

DEHP exposure exacerbates hepatic insulin resistance through multiple mechanisms, including oxidative damage that impairs the PI3K/Akt/signaling axis required for insulin-mediated suppression of gluconeogenesis (G. Li et al., 2021; L. Li et al., 2023). Additionally, DEHP-induced FoxO1 upregulation promotes gluconeogenesis, further elevating glucose levels (Rajagopal et al., 2019b; Wei et al., 2022). DEHP also activates PPARγ, disrupting IRS1 phosphorylation and insulin signaling in liver cells (W. Zhang et al., 2017). Systemic metabolic consequences may be further influenced by DEHP-induced β-cell dysfunction including reduced insulin secretion(Al-Abdulla et al., 2022; Y. Lin et al., 2011). Together, these disruptions emphasize DEHP’s role in promoting metabolic dysfunction linked to diseases such as NAFLD and type 2 diabetes (Bernal et al., 2022; F. Wang et al., 2019).

**Weight-of-Evidence (KER17)**

- **Evidence and Plausibility:** The causal link between impaired insulin signaling and hepatic insulin resistance is strongly supported across metabolic studies, where disruption of IRS1/IRS2, PI3K/Akt, and downstream gluconeogenic control reliably produces hepatic insulin resistance (Santoleri & Titchenell, 2019; D. Yu et al., 2021). The DEHP-induced effects described above, altered IRS function, reduced Akt signaling, FoxO1 dysregulation, and, indirectly, pancreatic β-cell impairment leading to reduced insulin secretion, are all consistent with this well-characterized mechanism and reinforce the biological plausibility of this KER(Al-Abdulla et al., 2022; L. Li et al., 2023; W. Zhang et al., 2017).
- **Temporal and Dose–Response Concordance:** Studies show that insulin signaling defects appear before or alongside the emergence of hepatic insulin resistance, and severity increases with stronger upstream disruption (Kim, Park, Bae, Lim, & Hong, 2013; L. Li et al., 2023)
- **Essentiality:** Restoring IRS1/IRS2 activity or PI3K/Akt signaling improves hepatic insulin sensitivity in experimental systems (Santoleri & Titchenell, 2019), supporting moderate-to-strong essentiality.

KE 13: Steatosis

Steatosis, the accumulation of fat within liver cells, is often triggered by disrupted fatty acid metabolism and is closely linked to insulin resistance. This metabolic imbalance contributes to the buildup of free fatty acids (FFAs) in the liver, which activates pathways associated with oxidative stress, inflammation, and potentially fibrosis and apoptosis (Pafili & Roden, 2021). Four primary mechanisms drive triglyceride buildup: enhanced fatty acid uptake, increased de novo lipid synthesis, decreased fatty acid oxidation, and reduced lipid efflux (Angrish et al., 2016). These pathways, when disrupted, intensify the storage of fat within hepatocytes and trigger chronic inflammation, with elevated levels of pro-inflammatory cytokines like TNF-α and interleukins exacerbating liver damage in a self-perpetuating cycle (Bessone, Razori, & Roma, 2019). Consistent with this mechanistic context, empirical data from HepaRG models within the OBERON project show that DEHP exposure significantly increases lipid-droplet accumulation in both 2D and 3D cultures, supporting its contribution to hepatocellular triglyceride storage and early steatosis (Bernal et al., 2024)

Recent studies suggest that environmental factors, including endocrine disruptors like DEHP, can accelerate steatosis by further disrupting lipid pathways and increasing oxidative stress. Extracellular vesicles (EVs) have emerged as potential biomarkers in this context, carrying molecular signals that reflect cellular stress and inflammation in liver cells exposed to DEHP. These EVs not only serve as indicators of liver health but may also propagate inflammatory signals to other cells, amplifying liver damage and promoting the progression toward metabolic dysfunction-associated steatotic liver disease (MASLD) (Merret, Sparfel, Lavau, Lagadic-Gossmann, & Martin-Chouly, 2024).

KE 14: Hepatic Insulin Resistance

Hepatic insulin resistance, defined as the liver's reduced ability to respond to insulin, leads to increased glucose production and release, thereby contributing to systemic insulin resistance and hyperglycemia (Andrade & Andrade, 2024; Rector et al., 2013). Results from HepaRG cells exposed to DEHP reveal critical disruptions in pathways contributing to this condition. Specifically, DEHP exposure resulted in upregulation of PPARγ, a lipid-regulating nuclear receptor, and increased expression of gluconeogenic enzymes like phosphoenolpyruvate carboxykinase (PCK1) and glucose-6-phosphatase (G6PC), which drives excess glucose production typical of hepatic insulin resistance. Additionally, DEHP inhibited serine phosphorylation of insulin receptor substrate-1 (IRS-1), further impairing insulin signaling. Alterations in serine levels across all DEHP concentrations substantiate these findings, suggesting a direct mechanism through which DEHP contributes to hepatic insulin resistance.

KE 15: NAFLD (Non-Alcoholic Fatty Liver Disease)

NAFLD is characterized by excessive fat accumulation within liver cells, ranging from simple steatosis to non-alcoholic steatohepatitis (NASH), fibrosis, and cirrhosis. This condition is closely associated with metabolic syndromes such as insulin resistance and obesity, where increased hepatic lipid content correlates with diminished systemic insulin sensitivity (Bellentani, 2017; Gastaldelli et al., 2007). Hepatic fat buildup originates from both enhanced uptake of free fatty acids (FFAs) from the bloodstream and increased de novo lipogenesis, compounded by mitochondrial inefficiencies that reduce lipid clearance (A. Lockman & J. Nyirenda, 2010).

Mitochondrial dysfunction, as supported by systems biology models and recent studies, plays a critical role in NAFLD progression, where impaired oxidative phosphorylation and reduced ATP synthesis lead to lipid peroxidation, oxidative stress, and inflammatory responses in hepatocytes (Ramanathan, Ali, & Ibdah, 2022). Additionally, environmental toxins such as di-(2-ethylhexyl) phthalate (DEHP) have been shown to exacerbate NAFLD. DEHP exposure promotes hepatic steatosis through activation of the JAK2/STAT5 pathway, oxidative stress, and inflammation, leading to further metabolic disruption (H. Chen et al., 2016).

Emerging evidence highlights the association between DEHP exposure and NAFLD in populations with high metabolic risk profiles. Studies show that DEHP can influence NAFLD progression through pathways involving BMI and waist circumference, with research suggesting that metabolic disruptions due to DEHP contribute to greater body fat accumulation, which in turn exacerbates NAFLD progression (Y. He, Zou, Hong, & Feng, 2023). Further meta-analytic reviews indicate that endocrine-disrupting chemicals, including DEHP, are significantly associated with the risk of NAFLD development, reinforcing DEHP’s role in lipid dysregulation and metabolic disease (K. Pan, Xu, Xu, Wang, & Yu, 2024).

KE 16: Obesity

Obesity is a systemic adverse outcome characterized by excessive adipose tissue accumulation and broad metabolic dysregulation. It is closely associated with insulin resistance, chronic low-grade inflammation, and altered lipid and glucose homeostasis, which collectively elevate the risk of type 2 diabetes, cardiovascular disease, and other metabolic disorders ((Meijnikman et al., 2022).. Although traditionally attributed to an imbalance between caloric intake and energy expenditure, obesity is increasingly understood as a condition influenced by environmental exposures, particularly endocrine-disrupting chemicals (Heindel et al., 2022; Lustig et al., 2022; Yazıcı & Sezer, 2017).

Accumulating evidence indicates that DEHP acts as an obesogen by disrupting key metabolic regulatory pathways. Chronic DEHP exposure has been associated with altered adipocyte differentiation, impaired insulin signaling, and activation of lipogenic pathways, ultimately promoting excess lipid storage and adipocyte hypertrophy (Yazıcı & Sezer, 2017; Hong Zhang et al., 2022). . DEHP has also been shown to interfere with pancreatic β-cell function and hepatic lipid handling, further contributing to systemic metabolic imbalance (Heindel et al., 2022). Additionally, DEHP-induced perturbations in the gut–liver axis and gut microbiota composition exacerbate inflammation and dysregulated lipid metabolism, increasing susceptibility to obesity (Lustig et al., 2022). For example, studies show that DEHP can alter gut microbiota composition, leading to lipid metabolism disruption and obesity (Pei et al., 2020; Sen et al., 2024; Yifan Zhang et al., 2023). DEHP's modulation of the hypothalamic-pituitary axis also affects appetite regulation, further contributing to obesity and metabolic disorders (Kozlova et al., 2022; La Merrill et al., 2025; Peng, 2022; Wu et al., 2023).

Together, these findings position obesity as a downstream adverse outcome arising from multiple interconnected metabolic disturbances triggered by DEHP exposure. While the mechanistic contributors (e.g., insulin resistance, lipid metabolism disruption, sphingolipid imbalance) are detailed in the relevant upstream KEs and KERs, KE16 represents the integrated whole-organism consequence: increased adiposity and impaired metabolic function resulting from cumulative disruption across endocrine, hepatic, adipose, and gut-derived pathways.

KER 18: Hepatic insulin resistance Leads to NAFLD

Hepatic insulin resistance promotes NAFLD by directly enhancing lipid deposition within the liver, as the failure to suppress gluconeogenesis and de novo lipogenesis leads to abnormal fat accumulation. Insulin resistance reduces the liver's response to insulin signaling, thereby increasing glucose production and upregulating lipogenic transcription factors, such as SREBP-1c and PPAR-γ, which drive the synthesis of fatty acids and triglycerides within hepatocytes (A. Lockman & J. Nyirenda, 2010; Gastaldelli et al., 2007). In insulin-resistant states, the hepatic shift toward excessive lipogenesis and reduced fatty acid oxidation culminates in steatosis, which progressively disrupts liver function and initiates inflammation, fibrosis, and further metabolic dysregulation (Browning et al., 2004; Samuel & Shulman, 2016).

Experimental data support this progression in DEHP-exposed models, where disruptions in gluconeogenesis and lipid metabolism align with enhanced fat accumulation, linking DEHP exposure to the worsening of NAFLD through impaired insulin and lipid homeostasis (H. Chen et al., 2016; Y. He et al., 2023). This sequence underscores how insulin resistance in hepatic cells not only impacts glucose homeostasis but actively drives the liver’s susceptibility to metabolic liver disease.

**Weight-of-Evidence (KER18)**

- **Evidence and Plausibility:** The mechanistic link between hepatic insulin resistance and NAFLD is well established. Insulin-resistant hepatocytes increase gluconeogenesis and upregulate lipogenic pathways (SREBP-1c, PPARγ), creating a metabolic shift toward triglyceride accumulation (A. Lockman & J. Nyirenda, 2010; Browning et al., 2004; Gastaldelli et al., 2007; Samuel & Shulman, 2016)). The mechanisms described above for DEHP—altered gluconeogenesis, lipid deposition, and disrupted IRS/PI3K/Akt signaling—match this canonical progression, reinforcing the biological plausibility(H. Chen et al., 2016; L. He et al., 2023).
- **Temporal and Dose–Response Concordance:** Studies consistently show that hepatic insulin resistance develops before or alongside increased de novo lipogenesis and hepatic fat accumulation(Browning et al., 2004; Gastaldelli et al., 2007). DEHP studies report parallel temporal patterns, where metabolic disruption precedes or co-occurs with fat accumulation(H. Chen et al., 2016).
- **Essentiality:** Interventions that restore insulin signaling reduce hepatic lipid accumulation and blunt NAFLD progression, demonstrating strong essentiality for this KER (Gastaldelli et al., 2007; Samuel & Shulman, 2016).

KER 19: Steatosis Leads to NAFLD

The progression from hepatic steatosis to non-alcoholic fatty liver disease (NAFLD) occurs when fat accumulation in the liver cells is coupled with inflammatory and fibrotic responses. Initial fat accumulation, largely driven by lipid dysregulation and impaired fatty acid oxidation, sensitizes the liver to further damage and increases susceptibility to oxidative stress and inflammation, creating a conducive environment for NAFLD development (Geisler & Renquist, 2017; Q. Liu, Bengmark, & Qu, 2010). Studies have shown that, over time, the presence of triglycerides within hepatocytes activates stress pathways, which in turn initiate the production of pro-inflammatory cytokines and fibrotic markers, progressively advancing liver pathology from simple steatosis to NAFLD (Gluchowski et al., 2019; Régnier, Polizzi, Guillou, & Loiseau, 2019).

The transition to NAFLD is also modulated by environmental toxicants, such as DEHP, which has been shown to activate lipid accumulation pathways, including LXR/SREBP-1c, PPARγ, and NF-κB, further exacerbating steatosis and driving the progression to NAFLD (Bernal et al., 2024; Y.-Q. Huang et al., n.d.; Liang et al., 2024). This pathway activation not only increases lipid synthesis but also induces inflammation and fibrosis, reinforcing the link between DEHP exposure, steatosis, and NAFLD.

**Weight-of-Evidence (KER19)**

- **Evidence and Plausibility**: The progression from hepatic steatosis to NAFLD is one of the best-supported transitions in liver pathophysiology. Steatosis increases susceptibility to oxidative stress, inflammation, and fibrogenesis—the processes that define NAFLD and NASH(Geisler & Renquist, 2017; Gluchowski et al., 2019; Q. Liu et al., 2010; Régnier et al., 2019). Our findings and the DEHP literature align with this: DEHP consistently induces lipid accumulation, inflammatory signaling, and mitochondrial stress (Bernal et al., 2024; H. Chen et al., 2016; Y. Q. Huang et al., 2022; Liang et al., 2024).
- **Temporal Concordance:** Steatosis always precedes NAFLD by definition. Experimental and animal studies, including DEHP exposure. show that lipid accumulation occurs first, followed by inflammatory and fibrotic markers(Gluchowski et al., 2019; Régnier et al., 2019). The transition from simple steatosis to NASH often involves multiple hits, with steatosis being the initial hit (Bessone et al., 2019; Browning et al., 2004; Samala, Tersey, Chalasani, Anderson, & Mirmira, 2017)

**Essentiality:** Steatosis is a required precursor for NAFLD. Models that block triglyceride accumulation also block NAFLD progression (e.g., DGAT knockout models), supporting very high essentiality (Greuter, Malhi, Gores, & Shah, 2017; Mashek, 2021; Thayer et al., 2020)

### References

A. Lockman, K., & J. Nyirenda, M. (2010). Interrelationships between Hepatic Fat and Insulin Resistance in Non- Alcoholic Fatty Liver Disease. *Current Diabetes Reviews*, 6(5), 341–347. Retrieved from https://doi.org/10.2174/157339910793360879

Abdelmalek, M. F., Lazo, M., Horska, A., Bonekamp, S., Lipkin, E. W., Balasubramanyam, A., … Clark, J. M. (2012). Higher Dietary Fructose Is Associated with Impaired Hepatic ATP Homeostasis in Obese Individuals with Type 2 Diabetes. *Hepatology (Baltimore, Md.)*, 56(3), 952. Retrieved 8 January 2026 from https://doi.org/10.1002/HEP.25741

Adeva-Andany, M. M., Carneiro-Freire, N., Seco-Filgueira, M., Fernández-Fernández, C., & Mouriño-Bayolo, D. (2019). Mitochondrial β-oxidation of saturated fatty acids in humans. *Mitochondrion*, 46, 73–90. Retrieved from https://doi.org/10.1016/j.mito.2018.02.009

Ajaz, S., McPhail, M. J., Gnudi, L., Trovato, F. M., Mujib, S., Napoli, S., … Agarwal, K. (2021). Mitochondrial dysfunction as a mechanistic biomarker in patients with non-alcoholic fatty liver disease (NAFLD). *Mitochondrion*, 57, 119–130. Retrieved 14 November 2025 from https://doi.org/10.1016/J.MITO.2020.12.010

Al-Abdulla, R., Ferrero, H., Soriano, S., Boronat-Belda, T., & Alonso-Magdalena, P. (2022). Screening of Relevant Metabolism-Disrupting Chemicals on Pancreatic β-Cells: Evaluation of Murine and Human In Vitro Models. *International Journal of Molecular Sciences*, 23(8), 1–29. Retrieved 8 January 2026 from https://doi.org/10.3390/ijms23084182

Al-Muzafar, H. M., & Amin, K. A. (2018). Thiazolidinedione induces a therapeutic effect on hepatosteatosis by regulating stearoyl-CoA desaturase-1, lipase activity, leptin and resistin. *Experimental and Therapeutic Medicine*, 16(4), 2938–2948. Retrieved from https://doi.org/10.3892/etm.2018.6563

Ali-Berrada, S., Guitton, J., Tan-Chen, S., Gyulkhandanyan, A., Hajduch, E., & Le Stunff, H. (2023). Circulating Sphingolipids and Glucose Homeostasis: An Update. *International Journal of Molecular Sciences*, 24(16). Retrieved 8 January 2026 from https://doi.org/10.3390/IJMS241612720

Alonso-Magdalena, P., Tudurí, E., Marroquí, L., Quesada, I., Sargis, R. M., & Nadal, A. (2018). *Toxic effects of common environmental pollutants in pancreatic β-cells and the onset of diabetes mellitus*. *Encyclopedia of Endocrine Diseases* (2nd ed.). Elsevier Inc. Retrieved from https://doi.org/10.1016/B978-0-12-801238-3.64325-8

Alves-Bezerra, M., & Cohen, D. E. (2017). Triglyceride metabolism in the liver. *Comprehensive Physiology*, 8(1), 1–22. Retrieved from https://doi.org/10.1002/cphy.c170012

Andrade, L. J. O., & Andrade, J. C. N. C. (2024). “in-Situ Insulin Resistance – Localized Typ E 2 Diabetes Mellitus or Type 6 Diabetes Mellitus?”: a Scoping Review. *Endocrinologia & Diabetes Clínica e Experimental*, 21(2), 120–144. Retrieved from https://doi.org/10.29327/2413063.21.2-7

Angerer, H., Radermacher, M., Mankowska, M., Steger, M., Zwicker, K., Heide, H., … Zickermann, V. (2014). The LYR protein subunit NB4M/NDUFA6 of mitochondrial complex i anchors an acyl carrier protein and is essential for catalytic activity. *Proceedings of the National Academy of Sciences of the United States of America*, 111(14), 5207–5212. Retrieved from https://doi.org/10.1073/pnas.1322438111

Angrish, M. M., Kaiser, J. P., McQueen, C. A., & Chorley, B. N. (2016). Tipping the balance: Hepatotoxicity and the 4 apical key events of hepatic steatosis. *Toxicological Sciences*, 150(2), 261–268. Retrieved from https://doi.org/10.1093/toxsci/kfw018

Apostolova, N., & Victor, V. M. (2015). Molecular strategies for targeting antioxidants to mitochondria: therapeutic implications. *Antioxidants & Redox Signaling*, 22(8), 686–729. Retrieved 7 January 2026 from https://doi.org/10.1089/ARS.2014.5952

Arnold, P. K., & Finley, L. W. S. (2023). Regulation and function of the mammalian tricarboxylic acid cycle. *Journal of Biological Chemistry*, 299(2), 102838. Retrieved 7 January 2026 from https://doi.org/10.1016/J.JBC.2022.102838

Asano, M., Kajita, K., Fuwa, M., Kajita, T., Mori, I., Akahoshi, N., … Morita, H. (2023). Opposing Roles of Sphingosine 1-Phosphate Receptors 1 and 2 in Fat Deposition and Glucose Tolerance in Obese Male Mice. *Endocrinology*, 164(3), 1–13. Retrieved 23 October 2024 from https://doi.org/10.1210/ENDOCR/BQAD019

Ashrap, P., Aung, M. T., Watkins, D. J., Mukherjee, B., Rosario-Pabón, Z., Vélez-Vega, C. M., … Meeker, J. D. (2021). Maternal Urinary Phthalate Metabolites are Associated with Lipidomic Signatures Among Pregnant Women in Puerto Rico. *MedRxiv*, 2021.07.19.21260700. Retrieved 7 January 2026 from https://doi.org/10.1101/2021.07.19.21260700

Ashrap, P., Aung, M. T., Watkins, D. J., Mukherjee, B., Rosario-Pabón, Z., Vélez-Vega, C. M., … Meeker, J. D. (2022). Maternal Urinary Phthalate Metabolites are Associated with Lipidomic Signatures Among Pregnant Women in Puerto Rico. *Journal of Exposure Science & Environmental Epidemiology*, 32(3), 384. Retrieved 14 November 2025 from https://doi.org/10.1038/S41370-022-00410-3

Begriche, K., Massart, J., Robin, M. A., Bonnet, F., & Fromenty, B. (2013). Mitochondrial adaptations and dysfunctions in nonalcoholic fatty liver disease. *Hepatology*, 58(4), 1497–1507. Retrieved from https://doi.org/10.1002/hep.26226

Bellentani, S. (2017). The epidemiology of non-alcoholic fatty liver disease. *Liver International*, 37, 81–84. Retrieved from https://doi.org/10.1111/liv.13299

Bernal, K., Touma, C., Erradhouani, C., Boronat-Belda, T., Gaillard, L., Al Kassir, S., … Coumoul, X. (2022). Combinatorial pathway disruption is a powerful approach to delineate metabolic impacts of endocrine disruptors. *FEBS Letters*, 596(24), 3107–3123. Retrieved from https://doi.org/10.1002/1873-3468.14465

Bernal, K., Touma, C., Le-Grand, B., Rose, S., Degerli, S., Genêt, V., … Blanc, E. B. (2024). Assessment of endocrine disruptor impacts on lipid metabolism in a fatty acid-supplemented HepaRG human hepatic cell line. *Chemosphere*, 349(October 2023). Retrieved from https://doi.org/10.1016/j.chemosphere.2023.140883

Bessone, F., Razori, M. V., & Roma, M. G. (2019). Molecular pathways of nonalcoholic fatty liver disease development and progression. *Cellular and Molecular Life Sciences*, 76(1), 99–128. Retrieved from https://doi.org/10.1007/s00018-018-2947-0

Biddinger, S. B., & Kahn, C. R. (2006). From mice to men: Insights into the insulin resistance syndromes. *Annual Review of Physiology*, 68(Volume 68, 2006), 123–158. Retrieved 24 October 2024 from https://doi.org/10.1146/ANNUREV.PHYSIOL.68.040104.124723/CITE/REFWORKS

Bjørndal, B., Alterås, E. K., Lindquist, C., Svardal, A., Skorve, J., & Berge, R. K. (2018). Associations between fatty acid oxidation, hepatic mitochondrial function, and plasma acylcarnitine levels in mice. *Nutrition and Metabolism*, 15(1), 1–13. Retrieved 28 August 2025 from https://doi.org/10.1186/S12986-018-0241-7/FIGURES/5

Boura-Halfon, S., & Zick, Y. (2009). Phosphorylation of IRS proteins, insulin action, and insulin resistance. *Https://Doi.Org/10.1152/Ajpendo.90437.2008*, 296(4), 581–591. Retrieved 14 November 2025 from https://doi.org/10.1152/AJPENDO.90437.2008

Brillo, V., Chieregato, L., Leanza, L., Muccioli, S., Costa, R., Brillo, V., … Costa, R. (2021). Mitochondrial Dynamics, ROS, and Cell Signaling: A Blended Overview. *Life 2021, Vol. 11,* 11(4). Retrieved 8 January 2026 from https://doi.org/10.3390/LIFE11040332

Browning, J. D., Szczepaniak, L. S., Dobbins, R., Nuremberg, P., Horton, J. D., Cohen, J. C., … Hobbs, H. H. (2004). Prevalence of Hepatic Steatosis in An Urban Population in the United States: Impact of Ethnicity. *Hepatology*, 40(6). Retrieved from https://journals.lww.com/hep/fulltext/2004/12000/prevalence_of_hepatic_steatosis_in_an_urban.22.aspx

Buckingham, R. E. (2005). Thiazolidinediones: Pleiotropic drugs with potent anti-inflammatory properties for tissue protection. *Hepatology Research*, 33(2), 167–170. Retrieved from https://doi.org/10.1016/j.hepres.2005.09.027

Buerger, A. N., Schmidt, J., Chase, A., Paxaio, C., Patel, T. N., Brumback, B. A., … Bisesi, J. H. (2019). Examining the responses of the zebrafish (Danio rerio) gastrointestinal system to the suspected obesogen diethylhexyl phthalate. *Environmental Pollution*, 245, 1086–1094. Retrieved from https://doi.org/10.1016/J.ENVPOL.2018.11.032

Chai, S. C., & Chen, T. (2022). Nuclear Receptors. In *Comprehensive Pharmacology* (Vol. 1, pp. 151–164). John Wiley & Sons, Ltd. Retrieved 29 July 2024 from https://doi.org/10.1016/B978-0-12-820472-6.00113-4

Chakrabarty, S., Bui, Q., Badeanlou, L., Hester, K., Chun, J., Ruf, W., … Samad, F. (2022). S1P/S1PR3 signalling axis protects against obesity-induced metabolic dysfunction. *Adipocyte*, 11(1), 69–83. Retrieved from https://doi.org/10.1080/21623945.2021.2021700

Chandrasekaran, P., & Weiskirchen, R. (2024). Cellular and Molecular Mechanisms of Insulin Resistance. *Current Tissue Microenvironment Reports 2024 5:3*, 5(3), 79–90. Retrieved 8 January 2026 from https://doi.org/10.1007/S43152-024-00056-3

Chen, H., Zhang, W., Rui, B. bei, Yang, S. min, Xu, W. ping, & Wei, W. (2016). Di(2-ethylhexyl) phthalate exacerbates non-alcoholic fatty liver in rats and its potential mechanisms. *Environmental Toxicology and Pharmacology*, 42, 38–44. Retrieved from https://doi.org/10.1016/j.etap.2015.12.016

Chen, W., Lu, H., Yang, J., Xiang, H., & Peng, H. (2016). Sphingosine 1-phosphate in metabolic syndrome (Review). *International Journal of Molecular Medicine*, 38(4), 1030–1038. Retrieved from https://doi.org/10.3892/ijmm.2016.2731

Chen, Y. H., Wu, Y. J., Chen, W. C., Lee, T. S., Tsou, T. C., Chang, H. C., … Chen, S. L. (2020). MEHP interferes with mitochondrial functions and homeostasis in skeletal muscle cells. *Bioscience Reports*, 40(4). Retrieved 7 January 2026 from https://doi.org/10.1042/BSR20194404

Cho, H., Seol, Y., Baik, S., Sung, B., Ryu, C. S., & Kim, Y. J. (2022). Mono(2-ethylhexyl) phthalate modulates lipid accumulation and reproductive signaling in Daphnia magna. *Environmental Science and Pollution Research International*, 29(37), 55639–55650. Retrieved 8 January 2026 from https://doi.org/10.1007/S11356-022-19701-1

Cichoz-Lach, H., & Michalak, A. (2014). Oxidative stress as a crucial factor in liver diseases. *World Journal of Gastroenterology*, 20(25), 8082–8091. Retrieved 7 August 2024 from https://doi.org/10.3748/WJG.V20.I25.8082

Daussin, F. N., Boulanger, E., & Lancel, S. (2021). From mitochondria to sarcopenia: Role of inflammaging and RAGE-ligand axis implication. *Experimental Gerontology*, 146. Retrieved 7 January 2026 from https://doi.org/10.1016/j.exger.2021.111247

Dawson, M. I., & Xia, Z. (2012). The retinoid X receptors and their ligands. *Biochimica et Biophysica Acta - Molecular and Cell Biology of Lipids*, 1821(1), 21–56. Retrieved 7 January 2026 from https://doi.org/10.1016/j.bbalip.2011.09.014

DeFronzo, R. A. (2010). Insulin resistance, lipotoxicity, type 2 diabetes and atherosclerosis: The missing links. The Claude Bernard Lecture 2009. *Diabetologia*, 53(7), 1270–1287. Retrieved from https://doi.org/10.1007/s00125-010-1684-1

Deng, K., Pan, M., Liu, J., Yang, M., Gu, Z., Zhang, Y., … Mai, K. (2018). Chronic stress of high dietary carbohydrate level causes inflammation and influences glucose transport through SOCS3 in Japanese flounder Paralichthys olivaceus. *Scientific Reports 2018 8:1*, 8(1), 7415-. Retrieved 14 November 2025 from https://doi.org/10.1038/s41598-018-25412-w

Ding, Y., Xu, T., Mao, G., Chen, Y., Qiu, X., Yang, L., … Wu, X. (2021). Di-(2-ethylhexyl) phthalate-induced hepatotoxicity exacerbated type 2 diabetes mellitus (T2DM) in female pubertal T2DM mice. *Food and Chemical Toxicology*, 149, 112003. Retrieved from https://doi.org/10.1016/J.FCT.2021.112003

DONG, X. W., WU, W. D., YAO, S. Q., ZHANG, Y. B., WANG, C., WU, H. Y., & NA, X. L. (2021, November 20). Long-term Di-(2-ethylhexyl) phthalate Exposure Disturbs the Lipid Metabolism Profiles and Hepatic Enzymes in Male Rats: A UPLC-MS-based Serum Metabolomics Analysis. *Biomedical and Environmental Sciences*. Biomedical and Environmental Sciences. Retrieved 7 January 2026 from https://doi.org/10.3967/bes2021.127

Ebanks, B., & Chakrabarti, L. (2022). Mitochondrial ATP Synthase is a Target of Oxidative Stress in Neurodegenerative Diseases. *Frontiers in Molecular Biosciences*, 9, 854321. Retrieved 14 November 2025 from https://doi.org/10.3389/FMOLB.2022.854321/BIBTEX

Esch, B. M., Limar, S., Bogdanowski, A., Gournas, C., More, T., Sundag, C., … Fröhlich, F. (2020). Uptake of exogenous serine is important to maintain sphingolipid homeostasis in Saccharomyces cerevisiae. *PLoS Genetics*, 16(8). Retrieved from https://doi.org/10.1371/JOURNAL.PGEN.1008745

Fang, Haiqin, Fang, W., Cao, H., Luo, S., Cong, J., Liu, S., … Jia, X. (2019). Di-(2-ethylhexyl)-phthalate induces apoptosis via the PPARγ/PTEN/AKT pathway in differentiated human embryonic stem cells. *Food and Chemical Toxicology*, 131, 110552. Retrieved from https://doi.org/10.1016/j.fct.2019.05.060

Fang, Hongjuan, Feng, Q., Shi, Y., Zhou, J., Wang, Q., & Zhong, L. (2020). Hepatic insulin resistance induced by mitochondrial oxidative stress can be ameliorated by sphingosine 1-phosphate. *Molecular and Cellular Endocrinology*, 501, 110660. Retrieved from https://doi.org/10.1016/J.MCE.2019.110660

Fang, Z., Pyne, S., & Pyne, N. J. (2019). Ceramide and sphingosine 1-phosphate in adipose dysfunction. *Progress in Lipid Research*, 74, 145–159. Retrieved 8 January 2026 from https://doi.org/10.1016/j.plipres.2019.04.001

Fayyaz, S., Henkel, J., Japtok, L., Krämer, S., Damm, G., Seehofer, D., … Kleuser, B. (2014). Involvement of sphingosine 1-phosphate in palmitate-induced insulin resistance of hepatocytes via the S1P2 receptor subtype. *Diabetologia*, 57(2), 373–382. Retrieved from https://doi.org/10.1007/s00125-013-3123-6

Fayyaz, S., Japtok, L., & Kleuser, B. (2014). Divergent role of sphingosine 1-phosphate on insulin resistance. *Cellular Physiology and Biochemistry*, 34(1), 134–147. Retrieved from https://doi.org/10.1159/000362990

Feige, J. N., Gelman, L., Rossi, D., Zoete, V., Métivier, R., Tudor, C., … Desvergne, B. (2007). The Endocrine Disruptor Monoethyl-hexyl-phthalate Is a Selective Peroxisome Proliferator-activated Receptor γ Modulator That Promotes Adipogenesis. *Journal of Biological Chemistry*, 282(26), 19152–19166. Retrieved from https://doi.org/10.1074/JBC.M702724200

Feng, J., Lu, S., Ou, B., Liu, Q., Dai, J., Ji, C., … Ma, Y. (2020). The Role of JNk Signaling Pathway in Obesity-Driven Insulin Resistance. *Diabetes, Metabolic Syndrome and Obesity: Targets and Therapy*, 13, 1399. Retrieved 14 November 2025 from https://doi.org/10.2147/DMSO.S236127

Forman, H. J., Zhang, H., & Rinna, A. (2009). Glutathione: Overview of its protective roles, measurement, and biosynthesis. *Molecular Aspects of Medicine*, 30(1–2), 1–12. Retrieved 8 January 2026 from https://doi.org/10.1016/j.mam.2008.08.006

Galdieri, L., & Vancura, A. (2012). Acetyl-CoA Carboxylase Regulates Global Histone Acetylation. *Journal of Biological Chemistry*, 287(28), 23865–23876. Retrieved 14 November 2025 from https://doi.org/10.1074/JBC.M112.380519

Galili, O., Versari, D., Sattler, K. J., Olson, M. L., Mannheim, D., McConnell, J. P., … Lerman, A. (2007). Early experimental obesity is associated with coronary endothelial dysfunction and oxidative stress. *American Journal of Physiology - Heart and Circulatory Physiology*, 292(2), 904–911. Retrieved from https://doi.org/10.1152/ajpheart.00628.2006

García-Ruiz, I., Solís-Muñoz, P., Fernández-Moreira, D., Grau, M., Colina, F., Muñoz-Yagüe, T., & Solís-Herruzo, J. A. (2014). High-fat diet decreases activity of the oxidative phosphorylation complexes and causes nonalcoholic steatohepatitis in mice. *DMM Disease Models and Mechanisms*, 7(11), 1287–1296. Retrieved from https://doi.org/10.1242/dmm.016766

Gastaldelli, A., Cusi, K., Pettiti, M., Hardies, J., Miyazaki, Y., Berria, R., … DeFronzo, R. A. (2007). Relationship Between Hepatic/Visceral Fat and Hepatic Insulin Resistance in Nondiabetic and Type 2 Diabetic Subjects. *Gastroenterology*, 133(2), 496–506. Retrieved from https://doi.org/10.1053/j.gastro.2007.04.068

Geisler, C. E., & Renquist, B. J. (2017). Hepatic lipid accumulation: Cause and consequence of dysregulated glucoregulatory hormones. *Journal of Endocrinology*, 234(1), R1–R21. Retrieved from https://doi.org/10.1530/JOE-16-0513

Gier, B., Krippeit-Drews, P., Sheiko, T., Aguilar-Bryan, L., Bryan, J., Düfer, M., & Drews, G. (2009). Suppression of KATP channel activity protects murine pancreatic β cells against oxidative stress. *The Journal of Clinical Investigation*, 119(11), 3246–3256. Retrieved 7 January 2026 from https://doi.org/10.1172/JCI38817

Giorgi, C., Marchi, S., Simoes, I. C. M., Ren, Z., Morciano, G., Perrone, M., … Wieckowski, M. R. (2018). Mitochondria and Reactive Oxygen Species in Aging and Age-Related Diseases, 340, 209–344. Retrieved 14 November 2025 from https://pubmed.ncbi.nlm.nih.gov/30072092/

Gluchowski, N. L., Gabriel, K. R., Chitraju, C., Bronson, R. T., Mejhert, N., Boland, S., … Walther, T. C. (2019). Hepatocyte Deletion of Triglyceride-Synthesis Enzyme Acyl CoA: Diacylglycerol Acyltransferase 2 Reduces Steatosis Without Increasing Inflammation or Fibrosis in Mice. *Hepatology*, 70(6), 1972–1985. Retrieved from https://doi.org/10.1002/hep.30765

Goyal, M., Tiwari, S., Arora, J., Cooper, B., Subramanian, R., & Mukherjee, T. (2025). Metabolic coupling of ROS generation and antioxidant synthesis by the GABA shunt pathway in myeloid-like blood progenitor cells of Drosophila. *PLOS Genetics*, 21(9), e1011602. Retrieved 8 January 2026 from https://doi.org/10.1371/JOURNAL.PGEN.1011602

Grattagliano, I., Montezinho, L. P., Oliveira, P. J., Frühbeck, G., Gómez-Ambrosi, J., Montecucco, F., … Portincasa, P. (2019). Targeting mitochondria to oppose the progression of nonalcoholic fatty liver disease. *Biochemical Pharmacology*, 160, 34–45. Retrieved 8 January 2026 from https://doi.org/10.1016/j.bcp.2018.11.020

Green, C. D., Maceyka, M., Cowart, L. A., & Spiegel, S. (2021). Sphingolipids in metabolic disease: The good, the bad, and the unknown. *Cell Metabolism*. Retrieved 28 July 2023 from https://doi.org/10.1016/j.cmet.2021.06.006

Greuter, T., Malhi, H., Gores, G. J., & Shah, V. H. (2017). Therapeutic opportunities for alcoholic steatohepatitis and nonalcoholic steatohepatitis: exploiting similarities and differences in pathogenesis. *JCI Insight*, 2(17). Retrieved 14 November 2025 from https://doi.org/10.1172/JCI.INSIGHT.95354

Gu, T., Duan, M., Zhang, R., Zeng, T., Xu, W., Feng, W., … Lu, L. (2022). Probiotic Fermented Feed Alleviates Liver Fat Deposition in Shaoxing Ducks via Modulating Gut Microbiota. *Frontiers in Microbiology*, 13, 928670. Retrieved 7 January 2026 from https://doi.org/10.3389/FMICB.2022.928670/BIBTEX

Gual, P., Grémeaux, T., Gonzalez, T., Le Marchand-Brustel, Y., & Tanti, J. F. (2003). MAP kinases and mTOR mediate insulin-induced phosphorylation of Insulin Receptor Substrate-1 on serine residues 307, 612 and 632. *Diabetologia*, 46(11), 1532–1542. Retrieved 9 August 2024 from https://doi.org/10.1007/S00125-003-1223-4/FIGURES/8

Guitton, J., Bandet, C. L., Mariko, M. L., Tan-Chen, S., Bourron, O., Benomar, Y., … Le Stunff, H. (2020). Sphingosine-1-Phosphate Metabolism in the Regulation of Obesity/Type 2 Diabetes. *Cells*, 9(7). Retrieved 14 November 2025 from https://doi.org/10.3390/cells9071682

Hait, N. C., & Maiti, A. (2017). The Role of Sphingosine-1-Phosphate and Ceramide-1-Phosphate in Inflammation and Cancer. *Mediators of Inflammation*, 2017. Retrieved from https://doi.org/10.1155/2017/4806541

Hajduch, E., Lachkar, F., Ferré, P., & Foufelle, F. (2021). Roles of ceramides in non-alcoholic fatty liver disease. *Journal of Clinical Medicine*, 10(4), 1–19. Retrieved 28 August 2025 from https://doi.org/10.3390/jcm10040792

Hala, D., Petersen, L. H., Huggett, D. B., Puchowicz, M. A., Brunengraber, H., Zhang, G.-F., … Zhang, G.-F. (2021). Overcompensation of CoA Trapping by Di(2-ethylhexyl) Phthalate (DEHP) Metabolites in Livers of Wistar Rats. *International Journal of Molecular Sciences 2021, Vol. 22,* 22(24). Retrieved 14 November 2025 from https://doi.org/10.3390/IJMS222413489

Handzlik, M. K., & Metallo, C. M. (2023). Sources and Sinks of Serine in Nutrition, Health, and Disease. *Annual Review of Nutrition*, 43(Volume 43, 2023), 123–151. Retrieved 14 November 2025 from https://doi.org/10.1146/ANNUREV-NUTR-061021-022648/CITE/REFWORKS

Hansen, J. S., De Maré, S., Jones, H. A., Göransson, O., & Lindkvist-Petersson, K. (2017). Visualization of lipid directed dynamics of perilipin 1 in human primary adipocytes. *Scientific Reports*, 7(1), 15011. Retrieved from https://doi.org/10.1038/s41598-017-15059-4

Hao, C., Cheng, X., Xia, H., & Ma, X. (2012). The endocrine disruptor mono-(2-ethylhexyl)phthalate promotes adipocyte differentiation and induces obesity in mice. *Bioscience Reports*, 32(6), 619–629. Retrieved 7 January 2026 from https://doi.org/10.1042/BSR20120042

Harrison, P. J., Dunn, T., & Campopiano, D. J. (2018). Sphingolipid biosynthesis in man and microbes. *Natural Product Reports*, 35(9), 921–954. Retrieved from https://doi.org/10.1039/c8np00019k

He, L., Ding, Y., Zhou, X., Li, T., & Yin, Y. (2023). Serine signaling governs metabolic homeostasis and health. *Trends in Endocrinology and Metabolism*, 34(6), 361–372. Retrieved 9 August 2024 from https://doi.org/10.1016/j.tem.2023.03.001

He, Y., Zou, J., Hong, T., & Feng, D. (2023). Association between Di-2-ethylhexyl phthalate and nonalcoholic fatty liver disease among US adults: Mediation analysis of body mass index and waist circumference in the NHANES. *Food and Chemical Toxicology*, 179(April), 113968. Retrieved from https://doi.org/10.1016/j.fct.2023.113968

Heindel, J. J., Howard, S., Agay-Shay, K., Arrebola, J. P., Audouze, K., Babin, P. J., … Blumberg, B. (2022). Obesity II: Establishing causal links between chemical exposures and obesity. *Biochemical Pharmacology*, 199(April), 115015. Retrieved from https://doi.org/10.1016/j.bcp.2022.115015

Hibino, M., Maeki, M., Tokeshi, M., Ishitsuka, Y., Harashima, H., & Yamada, Y. (2023). A system that delivers an antioxidant to mitochondria for the treatment of drug-induced liver injury. *Scientific Reports 2023 13:1*, 13(1), 6961-. Retrieved 8 January 2026 from https://doi.org/10.1038/s41598-023-33893-7

Hisano, Y., Inoue, A., Taimatsu, K., Ota, S., Ohga, R., Kotani, H., … Kawahara, A. (2015). Comprehensive analysis of sphingosine-1-phosphate receptor mutants during zebrafish embryogenesis. *Genes to Cells : Devoted to Molecular & Cellular Mechanisms*, 20(8), 647–658. Retrieved from https://doi.org/10.1111/gtc.12259

Holeček, M. (2022). Serine Metabolism in Health and Disease and as a Conditionally Essential Amino Acid. *Nutrients*, 14(9). Retrieved 9 August 2024 from https://doi.org/10.3390/NU14091987

Hsu, J.-W., Nien, C.-Y., Yeh, S.-C., Tsai, F.-Y., Chen, H.-W., Lee, T.-S., … Tsou, T.-C. (2020). Phthalate exposure causes browning-like effects on adipocytes in vitro and in vivo. *Food and Chemical Toxicology*, 142, 111487. Retrieved from https://doi.org/10.1016/j.fct.2020.111487

Huang, Y.-Q., Tang, Y.-X., Qiu, B.-H., Talukder, M., Li, X.-N., & Li, J.-L. (n.d.). Di-2-ethylhexyl phthalate (DEHP) induced lipid metabolism disorder in liver via activating the LXR/SREBP-1c/PPARα/γ and NF-κB signaling pathway. *Food and Chemical Toxicology*, 165, 113119. Retrieved from https://doi.org/10.1016/j.fct.2022.113119

Huang, Y. Q., Tang, Y. X., Qiu, B. H., Talukder, M., Li, X. N., & Li, J. L. (2022). Di-2-ethylhexyl phthalate (DEHP) induced lipid metabolism disorder in liver via activating the LXR/SREBP-1c/PPARα/γ and NF-κB signaling pathway. *Food and Chemical Toxicology*, 165, 113119. Retrieved from https://doi.org/10.1016/J.FCT.2022.113119

Huang, Y., Wu, C., Ye, Y., Zeng, J., Zhu, J., Li, Y., … Liu, J. (2019). The Increase of ROS Caused by the Interference of DEHP with JNK/p38/p53 Pathway as the Reason for Hepatotoxicity. *International Journal of Environmental Research and Public Health*, 16(3). Retrieved 7 January 2026 from https://doi.org/10.3390/IJERPH16030356

Huff, M., Da Silveira, W. A., Carnevali, O., Renaud, L., & Hardiman, G. (2018). Systems Analysis of the Liver Transcriptome in Adult Male Zebrafish Exposed to the Plasticizer (2-Ethylhexyl) Phthalate (DEHP). *Scientific Reports*, 8(1). Retrieved 8 January 2026 from https://doi.org/10.1038/S41598-018-20266-8

Hughey, C. C., Puchalska, P., & Crawford, P. A. (2022). Integrating the contributions of mitochondrial oxidative metabolism to lipotoxicity and inflammation in NAFLD pathogenesis. *Biochimica et Biophysica Acta - Molecular and Cell Biology of Lipids*, 1867(11). Retrieved from https://doi.org/10.1016/j.bbalip.2022.159209

Hurst, C. H., & Waxman, D. J. (2003). Activation of PPARα and PPARγ by environmental phthalate monoesters. *Toxicological Sciences*, 74(2), 297–308. Retrieved 22 December 2023 from https://doi.org/10.1093/toxsci/kfg145

Ide, T., & Origuchi, I. (2020). An Oil Rich in γ-Linolenic Acid Differently Affects Hepatic Fatty Acid Oxidation in Mice and Rats, 43(9), 1382–1392. Retrieved 7 January 2026 from https://doi.org/10.1248/BPB.B20-00322

Iqbal, M., Pumford, N. R., Tang, Z. X., Lassiter, K., Ojano-Dirain, C., Wing, T., … Bottje, W. (2005). Compromised liver mitochondrial function and complex activity in low feed efficient broilers are associated with higher oxidative stress and differential protein expression. *Poultry Science*, 84(6), 933–941. Retrieved 8 January 2026 from https://doi.org/10.1093/ps/84.6.933

Ishii, I., Fukushima, N., Ye, X., & Chun, J. (2004). Lysophospholipid receptors: Signaling and biology. *Annual Review of Biochemistry*, 73(1), 321–354. Retrieved from https://doi.org/10.1146/annurev.biochem.73.011303.073731

Jo, J. Y., Kim, T. H., Jeong, H. Y., Lim, S. M., Kim, H. S., & Im, D. S. (2011). Effect of Di-(2-ethylhexyl)-phthalate on sphingolipid metabolic enzymes in rat liver. *Toxicological Research*, 27(3), 185–190. Retrieved from https://doi.org/10.5487/TR.2011.27.3.185

Jornayvaz, F. R., & Shulman, G. I. (2012). Diacylglycerol activation of protein kinase Cε and hepatic insulin resistance. *Cell Metabolism*, 15(5), 574–584. Retrieved from https://doi.org/10.1016/j.cmet.2012.03.005

Juchnicka, I., Kuźmicki, M., & Szamatowicz, J. (2021). Ceramides and Sphingosino-1-Phosphate in Obesity. *Frontiers in Endocrinology*, 12(May), 1–7. Retrieved 14 November 2025 from https://doi.org/10.3389/fendo.2021.635995

Jung, K. H., Lee, S., Kim, H. S., Kim, J. M., Lee, Y. J., Park, M. S., … Hong, S. S. (2024). Acetyl-CoA synthetase 2 contributes to a better prognosis for liver cancer by switching acetate-glucose metabolism. *Experimental & Molecular Medicine 2024 56:3*, 56(3), 721–733. Retrieved 7 January 2026 from https://doi.org/10.1038/s12276-024-01185-3

Kajita, K., Ishii, I., Mori, I., Asano, M., Fuwa, M., & Morita, H. (2024). Sphingosine 1-Phosphate Regulates Obesity and Glucose Homeostasis. *International Journal of Molecular Sciences*, 1–18.

Kasumov, T., Li, L., Li, M., Gulshan, K., Kirwan, J. P., Liu, X., … McCullough, A. (2015). Ceramide as a mediator of non-alcoholic fatty liver disease and associated atherosclerosis. *PLoS ONE*, 10(5). Retrieved from https://doi.org/10.1371/journal.pone.0126910

Kawano, Y., & Cohen, D. E. (2013). Mechanisms of hepatic triglyceride accumulation in non-alcoholic fatty liver disease. *Journal of Gastroenterology 2013 48:4*, 48(4), 434–441. Retrieved 7 January 2026 from https://doi.org/10.1007/S00535-013-0758-5

Kim, J. H., Park, H. Y., Bae, S., Lim, Y. H., & Hong, Y. C. (2013). Diethylhexyl Phthalates Is Associated with Insulin Resistance via Oxidative Stress in the Elderly: A Panel Study. *PLOS ONE*, 8(8), e71392. Retrieved 8 January 2026 from https://doi.org/10.1371/JOURNAL.PONE.0071392

Kitada, Y., Kajita, K., Taguchi, K., Mori, I., Yamauchi, M., Ikeda, T., … Morita, H. (2016). Blockade of Sphingosine 1-Phosphate Receptor 2 Signaling Attenuates High-Fat Diet-Induced Adipocyte Hypertrophy and Systemic Glucose Intolerance in Mice. *Endocrinology*, 157(5), 1839–1851. Retrieved from https://doi.org/10.1210/en.2015-1768

Kleuser, B. (2018). Divergent Role of Sphingosine 1-Phosphate in Liver Health and Disease. *International Journal of Molecular Sciences*, 19(3). Retrieved from https://doi.org/10.3390/ijms19030722

Klöting, N., Hesselbarth, N., Gericke, M., Kunath, A., Biemann, R., Chakaroun, R., … Blüher, M. (2015). Di-(2-Ethylhexyl)-Phthalate (DEHP) Causes Impaired Adipocyte Function and Alters Serum Metabolites. *PLOS ONE*, 10(12), e0143190. Retrieved 14 November 2025 from https://doi.org/10.1371/JOURNAL.PONE.0143190

Kocbach Bølling, A., Ovrevik, J., Samuelsen, J. T., Holme, J. A., Rakkestad, K. E., Mathisen, G. H., … Becher, R. (2012). Mono-2-ethylhexylphthalate (MEHP) induces TNF-α release and macrophage differentiation through different signalling pathways in RAW264.7 cells. *Toxicology Letters*, 209(1), 43–50. Retrieved from https://doi.org/10.1016/J.TOXLET.2011.11.016

Koliaki, C., & Roden, M. (2013). Hepatic energy metabolism in human diabetes mellitus, obesity and non-alcoholic fatty liver disease. *Molecular and Cellular Endocrinology*, 379(1–2), 35–42. Retrieved from https://doi.org/10.1016/j.mce.2013.06.002

Koliaki, C., Szendroedi, J., Kaul, K., Jelenik, T., Nowotny, P., Jankowiak, F., … Roden, M. (2015). Adaptation of Hepatic Mitochondrial Function in Humans with Non-Alcoholic Fatty Liver Is Lost in Steatohepatitis. *Cell Metabolism*, 21(5), 739–746. Retrieved from https://doi.org/10.1016/j.cmet.2015.04.004

Koves, T. R., Zhang, G. F., Davidson, M. T., Chaves, A. B., Crown, S. B., Johnson, J. M., … Muoio, D. M. (2023). Pyruvate-supported flux through medium-chain ketothiolase promotes mitochondrial lipid tolerance in cardiac and skeletal muscles. *Cell Metabolism*, 35(6), 1038-1056.e8. Retrieved 14 November 2025 from https://doi.org/10.1016/J.CMET.2023.03.016

Kowalczyk, P., Sulejczak, D., Kleczkowska, P., Bukowska-Ośko, I., Kucia, M., Popiel, M., … Kaczyńska, K. Mitochondrial oxidative stress—a causative factor and therapeutic target in many diseases, 22 International Journal of Molecular Sciences § (2021). Multidisciplinary Digital Publishing Institute. Retrieved 14 November 2025 from https://doi.org/10.3390/ijms222413384

Kowalski, G. M., Carey, A. L., Selathurai, A., Kingwell, B. A., & Bruce, C. R. (2013). Plasma sphingosine-1-phosphate is elevated in obesity. *PLoS One*, 8(9), 72449. Retrieved from https://doi.org/10.1371/journal.pone.0072449

Kozlova, E. V., Denys, M. E., Benedum, J., Valdez, M. C., Enriquez, D., Bishay, A. E., … Curras-Collazo, M. C. (2022). Developmental exposure to indoor flame retardants and hypothalamic molecular signatures: Sex-dependent reprogramming of lipid homeostasis. *Frontiers in Endocrinology*, 13, 997304. Retrieved from https://doi.org/10.3389/FENDO.2022.997304/FULL

Kulkarni, R. N., Winnay, J. N., Daniels, M., Brüning, J. C., Flier, S. N., Hanahan, D., & Kahn, C. R. (1999). Altered function of insulin receptor substrate-1–deficient mouse islets and cultured β-cell lines. *The Journal of Clinical Investigation*, 104(12), R69–R75. Retrieved from https://doi.org/10.1172/JCI8339

La Merrill, M. A., Smith, M. T., McHale, C. M., Heindel, J. J., Atlas, E., Cave, M. C., … Blumberg, B. (2025). Consensus on the key characteristics of metabolism disruptors. *Nature Reviews. Endocrinology*, 21(4), 245–261. Retrieved 8 January 2026 from https://doi.org/10.1038/S41574-024-01059-8

Le Mentec, H. (2023). Impact of Endocrine Disruptors on Hepatic Steatosis and Its Pathological Progression. *Toxics*. Retrieved from https://doi.org/10.3390/toxics11070624 PMID- 37500508

Lee, A. H., Oh, J. H., Kim, H. S., Shin, J. H., Yoon, E. L., & Jun, D. W. (2022). Peripheral blood mononuclear cell mitochondrial copy number and adenosine triphosphate inhibition test in NAFLD. *Frontiers in Endocrinology*, 13. Retrieved 8 January 2026 from https://doi.org/10.3389/FENDO.2022.967848/PDF

Lee, E. J., Hong, Y. P., & Yang, Y. J. (2024). Short-term exposure to di(2-ethylhexyl)phthalate may disrupt hepatic lipid metabolism through modulating the oxidative stress in male adolescent rats. *Environmental Analysis Health and Toxicology*, 39(1). Retrieved 14 November 2025 from https://doi.org/10.5620/eaht.2024007

Lee, J. J. W., Alamleh, S., Zhan, L. J., Hueniken, K., Mahler, M. B., Billfalk-Kelly, A., … Liu, G. F. (2024). Clinical Predictors of Cisplatin Chemoradiation-Induced Ototoxicity in HPV-Positive Oropharyngeal Squamous Cell Carcinoma: A Case-Control Study. *JOURNAL OF OTOLARYNGOLOGY-HEAD & NECK SURGERY*, 53. Retrieved from https://doi.org/10.1177/19160216241248671

Lee, K., Haddad, A., Osme, A., Kim, C., Borzou, A., Ilchenko, S., … Kasumov, T. (2018). Hepatic mitochondrial defects in a nonalcoholic fatty liver disease mouse model are associated with increased degradation of oxidative phosphorylation subunits. *Molecular and Cellular Proteomics*, 17(12), 2371–2386. Retrieved from https://doi.org/10.1074/mcp.RA118.000961

Lefebvre, P., Benomar, Y., & Staels, B. (2010). Retinoid X receptors: common heterodimerization partners with distinct functions. *Trends in Endocrinology & Metabolism*, 21(11), 676–683. Retrieved 7 January 2026 from https://doi.org/10.1016/J.TEM.2010.06.009

Legaki, A. I., Moustakas, I. I., Sikorska, M., Papadopoulos, G., Velliou, R. I., & Chatzigeorgiou, A. (2022). Hepatocyte Mitochondrial Dynamics and Bioenergetics in Obesity-Related Non-Alcoholic Fatty Liver Disease. *Current Obesity Reports*, 11(3), 126–143. Retrieved from https://doi.org/10.1007/s13679-022-00473-1

Li, A. M., & Ye, J. (2020). The PHGDH enigma: Do cancer cells only need serine or also a redox modulator? *Cancer Letters*, 476, 97–105. Retrieved 8 January 2026 from https://doi.org/10.1016/J.CANLET.2020.01.036

Li, G., Zhao, C. Y., Wu, Q., Guan, S. yuan, Jin, H. W., Na, X. L., & Zhang, Y. B. (2021). Integrated metabolomics and transcriptomics reveal di(2-ethylhexyl) phthalate-induced mitochondrial dysfunction and glucose metabolism disorder through oxidative stress in rat liver. *Ecotoxicology and Environmental Safety*, 228, 112988. Retrieved from https://doi.org/10.1016/J.ECOENV.2021.112988

Li, J., Yan, H., Xiang, R., Yang, W., Ye, J., Yin, R., … Chi, Y. (2022). ATP Secretion and Metabolism in Regulating Pancreatic Beta Cell Functions and Hepatic Glycolipid Metabolism. *Frontiers in Physiology*, 13. Retrieved 8 January 2026 from https://doi.org/10.3389/FPHYS.2022.918042

Li, L., Huang, L., Lei, R., Zhang, P., Yang, Y., Liu, H., & Zhang, Y. (2023). DEHP and DBP, common phthalates, induce glucose metabolism disorders in rats via oxidative damage of PI3K/Akt/GLUT4 signaling. *Environmental Pollution*, 341, 122948. Retrieved from https://doi.org/10.1016/j.envpol.2023.122948

Li, X., Fang, E. F., Scheibye-Knudsen, M., Cui, H., Qiu, L., Li, J., … Guo, H. (2014). Di-(2-ethylhexyl) phthalate inhibits DNA replication leading to hyperPARylation, SIRT1 attenuation and mitochondrial dysfunction in the testis. *Scientific Reports 2014 4:1*, 4(1), 6434-. Retrieved 8 January 2026 from https://doi.org/10.1038/srep06434

Liang, X., Liang, J., Zhang, S., Yan, H., & Luan, T. (2024). Di-2-ethylhexyl phthalate disrupts hepatic lipid metabolism in obese mice by activating the LXR/SREBP-1c and PPAR-α signaling pathways. *Science of the Total Environment*, 914, 169919. Retrieved from https://doi.org/10.1016/j.scitotenv.2024.169919

Lin, T.-Y., Cantley, L. C., DeNicola, G. M., Lin, T.-Y., Cantley, L. C., & DeNicola, G. M. (2016). NRF2 Rewires Cellular Metabolism to Support the Antioxidant Response. *A Master Regulator of Oxidative Stress - The Transcription Factor Nrf2*. Retrieved 8 January 2026 from https://doi.org/10.5772/65141

Lin, Y., Wei, J., Li, Y., Chen, J., Zhou, Z., Song, L., … Xu, S. (2011). Developmental exposure to di(2-ethylhexyl) phthalate impairs endocrine pancreas and leads to long-term adverse effects on glucose homeostasis in the rat. *American Journal of Physiology. Endocrinology and Metabolism*, 301(3), 527–538. Retrieved 8 January 2026 from https://doi.org/10.1152/AJPENDO.00233.2011

Lipke, K., Kubis-Kubiak, A., & Piwowar, A. (2022). Molecular Mechanism of Lipotoxicity as an Interesting Aspect in the Development of Pathological States—Current View of Knowledge. *Cells*, 11(5), 844. Retrieved 28 August 2025 from https://doi.org/10.3390/CELLS11050844

Liu, J., Luo, R., Zhang, Y., & Li, X. (2024). Current status and perspective on molecular targets and therapeutic intervention strategy in hepatic ischemia-reperfusion injury. *Clinical and Molecular Hepatology*, 30(4), 585. Retrieved 8 January 2026 from https://doi.org/10.3350/CMH.2024.0222

Liu, Q., Bengmark, S., & Qu, S. (2010). The role of hepatic fat accumulation in pathogenesis of non-alcoholic fatty liver disease (NAFLD). *Lipids in Health and Disease*, 9, 1–9. Retrieved from https://doi.org/10.1186/1476-511X-9-42

Liu, X., Liu, B., Wang, J., Liu, H., Wu, J., Qi, Y., … Li, R. (2025). PHGDH activation fuels glioblastoma progression and radioresistance via serine synthesis pathway. *Journal of Experimental & Clinical Cancer Research : CR*, 44(1). Retrieved 8 January 2026 from https://doi.org/10.1186/S13046-025-03361-3

Lovero, D., Giordano, L., Marsano, R. M., Sanchez-Martinez, A., Boukhatmi, H., Drechsler, M., … Caggese, C. (2018). Characterization of Drosophila ATPsynC mutants as a new model of mitochondrial ATP synthase disorders. *PLOS ONE*, 13(8), e0201811. Retrieved 8 January 2026 from https://doi.org/10.1371/JOURNAL.PONE.0201811

Lustig, R. H., Collier, D., Kassotis, C., Roepke, T. A., Ji Kim, M., Blanc, E., … Heindel, J. J. (2022). Obesity I: Overview and molecular and biochemical mechanisms. *Biochemical Pharmacology*, 199(April), 115012. Retrieved from https://doi.org/10.1016/j.bcp.2022.115012

Mah, M., Febbraio, M., & Turpin-Nolan, S. (2021). Circulating Ceramides- Are Origins Important for Sphingolipid Biomarkers and Treatments? *Frontiers in Endocrinology*, 12. Retrieved from https://doi.org/10.3389/fendo.2021.684448

Maldonado, E., Morales-Pison, S., Urbina, F., Solari, A., Maldonado, E., Morales-Pison, S., … Solari, A. (2023). Aging Hallmarks and the Role of Oxidative Stress. *Antioxidants 2023, Vol. 12,* 12(3). Retrieved 7 January 2026 from https://doi.org/10.3390/ANTIOX12030651

Mantena, S. K., Vaughn, D. P., Andringa, K. K., Eccleston, H. B., King, A. L., Abrams, G. A., … Bailey, S. M. (2008). High fat diet induces dysregulation of hepatic oxygen gradients and mitochondrial function in vivo. *Biochemical Journal*, 417(Pt 1), 183. Retrieved 8 January 2026 from https://doi.org/10.1042/BJ20080868

Maradonna, F., Evangelisti, M., Gioacchini, G., Migliarini, B., Olivotto, I., & Carnevali, O. (2013). Assay of vtg, ERs and PPARs as endpoint for the rapid in vitro screening of the harmful effect of Di-(2-ethylhexyl)-phthalate (DEHP) and phthalic acid (PA) in zebrafish primary hepatocyte cultures. *Toxicology in Vitro*, 27(1), 84–91. Retrieved from https://doi.org/10.1016/j.tiv.2012.09.018

Martinez-Botas, J., Anderson, J. B., Tessier, D., Lapillonne, A., Chang, B. H. J., Quast, M. J., … Chan, L. (2000). Absence of perilipin results in leanness and reverses obesity in Lepr(db/db) mice. *Nature Genetics*, 26(4), 474–479. Retrieved from https://doi.org/10.1038/82630

Mashek, D. G. (2021). Hepatic lipid droplets: A balancing act between energy storage and metabolic dysfunction in NAFLD. *Molecular Metabolism*, 50, 101115. Retrieved 14 November 2025 from https://doi.org/10.1016/J.MOLMET.2020.101115

Meijnikman, A. S., Lappa, D., Herrema, H., Aydin, O., Krautkramer, K. A., Tremaroli, V., … Nieuwdorp, M. (2022). A systems biology approach to study non-alcoholic fatty liver (NAFL) in women with obesity. *IScience*, 25(8). Retrieved from https://doi.org/10.1016/j.isci.2022.104828

Merret, P.-E., Sparfel, L., Lavau, C., Lagadic-Gossmann, D., & Martin-Chouly, C. (2024). Extracellular vesicles as a potential source of biomarkers for endocrine disruptors in MASLD: A short review on the case of DEHP. *Biochimie*. Retrieved from https://doi.org/https://doi.org/10.1016/j.biochi.2024.09.009

Misrani, A., Tabassum, S., & Yang, L. (2021). Mitochondrial Dysfunction and Oxidative Stress in Alzheimer’s Disease. *Frontiers in Aging Neuroscience*, 13, 617588. Retrieved 7 January 2026 from https://doi.org/10.3389/FNAGI.2021.617588/FULL

Moliterni, C., Vari, F., Schifano, E., Tacconi, S., Stanca, E., Friuli, M., … Giudetti, A. M. (2024). Lipotoxicity of palmitic acid is associated with DGAT1 downregulation and abolished by PPARα activation in liver cells. *Journal of Lipid Research*, 65(12), 100692. Retrieved 28 August 2025 from https://doi.org/10.1016/J.JLR.2024.100692

Morino, K., Neschen, S., Bilz, S., Sono, S., Tsirigotis, D., Reznick, R. M., … Shulman, G. I. (2008). Muscle-pecific IRS-1 ser → ala transgenic mice are protected from fat-induced insulin resistance in skeletal muscle. *Diabetes*, 57(10), 2644–2651. Retrieved from https://doi.org/10.2337/db06-0454

Morris, G., & Berk, M. (2015). The many roads to mitochondrial dysfunction in neuroimmune and neuropsychiatric disorders. *BMC Medicine 2015 13:1*, 13(1), 68-. Retrieved 7 January 2026 from https://doi.org/10.1186/S12916-015-0310-Y

Mu, W., Xuefang, C., Liu, Y., Qianzhou, L., Gaolin, L., Jigang, Z., & Xiaoyu, L. (2019). Potential nexus of non-alcoholic fatty liver disease and type 2 diabetes mellitus: Insulin resistance between hepatic and peripheral tissues. *Frontiers in Pharmacology*, 9(JAN). Retrieved from https://doi.org/10.3389/fphar.2018.01566

Mukai, E., Fujimoto, S., & Inagaki, N. (2022). Role of Reactive Oxygen Species in Glucose Metabolism Disorder in Diabetic Pancreatic β-Cells. *Biomolecules*, 12(9). Retrieved 8 January 2026 from https://doi.org/10.3390/BIOM12091228

Mukhopadhyay, P., Horváth, B., Zsengeller, Z., Bátkai, S., Cao, Z., Kechrid, M., … Pacher, P. (2012). Mitochondrial reactive oxygen species generation triggers inflammatory response and tissue injury associated with hepatic ischemia–reperfusion: Therapeutic potential of mitochondrially targeted antioxidants. *Free Radical Biology and Medicine*, 53(5), 1123–1138. Retrieved 8 January 2026 from https://doi.org/10.1016/J.FREERADBIOMED.2012.05.036

Mullarky, E., & Cantley, L. C. (2015). Diverting Glycolysis to Combat Oxidative Stress. *Innovative Medicine*, 3–23. Retrieved 9 August 2024 from https://doi.org/10.1007/978-4-431-55651-0_1

Murphy, M. P. (2009). How mitochondria produce reactive oxygen species. *Biochemical Journal*, 417(1), 1–13. Retrieved 7 January 2026 from https://doi.org/10.1042/BJ20081386

Murray-Cors, S., Owens, M., Liew, Y. T., Day, M., Cairns, W., & Prokop, A. (2025). Impacts of mitochondrial dysfunction on axonal microtubule bundles as a potential mechanism of neurodegeneration. *Frontiers in Neuroscience*, 19, 1631752. Retrieved from https://doi.org/10.3389/FNINS.2025.1631752/FULL

Muyyarikkandy, M. S., McLeod, M., Maguire, M., Mahar, R., Kattapuram, N., Zhang, C., … Sunny, N. E. (2020). Branched chain amino acids and carbohydrate restriction exacerbate ketogenesis and hepatic mitochondrial oxidative dysfunction during NAFLD. *FASEB Journal*, 34(11), 14832–14849. Retrieved 14 November 2025 from https://doi.org/10.1096/FJ.202001495R;REQUESTEDJOURNAL:JOURNAL:15306860;WGROUP:STRING:PUBLICATION

Myint, M., Oppedisano, F., De Giorgi, V., Kim, B. M., Marincola, F. M., Alter, H. J., & Nesci, S. (2023). Inflammatory signaling in NASH driven by hepatocyte mitochondrial dysfunctions. *Journal of Translational Medicine*, 21(1). Retrieved 8 January 2026 from https://doi.org/10.1186/S12967-023-04627-0

Nakamura, T., & Lipton, S. A. (2017). ’sNO’-Storms Compromise Protein Activity and Mitochondrial Metabolism in Neurodegenerative Disorders. *Trends in Endocrinology and Metabolism*, 28(12), 879–892. Retrieved 7 January 2026 from https://doi.org/10.1016/j.tem.2017.10.004

Niranjan, R. (2023). Uncoupling hepatic insulin resistance–hepatic inflammation to improve insulin sensitivity and to prevent impaired metabolism-associated fatty liver disease in type 2 diabetes. *Neuroscience & Biobehavioral Reviews*. Retrieved from https://doi.org/10.1016/j.neubiorev.2023.105026

Nonarath, H. J., Hall, A. E., SenthilKumar, G., Abroe, B., Eells, J. T., & Liedhegner, E. (2021). 670nm Photobiomodulation Modulates Bioenergetics And Oxidative Stress, Decreasing Inflammatory Mediator Production In An In Vitro Model Of Diabetic Retinopathy. Retrieved 8 January 2026 from https://doi.org/10.21203/RS.3.RS-185179/V1

OECD. (2018). Users Handbook supplement to the Guidance Document for developing and assessing Adverse Outcome Pathways, OECD Series on Adverse Outcome Pathways, No. 1, OECD Publishing, Paris. *Organisation for Economic Co-Operation and Development Publishing*, (1). Retrieved 19 November 2025 from https://doi.org/10.1787/5jlv1m9d1g32-en

Pafili, K., & Roden, M. (2021). Nonalcoholic fatty liver disease (NAFLD) from pathogenesis to treatment concepts in humans. *Molecular Metabolism*, 50, 101122. Retrieved 23 December 2023 from https://doi.org/10.1016/j.molmet.2020.101122

Palma, F. R., Gantner, B. N., Sakiyama, M. J., Kayzuka, C., Shukla, S., Lacchini, R., … Bonini, M. G. (2023). ROS production by mitochondria: function or dysfunction? *Oncogene 2023 43:5*, 43(5), 295–303. Retrieved 14 November 2025 from https://doi.org/10.1038/s41388-023-02907-z

Pan, K., Xu, J., Xu, Y., Wang, C., & Yu, J. (2024). The association between endocrine disrupting chemicals and nonalcoholic fatty liver disease: A systematic review and meta-analysis. *Pharmacological Research*, 205(April), 107251. Retrieved from https://doi.org/10.1016/j.phrs.2024.107251

Pan, X., Zheng, L., Ding, Y., Dai, Z., Qi, X., Zhang, X., … Qiao, Q. (2022). Effects of di-(2-ethylhexyl) phthalate on oxidative stress and lipid metabolism in livers of male Xenopus tropicalis. Retrieved 8 January 2026 from https://doi.org/10.21203/rs.3.rs-1595962/v1

Park, C. G., Sung, B., Ryu, C. S., & Kim, Y. J. (2020). Mono-(2-ethylhexyl) phthalate induces oxidative stress and lipid accumulation in zebrafish liver cells. *Comparative Biochemistry and Physiology Part - C: Toxicology and Pharmacology*, 230, 108704. Retrieved from https://doi.org/10.1016/j.cbpc.2020.108704

Park, S. Y., Moon, M. H., Jeong, J. K., Lee, J. H., Park, Y. G., Lee, Y. J., & Seol, J. W. (2012). Antiobesity activity of a sphingosine 1-phosphate analogue FTY720 observed in adipocytes and obese mouse model. *Experimental and Molecular Medicine*, 44(10), 603–614. Retrieved 14 November 2025 from https://doi.org/10.3858/emm.2012.44.10.069

Pei, D., Jia, P.-P. C. I. of G. and I. T., Xin, G.-Y., Junaid, M., Wang, Y., & Ma, Y.-B. (2020, March 25). DEHP chronic exposure disturbs the gut microbial community and metabolic homeostasis: Gender-based differences in zebrafish. Retrieved 8 January 2026 from https://doi.org/10.21203/rs.3.rs-18516/v1

Peng, M. (2022). Phthalates and Obesity: Examining the Metabolism-disrupting Chemical Hypothesis in Midlife Women. Retrieved 8 January 2026 from https://doi.org/10.7302/6134

Pérez-Albaladejo, E., Solé, M., & Porte, C. (2020). Plastics and plastic additives as inducers of oxidative stress. *Current Opinion in Toxicology*, 20–21, 69–76. Retrieved from https://doi.org/10.1016/J.COTOX.2020.07.002

Petersen, M. C., & Shulman, G. I. (2017). Roles of Diacylglycerols and Ceramides in Hepatic Insulin Resistance. *Trends in Pharmacological Sciences*, 38(7), 649–665. Retrieved from https://doi.org/10.1016/j.tips.2017.04.004

Pietrocola, F., Galluzzi, L., Bravo-San Pedro, J. M., Madeo, F., & Kroemer, G. (2015). Acetyl Coenzyme A: A Central Metabolite and Second Messenger. *Cell Metabolism*, 21(6), 805–821. Retrieved 14 November 2025 from https://doi.org/10.1016/J.CMET.2015.05.014

Qian, L., Zhu, Y., Deng, C., Liang, Z., Chen, J., Chen, Y., … Yang, Y. (2024). Peroxisome proliferator-activated receptor gamma coactivator-1 (PGC-1) family in physiological and pathophysiological process and diseases. *Signal Transduction and Targeted Therapy*, 9(1). Retrieved from https://doi.org/10.1038/s41392-024-01756-w

Rajagopal, G., Bhaskaran, R. S., & Karundevi, B. (2019a). Developmental exposure to DEHP alters hepatic glucose uptake and transcriptional regulation of GLUT2 in rat male offspring. *Toxicology*, 413, 56–64. Retrieved 8 January 2026 from https://doi.org/10.1016/j.tox.2018.12.004

Rajagopal, G., Bhaskaran, R. S., & Karundevi, B. (2019b). Maternal di-(2-ethylhexyl) phthalate exposure alters hepatic insulin signal transduction and glucoregulatory events in rat F₁ male offspring. *Journal of Applied Toxicology*, 39(5), 751. Retrieved from https://doi.org/10.1002/jat.3764

Ramanathan, R., Ali, A. H., & Ibdah, J. A. (2022). Mitochondrial Dysfunction Plays Central Role in Nonalcoholic Fatty Liver Disease. *International Journal of Molecular Sciences*, 23(13). Retrieved from https://doi.org/10.3390/ijms23137280

Rector, R. S., Morris, E. M., Ridenhour, S., Meers, G. M., Hsu, F. F., Turk, J., & Ibdah, J. A. (2013). Selective hepatic insulin resistance in a murine model heterozygous for a mitochondrial trifunctional protein defect. *Hepatology*, 57(6), 2213–2223. Retrieved from https://doi.org/10.1002/hep.26285

Reddam, A., McLarnan, S., & Kupsco, A. (2022). Environmental Chemical Exposures and Mitochondrial Dysfunction: a Review of Recent Literature. *Current Environmental Health Reports*, 9(4), 631–649. Retrieved 14 November 2025 from https://doi.org/10.1007/S40572-022-00371-7/FIGURES/2

Regnault, C., Usal, M., Veyrenc, S., Couturier, K., Batandier, C., Bulteau, A. L., … Reynaud, S. (2018). Unexpected metabolic disorders induced by endocrine disruptors in Xenopus tropicalis provide new lead for understanding amphibian decline. *Proceedings of the National Academy of Sciences of the United States of America*, 115(19), E4416–E4425. Retrieved 7 January 2026 from https://doi.org/10.1073/PNAS.1721267115;PAGEGROUP:STRING:PUBLICATION

Régnier, M., Polizzi, A., Guillou, H., & Loiseau, N. (2019). Sphingolipid metabolism in non-alcoholic fatty liver diseases. *Biochimie*. Retrieved 27 May 2024 from https://doi.org/10.1016/j.biochi.2018.07.021

Rengarajan, S., Parthasarathy, C., Anitha, M., & Balasubramanian, K. (2007). Diethylhexyl phthalate impairs insulin binding and glucose oxidation in Chang liver cells. *Toxicology in Vitro*, 21(1), 99–102. Retrieved from https://doi.org/10.1016/j.tiv.2006.07.005

Rui, L. (2014). Energy Metabolism in the Liver. *Comprehensive Physiology*, 4(1), 177–197. Retrieved 7 January 2026 from https://doi.org/10.1002/J.2040-4603.2014.TB00548.X

Rusyn, I., Peters, J., & Cunningham, M. (2006). Modes of Action and Species-Specific Effects of Di-(2-ethylhexyl)Phthalate in the Liver. *Critical Reviews in Toxicology*, 36(5), 459–479. Retrieved 23 December 2023 from https://doi.org/10.1080/10408440600779065

Ryu, J. Y., Whang, J., Park, H., Im, J. Y., Kim, J., Ahn, M. Y., … Kim, S. H. (2007). Di(2-ethylhexyl) phthalate induces apoptosis through peroxisome proliferators-activated receptor-gamma and ERK 1/2 activation in testis of Sprague-Dawley rats. *Journal of Toxicology and Environmental Health - Part A: Current Issues*, 70(15–16), 1296–1303. Retrieved from https://doi.org/10.1080/15287390701432160

Sabens Liedhegner, E. A., Gao, X. H., & Mieyal, J. J. (2012). Mechanisms of altered redox regulation in neurodegenerative diseases--focus on S--glutathionylation. *Antioxidants & Redox Signaling*, 16(6), 543–566. Retrieved 7 January 2026 from https://doi.org/10.1089/ARS.2011.4119

Sajan, M. P., Ivey, R. A., & Farese, R. V. (2015). BMI-related progression of atypical PKC-dependent aberrations in insulin signaling through IRS-1, Akt, FoxO1 and PGC-1α in livers of obese and type 2 diabetic humans. *Metabolism: Clinical and Experimental*, 64(11), 1454–1465. Retrieved 24 October 2024 from https://doi.org/10.1016/J.METABOL.2015.08.011

Samala, N., Tersey, S. A., Chalasani, N., Anderson, R. M., & Mirmira, R. G. (2017). Molecular mechanisms of nonalcoholic fatty liver disease: Potential role for 12-lipoxygenase. *Journal of Diabetes and Its Complications*, 31(11), 1630–1637. Retrieved 14 November 2025 from https://doi.org/10.1016/J.JDIACOMP.2017.07.014

Sampey, B. P., Freemerman, A. J., Zhang, J., Kuan, P. F., Galanko, J. A., O’Connell, T. M., … Makowski, L. (2012). Metabolomic Profiling Reveals Mitochondrial-Derived Lipid Biomarkers That Drive Obesity-Associated Inflammation. *PLOS ONE*, 7(6), e38812. Retrieved 28 August 2025 from https://doi.org/10.1371/JOURNAL.PONE.0038812

Samuel, V. T., & Shulman, G. I. (2016). The pathogenesis of insulin resistance: integrating signaling pathways and substrate flux. *The Journal of Clinical Investigation*, 126(1), 12–22. Retrieved from https://doi.org/10.1172/JCI77812

San-Millán, I. (2023). The Key Role of Mitochondrial Function in Health and Disease. *Antioxidants*, 12(4). Retrieved from https://doi.org/10.3390/antiox12040782

Santoleri, D., & Titchenell, P. M. (2019). Resolving the Paradox of Hepatic Insulin Resistance. *Cmgh*, 7(2), 447–456. Retrieved from https://doi.org/10.1016/j.jcmgh.2018.10.016

Sargis, R. M., & Simmons, R. A. (2019). Environmental neglect: endocrine disruptors as underappreciated but potentially modifiable diabetes risk factors. *Diabetologia*, 62(10), 1811–1822. Retrieved from https://doi.org/10.1007/s00125-019-4940-z

Schulz, H. (2013). Fatty Acid Oxidation. *Encyclopedia of Biological Chemistry: Second Edition*. Retrieved from https://doi.org/10.1016/B978-0-12-378630-2.00071-2

Scoditti, E., Sabatini, S., Carli, F., & Gastaldelli, A. (2024). Hepatic glucose metabolism in the steatotic liver. *Nature Reviews Gastroenterology and Hepatology*, 21(5), 319–334. Retrieved from https://doi.org/10.1038/s41575-023-00888-8

Sen, P., Fan, Y., Schlezinger, J. J., Ehrlich, S. D., Webster, T. F., Hyötyläinen, T., … Orešič, M. (2024). Exposure to environmental toxicants is associated with gut microbiome dysbiosis, insulin resistance and obesity. *Environment International*, 186, 108569. Retrieved 8 January 2026 from https://doi.org/10.1016/J.ENVINT.2024.108569

Sha, W., Hu, F., & Bu, S. (2020). Mitochondrial dysfunction and pancreatic islet &beta;‑cell failure (Review). *Experimental and Therapeutic Medicine*, 20(6), 1–1. Retrieved 7 January 2026 from https://doi.org/10.3892/ETM.2020.9396

Shan, S., & Hoffman, J. M. (2024). Serine metabolism in aging and age-related diseases. *GeroScience 2024 47:1*, 47(1), 611–630. Retrieved 14 November 2025 from https://doi.org/10.1007/S11357-024-01444-1

Shi, L., & Tu, B. P. (2015). Acetyl-CoA and the regulation of metabolism: mechanisms and consequences. *Current Opinion in Cell Biology*, 33, 125–131. Retrieved 14 November 2025 from https://doi.org/10.1016/J.CEB.2015.02.003

Shum, M., Ngo, J., Shirihai, O. S., & Liesa, M. (2021). Mitochondrial oxidative function in NAFLD: Friend or foe? *Molecular Metabolism*, 50, 101134. Retrieved from https://doi.org/10.1016/j.molmet.2020.101134

Souza, S. C., Muliro, K. V., Liscum, L., Lien, P., Yamamoto, M. T., Schaffer, J. E., … Greenberg, A. S. (2002). Modulation of hormone-sensitive lipase and protein kinase A-mediated lipolysis by perilipin A in an adenoviral reconstituted system. *Journal of Biological Chemistry*, 277(10), 8267–8272. Retrieved from https://doi.org/10.1074/jbc.M108329200

Spillier, Q., Vertommen, D., Ravez, S., Marteau, R., Thémans, Q., Corbet, C., … Frédérick, R. (2019). Anti-alcohol abuse drug disulfiram inhibits human PHGDH via disruption of its active tetrameric form through a specific cysteine oxidation. *Scientific Reports 2019 9:1*, 9(1), 4737-. Retrieved 14 November 2025 from https://doi.org/10.1038/s41598-019-41187-0

Srivastava, S., & Srivastava, S. (2017). The Mitochondrial Basis of Aging and Age-Related Disorders. *Genes 2017, Vol. 8,* 8(12). Retrieved 7 January 2026 from https://doi.org/10.3390/GENES8120398

Summers, S. A. (2006). Ceramides in insulin resistance and lipotoxicity. *Progress in Lipid Research*, 45(1), 42–72. Retrieved 28 August 2025 from https://doi.org/10.1016/J.PLIPRES.2005.11.002

Sun, F., Huang, Y., Chen, H., Huang, J., Zhang, L., Wei, S., … Huang, W. (2023). BPA and its alternatives BPF and BPAF exaggerate hepatic lipid metabolism disorders in male mice fed a high fat diet. *Science of the Total Environment*, 867. Retrieved 11 July 2023 from https://doi.org/10.1016/j.scitotenv.2023.161521

Takahashi, C., Kurano, M., Nishikawa, M., Kano, K., Dohi, T., Miyauchi, K., … Yatomi, Y. (2017). Vehicle-dependent effects of sphingosine 1-phosphate on plasminogen activator inhibitor-1 expression. *Journal of Atherosclerosis and Thrombosis*, 24(9), 954–969. Retrieved from https://doi.org/10.5551/jat.37663

Tansey, J. T., Sztalryd, C., Gruia-Gray, J., Roush, D. L., Zee, J. V., Gavrilova, O., … Londos, C. (2001). Perilipin ablation results in a lean mouse with aberrant adipocyte lipolysis, enhanced leptin production, and resistance to diet-induced obesity. *Proceedings of the National Academy of Sciences of the United States of America*, 98(11), 6494–6499. Retrieved from https://doi.org/10.1073/pnas.101042998

Tansey, John T., Huml, A. M., Vogt, R., Davis, K. E., Jones, J. M., Fraser, K. A., … Londos, C. (2003). Functional studies on native and mutated forms of perilipins: A role in protein kinase a-mediated lipolysis of triacylglycerols in Chinese hamster ovary cells. *Journal of Biological Chemistry*, 278(10), 8401–8406. Retrieved from https://doi.org/10.1074/jbc.M211005200

Tanti, J. F., & Jager, J. (2009). Cellular mechanisms of insulin resistance: role of stress-regulated serine kinases and insulin receptor substrates (IRS) serine phosphorylation. *Current Opinion in Pharmacology*, 9(6), 753–762. Retrieved from https://doi.org/10.1016/j.coph.2009.07.004

Teodoro, J. S., Rolo, A. P., Duarte, F. V., Simões, A. M., & Palmeira, C. M. (2008). Differential alterations in mitochondrial function induced by a choline-deficient diet: Understanding fatty liver disease progression. *Mitochondrion*, 8(5–6), 367–376. Retrieved 8 January 2026 from https://doi.org/10.1016/j.mito.2008.07.008

Thayer, T. E., Lino Cardenas, C. L., Martyn, T., Nicholson, C. J., Traeger, L., Wunderer, F., … Malhotra, R. (2020). The Role of Bone Morphogenetic Protein Signaling in Non-Alcoholic Fatty Liver Disease. *Scientific Reports*, 10(1). Retrieved 14 November 2025 from https://doi.org/10.1038/S41598-020-66770-8

Titchenell, P. M., Quinn, W. J., Lu, M., Chu, Q., Lu, W., Li, C., … Birnbaum, M. J. (2016). Direct Hepatocyte Insulin Signaling Is Required for Lipogenesis but Is Dispensable for the Suppression of Glucose Production. *Cell Metabolism*, 23(6), 1154–1166. Retrieved 24 October 2024 from https://doi.org/10.1016/J.CMET.2016.04.022

Trushina, E., Nguyen, T. K. O., & Trushin, S. (2023). Modulation of Mitochondrial Function as a Therapeutic Strategy for Neurodegenerative Diseases. *Journal of Prevention of Alzheimer’s Disease*, 10(4), 675–685. Retrieved 8 January 2026 from https://doi.org/10.14283/jpad.2023.108

Uren-Webster, T. M., Lewis, C., Filby, A. L., Paull, G. C., & Santos, E. M. (2010). Mechanisms of toxicity of di(2-ethylhexyl) phthalate on the reproductive health of male zebrafish. *Aquatic Toxicology*, 99(3), 360–369. Retrieved from https://doi.org/10.1016/j.aquatox.2010.05.015

Useini, A., Engelberger, F., Künze, G., & Sträter, N. (2023). Structural basis of the activation of PPARγ by the plasticizer metabolites MEHP and MINCH. *Environment International*, 173, 107822. Retrieved from https://doi.org/10.1016/j.envint.2023.107822

van Eunen, K., Simons, S. M. J., Gerding, A., Bleeker, A., den Besten, G., Touw, C. M. L., … Bakker, B. M. (2013). Biochemical Competition Makes Fatty-Acid β-Oxidation Vulnerable to Substrate Overload. *PLOS Computational Biology*, 9(8), e1003186. Retrieved 7 January 2026 from https://doi.org/10.1371/JOURNAL.PCBI.1003186

Vela-Guajardo, J. E., Garza-González, S., & García, N. (2020). Glucolipotoxicity-induced Oxidative Stress is Related to Mitochondrial Dysfunction and Apoptosis of Pancreatic β-cell. *Current Diabetes Reviews*, 17(5), 7–17. Retrieved 7 January 2026 from https://doi.org/10.2174/1573399816666201103142102

Venkata, N. G., Robinson, J. A., Cabot, P. J., Davis, B., Monteith, G. R., Roberts-Thomson, S. J., … Roberts-Thomson, S. J. (2006). Mono (2-ethylhexyl) phthalate and mono-n-butyl phthalate activation of peroxisome proliferator activated-receptors α and γ in breast. *Toxicology Letters*, 163(3), 224–234. Retrieved 22 December 2023 from https://doi.org/10.1016/j.toxlet.2005.11.001

Vinayagamoorthi, R., Bobby, Z., & Sridhar, M. G. (2008). Antioxidants preserve redox balance and inhibit c-Jun-N-terminal kinase pathway while improving insulin signaling in fat-fed rats: evidence for the role of oxidative stress on IRS-1 serine phosphorylation and insulin resistance. *The Journal of Endocrinology*, 197(2), 287–296. Retrieved 14 November 2025 from https://doi.org/10.1677/JOE-08-0061

Wajner, M., & Amaral, A. U. (2016). Mitochondrial dysfunction in fatty acid oxidation disorders: insights from human and animal studies. *Bioscience Reports*, 36(1), e00281. Retrieved 28 August 2025 from https://doi.org/10.1042/BSR20150240

Walther, T. C., & Farese, R. V. (2012). Lipid droplets and cellular lipid metabolism. *Annual Review of Biochemistry*, 81, 687–714. Retrieved from https://doi.org/10.1146/annurev-biochem-061009-102430

Wang, F., Chang, C., Li, R., Zhang, Z., Jiang, H., Zeng, N., … Wang, Q. (2019). Retinol binding protein 4 mediates MEHP-induced glucometabolic abnormalities in HepG2 cells. *Toxicology*, 424, 152236. Retrieved from https://doi.org/10.1016/j.tox.2019.06.007

Wang, M., Wang, Y., Han, J., Duan, Z., Yin, J., Ding, R., … Wang, Q. (2023). Gestational and Lactational Co-Exposure to DEHP and BPA Impairs Hepatic Function via PI3K/AKT/FOXO1 Pathway in Offspring. *Toxics 2023, Vol. 11,* 11(3). Retrieved 14 November 2025 from https://doi.org/10.3390/TOXICS11030216

Wei, X., Yang, D., Zhang, B., Fan, X., Du, H., Zhu, R., … Gu, N. (2022). Di-(2-ethylhexyl) phthalate increases plasma glucose and induces lipid metabolic disorders via FoxO1 in adult mice. *Science of The Total Environment*, 842, 156815. Retrieved from https://doi.org/10.1016/J.SCITOTENV.2022.156815

Weigert, C., Hennige, A. M., Brischmann, T., Beck, A., Moeschel, K., Schäuble, M., … Lehmann, R. (2005). The phosphorylation of Ser318 of insulin receptor substrate 1 is not per se inhibitory in skeletal muscle cells but is necessary to trigger the attenuation of the insulin-stimulated signal. *Journal of Biological Chemistry*, 280(45), 37393–37399. Retrieved from https://doi.org/10.1074/jbc.M506134200

Wellen, K. E., Hatzivassiliou, G., Sachdeva, U. M., Bui, T. V., Cross, J. R., & Thompson, C. B. (2009). ATP-citrate lyase links cellular metabolism to histone acetylation. *Science*, 324(5930), 1076–1080. Retrieved 14 November 2025 from https://doi.org/10.1126/SCIENCE.1164097;WEBSITE:WEBSITE:AAAS-SITE;ISSUE:ISSUE:DOI

Wen, P., Sun, Z., Gou, F., Wang, J., Fan, Q., Zhao, D., & Yang, L. (2025). Oxidative stress and mitochondrial impairment: Key drivers in neurodegenerative disorders. *Ageing Research Reviews*, 104, 102667. Retrieved 8 January 2026 from https://doi.org/10.1016/J.ARR.2025.102667

Wigger, D., Schumacher, F., Schneider-Schaulies, S., & Kleuser, B. (2021). Sphingosine 1-phosphate metabolism and insulin signaling. *Cellular Signalling*, 82, 109959. Retrieved from https://doi.org/10.1016/j.cellsig.2021.109959

Wu, W., Yang, Y., Tan, B. K., Lin, S., Chen, Y., & Hu, J. (2023). Endocrine disruptors in foods: Overlooked factors contributing to the prevalence of obesity. *Https://Cjfs.Agriculturejournals.Cz/Doi/10.17221/80/2023-CJFS.Html*, 41(6), 393–405. Retrieved 8 January 2026 from https://doi.org/10.17221/80/2023-CJFS

Xu, J., Wang, L., Zhang, L., Zheng, F., Wang, F., Leng, J., … Xia, D. (2021). Mono-2-ethylhexyl phthalate drives progression of PINK1-parkin-mediated mitophagy via increasing mitochondrial ROS to exacerbate cytotoxicity. *Redox Biology*, 38, 101776. Retrieved 14 November 2025 from https://doi.org/10.1016/J.REDOX.2020.101776

Xu, P., Su, Y. ni, Ling, C., Wang, J., & Zhang, W. (2024). Mitochondrial dysfunction mediated by thioredoxin-interacting protein: A crucial determinant in di(2-ethylhexyl) phthalate-induced liver failure. *Ecotoxicology and Environmental Safety*, 272(1), 116103. Retrieved 10 February 2026 from https://doi.org/10.1016/j.ecoenv.2024.116103

Yang, A., & Mottillo, E. P. (2020). Adipocyte lipolysis: From molecular mechanisms of regulation to disease and therapeutics. *Biochemical Journal*, 477(5), 985–1008. Retrieved from https://doi.org/10.1042/BCJ20190468

Yang, Z.-J., Zhao, C.-L., Liang, W.-Q., Chen, Z.-R., Du, Z.-D., & Gong, S.-S. (2024). ROS-induced oxidative stress and mitochondrial dysfunction: a possible mechanism responsible for noise-induced ribbon synaptic damage. *American Journal of Translational Research*, 16(1), 272. Retrieved 8 January 2026 from https://doi.org/10.62347/EVDE9449

Yazıcı, D., & Sezer, H. (2017). Insulin resistance, obesity and lipotoxicity. *Advances in Experimental Medicine and Biology*, 960, 277–304. Retrieved 27 May 2024 from https://doi.org/10.1007/978-3-319-48382-5_12/FIGURES/4

Yi, D., Hou, Y., Wang, L., Ding, B., Yang, Z., Li, J., … Wu, G. (2014). Dietary N-acetylcysteine supplementation alleviates liver injury in lipopolysaccharide-challenged piglets. *British Journal of Nutrition*, 111(1), 46–54. Retrieved 8 January 2026 from https://doi.org/10.1017/S0007114513002171

Yki-Järvinen, H. (2009). Thiazolidinediones and the liver in humans. *Current Opinion in Lipidology*, 20(6), 477–483. Retrieved from https://doi.org/10.1097/MOL.0b013e3283321d37

Yoon, H., Shaw, J. L., Haigis, M. C., & Greka, A. (2021). Lipid metabolism in sickness and in health: emerging regulators of lipotoxicity. *Molecular Cell*, 81(18), 3708. Retrieved 28 August 2025 from https://doi.org/10.1016/J.MOLCEL.2021.08.027

Yu, D., Richardson, N. E., Green, C. L., Spicer, A. B., Murphy, M. E., Flores, V., … Lamming, D. W. (2021). The adverse metabolic effects of branched-chain amino acids are mediated by isoleucine and valine. *Cell Metabolism*, 33(5), 905-922.e6. Retrieved 31 July 2023 from https://doi.org/10.1016/j.cmet.2021.03.025

Yu, S., Meng, S., Xiang, M., & Ma, H. (2021, November 1). Phosphoenolpyruvate carboxykinase in cell metabolism: Roles and mechanisms beyond gluconeogenesis. *Molecular Metabolism*. Elsevier. Retrieved from https://doi.org/10.1016/j.molmet.2021.101257

Zabielski, P., Hady, H. R., Chacinska, M., Roszczyc, K., Gorski, J., & Blachnio-Zabielska, A. U. (2018). The effect of high fat diet and metformin treatment on liver lipids accumulation and their impact on insulin action. *Scientific Reports*, 8(1), 7249-. Retrieved 8 January 2026 from https://doi.org/10.1038/s41598-018-25397-6

Zeng, N., Huang, R., Li, N., Jiang, H., Li, R., Wang, F., … Wang, Q. (2018). MiR-451a attenuates free fatty acids–mediated hepatocyte steatosis by targeting the thyroid hormone responsive spot 14 gene. *Molecular and Cellular Endocrinology*, 474, 260–271. Retrieved 12 December 2025 from https://doi.org/10.1016/j.mce.2018.03.016

Zhang, C., Zhao, Y., Yu, M., Qin, J., Ye, B., Wang, Q., … Wang, Q. (2022). Mitochondrial Dysfunction and Chronic Liver Disease. *Current Issues in Molecular Biology 2022, Vol. 44, Pages 3156-3165*, 44(7), 3156–3165. Retrieved 8 January 2026 from https://doi.org/10.3390/CIMB44070218

Zhang, Haoyang, Ran, M., Jiang, L., Sun, X., Qiu, T., Li, J., … Yang, G. (2023). Mitochondrial dysfunction and endoplasmic reticulum stress induced by activation of PPARα leaded testicular to apoptosis in SD rats explored to di-(2-ethylhexyl) phthalate (DEHP). *Ecotoxicology and Environmental Safety*, 268, 115711. Retrieved 8 January 2026 from https://doi.org/10.1016/J.ECOENV.2023.115711

Zhang, Hong, Ben, Y., Han, Y., Zhang, Y., Li, Y., & Chen, X. (2022). Phthalate exposure and risk of diabetes mellitus: Implications from a systematic review and meta-analysis. *Environmental Research*, 204, 112109. Retrieved from https://doi.org/10.1016/j.envres.2021.112109

Zhang, W., Shen, X. yue, Zhang, W. wen, Chen, H., Xu, W. ping, & Wei, W. (2017). Di-(2-ethylhexyl) phthalate could disrupt the insulin signaling pathway in liver of SD rats and L02 cells via PPARγ. *Toxicology and Applied Pharmacology*, 316, 17–26. Retrieved 14 November 2025 from https://doi.org/10.1016/j.taap.2016.12.010

Zhang, X., Zhang, C., Chen, L., Han, X., & Ji, L. (2014). Human serum acylcarnitine profiles in different glucose tolerance states. *Diabetes Research and Clinical Practice*, 104(3), 376–382. Retrieved 28 August 2025 from https://doi.org/10.1016/J.DIABRES.2014.04.013

Zhang, Yifan, Feng, H., Tian, A., Zhang, C., Song, F., Zeng, T., & Zhao, X. (2023). Long-term exposure to low-dose Di(2-ethylhexyl) phthalate aggravated high fat diet-induced obesity in female mice. *Ecotoxicology and Environmental Safety*, 253. Retrieved 8 January 2026 from https://doi.org/10.1016/j.ecoenv.2023.114679

Zhang, Yu, Wen, P., Luo, J., Ding, H., Cao, H., He, W., … Jiang, L. (2021). Sirtuin 3 regulates mitochondrial protein acetylation and metabolism in tubular epithelial cells during renal fibrosis. *Cell Death & Disease*, 12(9), 847. Retrieved from https://doi.org/10.1038/s41419-021-04134-4

Zhang, Yuexia, Ge, S., Yang, Z., Li, Z., Gong, X., Zhang, Q., … Dong, C. (2020). Disturbance of di-(2-ethylhexyl) phthalate in hepatic lipid metabolism in rats fed with high fat diet. *Food and Chemical Toxicology*, 146, 111848. Retrieved 7 January 2026 from https://doi.org/10.1016/j.fct.2020.111848

Zhao, R. Z., Jiang, S., Zhang, L., & Yu, Z. Bin. (2019). Mitochondrial electron transport chain, ROS generation and uncoupling (Review). *International Journal of Molecular Medicine*, 44(1), 3–15. Retrieved from https://doi.org/10.3892/ijmm.2019.4188

Zhao, X., Fu, J., Du, J., & Xu, W. (2020). The Role of D-3-Phosphoglycerate Dehydrogenase in Cancer. *International Journal of Biological Sciences*, 16(9), 1495–1506. Retrieved 8 January 2026 from https://doi.org/10.7150/IJBS.41051

Zheng, Y., Wang, S., Wu, J., & Wang, Y. (2023). Mitochondrial metabolic dysfunction and non-alcoholic fatty liver disease: new insights from pathogenic mechanisms to clinically targeted therapy. *Journal of Translational Medicine 2023 21:1*, 21(1), 510-. Retrieved 14 November 2025 from https://doi.org/10.1186/S12967-023-04367-1

Zhou, M., Ford, B., Lee, D., Tindula, G., Huen, K., Tran, V., … Holland, N. (2018). Metabolomic Markers of Phthalate Exposure in Plasma and Urine of Pregnant Women. *Frontiers in Public Health*, 6. Retrieved from https://doi.org/10.3389/fpubh.2018.00298
