# Supplementary material for: Mapping DEHP-induced metabolic dysregulation through multi-omics and systems biology: an adverse outcome pathway network approach
Source: Front Toxicol. 2026 Sep 30;8:1930835. doi: 10.3389/ftox.2026.1930835 (PMC13661021; doi:10.3389/ftox.2026.1930835)
Supplement: Supplementary file 2 [file Supplementaryfile2.docx]

# Supplementary Material 2: Multi-Omics analysis

# HepaRG cells (3D)

### Cell culture & treatments

HepaRG cells were suspended at a density of 5x105 cells/ml in type I collagen and cells were maintained in supplemented William’s E medium (insulin 5 μg/ml, glutamine 2 mM, Fetal Bovine Serum (FBS) 10%, penicillin 100 U/ml, streptomycin 100 mg/ml and hydrocortisone hemisuccinate 25 mg/mL), which was replaced every two days. DEHP was added to the culture medium at a concentration of 100 pM, 100 nM, or 10 µM, with a final DMSO concentration of 0.1%. Hepoid® with HepaRG were treated for 14 days starting on the fifth day of culture. To prevent toxicity, cell viability was check by measuring intracellular ATP levels using the CellTiter-Glo 3D Assay from Promega (Madison, WI, USA) following the manufacturer’s protocol.

## Transcriptomics

### Sample Preparation

All steps followed the One-Color Microarray-Based Gene Expression Analysis (Low Input Quick Amp Labeling) Protocol version 6.9.1 supplied by Agilent Technologies. Briefly, samples were spiked with quality control Agilent RNA Spike-In mix in 1.5 µL of sample ranging from 10 to 200 ng total RNA and following the One-Color RNA Spike-In Kit protocol (Agilent 5188-5282) to monitor labelling reactions and microarray performance. Subsequently, samples were labelled using the Low Input Quick Amp Labeling kit (Agilent 5190-2305) by generating cDNA and then cRNA labelled with Cy-3 fluorescent dye. Samples were stored at -80°C until purification. Using the QIAGEN RNeasy Mini Kit (74136), cRNA samples were purified and eluted to a total volume of 30 µL with RNase-free water. A NanoDrop 2000™ spectrometer was used to measure sample purity and contamination (A260, 260/280), concentration, efficacy of the fluorescent dye incorporation (Concentration of Cy3), total cRNA yield (µg cRNA), and specific activity (Table S2.1). According to manufacturer recommendations, criteria for the specific activity (>6 pmol Cy3 per μg cRNA) and yield (>0.825 µg cRNA) should be met to ensure optimal microarray results.

*Table S2.1: Sample measurements of RNA concentration and associated A260/280 and A260/230 for each sample.*

| **Sample ID** | **Sample Type** | **NanoDrop™ Conc. (ng/µl)** | **A260/280** | **A260/230** |
| --- | --- | --- | --- | --- |
| **DEHP 1A** | CONTROL 4 | 244.4 | 2.11 | 2.15 |
| **DEHP 2A** | TCDD 25nM | 508.2 | 2.09 | 2.11 |
| **DEHP 3A** | 10μM | 237.2 | 2.11 | 2.07 |
| **DEHP 4A** | 100nM | 208.2 | 2.1 | 2.08 |
| **DEHP 5A** | 100pM | 248.9 | 2.11 | 2.13 |
| **DEHP 6A** | CONTROL 4 | 205.4 | 2.15 | 2.59 |
| **DEHP 7A** | TCDD 25nM | 423.2 | 2.13 | 2.33 |
| **DEHP 8A** | 10μM | 207.7 | 2.14 | 2.66 |
| **DEHP 9A** | 100nM | 199.2 | 2.14 | 2.75 |
| **DEHP 10A** | 100pM | 168.3 | 2.13 | 2.86 |
| **DEHP 1B** | CONTROL 4 | 235.1 | 2.13 | 2.11 |
| **DEHP 2B** | TCDD 25nM | 336.5 | 2.11 | 2.03 |
| **DEHP 3B** | 10μM | 288.2 | 2.12 | 2.11 |
| **DEHP 4B** | 100nM | 238.1 | 2.11 | 2.06 |
| **DEHP 5B** | 100pM | 277.1 | 2.11 | 2.11 |

Samples were hybridized using Agilent’s Gene Expression Hybridization Kit (Agilent 5188-5242) to Agilent SurePrint Zebrafish Gene Expression v3 4 x 44k Microarray Kit, design ID: 026437 (Agilent Technologies Inc., USA) following the manufacturer protocol. Hybridization assemblies were assessed for the presence of bubbles that could inhibit hybridization and then transferred to the hybridization oven (Agilent G2545A) and hybridized at 65°C for at least 17 hours. Samples were removed after hybridization and washed with Gene Expression wash buffers with added Triton X-102 (Agilent 5185-5975). Slides were inspected to ensure adequate washing and removal of debris that could interfere with reading, and were then placed in a SureScan slide holder (Agilent G4900-60035). Slide holders with microarrays were transferred to the Agilent SureScan Microarray Scanner™ (G2600, Agilent Technologies, Inc., USA) and analysed using GE 1200 one-colour protocol.

### Statistical Analysis

Scan results underwent quality control and background correction with Feature Extraction Software (Agilent version 11.5.1.1). Raw data were imported into GeneSpring software version 14.9 (Agilent) for further analysis. Data for GeneSpring™ was first normalized with quantile normalization and background corrected to the median of all samples. Quality control (outlier analysis) was completed using Principal Component Analysis (PCA), sample correlation analysis and flags on parameters generated by GeneSpring™. After preprocessing, a gene-wise analysis was set up to combine multiple probes coding for the same gene and so replicate genes are not presented individually. Non-detected and compromised probes, as well as control probes, were removed. Control probes are utilized for grid alignment and quality control and are not informative past this point. Data with coefficient variation of less than 30% were considered acceptable. Statistical analysis was performed using a Moderated T-Test with Westfall-Young multiple testing correction. A fold change cutoff of 1.5 and adjusted p-value cutoff of < 0.05 were applied. For the resultant DEGs from each analysis, Gene Ontology (GO) Analysis was performed using Entrez IDs and the clusterProfiler package in R, with molecular function, biological process, and cellular component subontologies.

For R:l*imma* (Smyth, 2004) analysis, scan results underwent quality control and background correction with Feature Extraction Software (Agilent version 11.5.1.1). Raw data were exported as .txt files and imported into R. Subsequently, data were analysed using the limma package with built-in analyses specific for One-Colour Agilent microarray data. Raw intensities were visually assessed and background correction was undertaken (“normexp”, offset = 50) as well as normalization (“quantile”). Probes coding for replicate genes were also averaged. Batch effects were visually analysed using multiple packages (*stats, tidyverse, ggplot2, pca3d*) but did not appear to contribute to the data variance and hence batch effect correction was not performed. Raw and pre-processed were compared using boxplots (*graphics*), hierarchical clustering, principal component analysis, log-intensity ratio to log-intensity average (MA plot), and expression densities between arrays to visualize the efficacy of the preprocessing methods. Array quality weights could not be estimated and therefore were not applied. Select quality control measures and visuals are presented in the Supplementary Materials. Statistical analysis was conducted using the linear modelling for microarray analysis. Multiple testing corrections were accounted for using the Benjamini-Hochberg method with an adjusted p-value cutoff of 0.05 and a log fold-change cutoff of 1.5. Resulting DEG lists were formed by filtering unannotated genes and mapped to known EntrezID genes. Pathway analysis was completed using Pathway Architect in GeneSpring for *Homo sapiens* using all annotations (RefSeq, Entrez Gene ID, Ensembl Gene ID, GenBank Accession, UniGene ID, TIGR ID) and WikiPathways, KEGG (where applicable), and BioCyc databases. Pathways with a p-value of less than 0.05 were considered significant.

## Metabolomics

### Sample Preparation

#### Polar Phase or Upper Phase

Briefly, 250 μL aliquot of each sample was transferred to a new Eppendorf tube after vortexing at 2000 rpm for 5 sec. Aliquots were dried under a gentle nitrogen flow using a Techne Sample concentrator (Time: 180 min, Temperature: 45 °C). Then, the samples were resuspended using 120 μL of solvent water/methanol (70:30), followed by vigorous vortexing for 5 sec at 2000 rpm. Samples were centrifuged at 15000 rpm for 10 min, and the supernatants were transferred to autosampler vials with inserts. A mixture of multiple internal standards was added to the solvent to assess system stability for each sample analysed (Table S2.2.). Internal standards were chosen based on their extracted ions, which should cover the retention time axis in both ionisation modes. The mixture of the internal standards was prepared as follows: 100 μL of each compound stored at 1000 ppm was added to 7100 μL of MeOH. Then 0.5 mL of the prepared mixture of internal standards was added to the 4.5 mL of the water/methanol in a ratio of 70:30 solvent. Pooled QC samples were prepared to condition the used analytical platform, perform intra-study reproducibility measurements and to correct mathematically systematic errors. For the preparation of the QC samples, equal volumes of 60 μL from each of the samples were mixed. The preparation of the QCs was the same as described above.

#### Non-polar Phase or Lower Phase

The sample preparation procedure was the same as described above. The only difference was the solvent used for the metabolites’ extraction. The samples were resuspended in 75 µL of a methanol/chloroform (3:1) solution containing the mixture of internal standards (Table S2.2).

Table S2.2. Internal standards were added to the samples to assess system stability for each sample analysed.

| Sample | Chemical Formula | MW | [M+H] + | [M-H]- |
| --- | --- | --- | --- | --- |
| L-tryptophan | C11 D5 H7 N2 O2 | 209.1213 | 210.1285 | 208.1136 |
| Fumarate | C4 D2 H2 O4 | 118.0235 | 119.0498 | 117.0162 |
| Citrate | C6H8O7 | 192.03 | 193.1304 | 191.1156 |
| Paracetamol | C8H9NO2 | 152.07 | 152.0706 | - |
| Caffeine | C8H10N4O2 | 195.08 | 195.0877 | - |
| Reserpine | C33H40N2O9 | 608.27 | 609.2807 | 607.2661 |
| Risperidone | C23H27FN4O2 | 410.21 | 411.2191 | - |
| Roxithromycin | C41H76N2O15 | 836.52 | 837.5318 | - |
| Caffeic acid | C9H8O4 | 180.04 | - | 179.035 |
| Tetrabromobisphenol A | C15H12Br4O2 | 539.75 | - | 542.7454 |

### Data Acquisition

#### Chromatographic separation

For the polar phase cell samples, a Fortis Speed Core pH+ C18 (2.1x 100 mm, 2.6 μm) from Fortis Technologies (United Kingdom) preceded by a filter column was used. The column was thermostat at 40^o^C. In both positive and negative mode, the flow rate was set at 400 μL/min, the injection volume at 5 μL, and the two mobile phases were 100% high-grade water with 0.1% formic acid (Mobile Phase A) and 100% methanol with 0.1% formic acid (Mobile Phase B). The following gradient was used: 0.1% B at 0 min, 0.1% B at 2 min, 25% B at 6 min, 80% B at 10 min, 90% B at 12 min, 99.9% B at 21 min, 99.9% B at 23 min, 0.1% B at 24 min and 0.1% B at 26 min.

For the non-polar phase cell samples, chromatographic separations were achieved using a Fortis HILIC (2.1x 100 mm, 2.6 μm) from Fortis Technologies (United Kingdom), preceded by a filter column. The column was thermostat at 35^o^C. The flow rate was 400μL/min, and the injection volume was 5 Μl in both negative and positive ionization mode. The mobile phases for positive ionization mode (PI) were ACN/Water (95:5) amended with 10 mM ammonium formate and 0.01% formic acid (Mobile Phase A), and ACN/Water (50:50) amended with 10 mM ammonium formate and 0.01% formic acid (Mobile Phase B). For negative ionization mode (NI), ACN/Water (95:5) amended with 10 mM Ammonium Acetate and 0.01% acetic acid (Mobile Phase A), and ACN/Water (50:50) amended with 10 mM ammonium acetate and 0.01% acetic acid (Mobile Phase B). The optimised gradient elution program was the same for both ionization modes: 0.1% B at 0 min, 0.1% B at 2 min, 25% B at 6 min, 80% B at 10 min, 90% B at 12 min, 99.9% B at 21 min, 99.9% B at 23 min, 0.1% B at 24 min and 0.1% B at 26 min.

#### Mass spectrometry

Cell samples were analysed with an Agilent 1290 infinity HPLC LC System coupled to an Agilent 6540 HRMS-QTOF/ LCMS system. The Q-TOF system was equipped with A Dual AJS ESI probe and operated in positive and negative modes using the full MS scan mode. The data were acquired between 50 and 1000 m/z at a scan rate of 1.5 spectra/s in centroid mode at a resolution of 40,000 FWHM. The source conditions were as follows: gas temperature 300 ^o^C, drying gas 7 L/min, nebulizer 50psig, fragmentor 250 V, skimmer 65 V, and capillary voltage 3500V or -3500V in positive or negative mode, respectively. References masses of 121.0509 (Purine) and 922.0098 (hexakis (1H, 1H, 3H-tetrafluoropropoxy) phosphazine) in PI and 112.9856 (TFA anion) and 980.0164 (Acetate) in NI were used for internal mass calibration during the runs. Two blank samples were injected at the beginning of each experiment followed by 10 runs of pooled QC sample to calibrate the column. The second blank sample contained the mixture of the internal standards. After the analysis of 3 problem samples, two blanks and one QC sample were analysed for the monitoring and the baseline stabilization of the LC–MS system (Figure S2.1).

.

Figure S2.1. Optimised analytical sequence for the LC-MS untargeted metabolomic analysis of cell samples. Solvent blank samples with and without standards (SDs) were analysed at the beginning, every 3 problem samples, and at the end of the sequence. Pooled quality control (QC) was injected 10 times before analysing problem samples to condition the column and evaluate system performance. The pooled QC was injected every 3 problem samples. Problem samples were analysed from lower to higher exposure doses to avoid contamination. Four injections of the pooled QC sample were added at the end of the batch for MS/MS data acquisition.

### Bioinformatics

#### Data Preprocessing

The UPLC-TOF-MS data of the analysed samples were collected with the Agilent MassHunter Workstation Data Acquisition Software v.B.06.01. Raw data generated from positive and negative ionization were pre-processed as two different experiments. The tool msConvert (Chambers et al., 2012; Kessner et al., 2008) was used to translate the vendor format (.d) data into the .mzML open format. Spectral processing was performed using the Bioconductor R - based packages XCMS v.3.10.1 (Smith et al., 2006) and CAMERA v.1.44.0 (Kuhl et al., 2012), running under R version 4.0.1 (http://http://www.r-project.org/). Briefly, after importing the raw data in .mzML format, an initial data inspection was performed using the base-peak chromatograms of the samples, boxplots representing the distribution of total ion currents per sample, and heatmaps of the QC and exposed samples. These plots were used to identify problematic or failing MS runs, obtain an initial evaluation of the introduced batch effects, and provide an initial overview of the sample grouping in the experiment. The next step in metabolomics LC-MS data pre-processing was the peak detection. For the chromatographic peak detection, the centWave algorithm developed by (Tautenhahn et al., 2008) was used. The functions of the IPO R-based package were implemented to optimise the ppm, peakwidth, mzdiff, and noise parameters (Libiseller et al., 2015), while the snthresh was calculated as follows: first, it was assumed that the x was the vector of intensity values of the actual regions of interest (ROIs), and xt the 10% trimmed x (5% of the smallest and 5% of the largest intensity values were discarded). Then, the baseline (BL) was defined as the mean of xt, and the noise level (NL) as the standard deviation of xt. The optimized XCMS parameters for all the performed experiments are listed in Table S2.3. The Obiwarp method was used for alignment (Prince & Marcotte, 2006). It is worth mentioning that the settings for each experiment were modified in accordance with the IPO optimisation results. The next step was to match detected chromatographic peaks across samples. The method to perform the correspondence in XCMS is the peak density, which combines chromatographic peaks depending on the density of peaks along the retention time axis within small slices along the m/z dimension (Richardson et al., 2008). Finally, the PerformPeakAnnotation function was used for isotope and adduct annotation.

Table S2.3. Optimised parameters resulting from the IPO R package.

| Experiment | ppm | peakwidth | sn | mzdiff |
| --- | --- | --- | --- | --- |
| RP (+) ESI | 24.5 | 11.2, 60) | 10 | 0.0045 |
| RP (-) ESI | 21.5 | (10.56, 30.5) | 5 | 0.00098 |
| HILIC (+) ESI | 26 | (13.6, 51.5) | 8 | 0.00395 |
| HILIC (-) ESI | 30 | (11.2, 51.5) | 6 | -0.001 |

#### Data pre-treatment

Τhe data matrix was refined using the 80% rule applied to quality control (QC) samples. Variables with more than 80% missing values (ion intensity = 0) were excluded (Wei et al., 2018). Detected peaks with 0% missing values in the solvent sample group were also removed, as these represent false positives due to contamination. Normalisation, mean centring, scaling, and log transformation were applied to approximate a Gaussian distribution and reduce systematic experimental errors. Detected peaks were normalized using multiple internal standards added to the QC samples, taking into account the proximity of metabolite peaks in both retention time and mass-to-charge ratio (m/z) (Katajamaa & Orešič, 2005). A baseline adjustment to the median of the QCs was implemented to ensure equal treatment of all compounds, regardless of intensity. Finally, a within-batch effects correction method developed by Brunius, et al. 2016 (Brunius et al., 2016), was employed to enhance peak detection, alignment, and quantification.

#### Metabolite annotation

The R package xMSAnnotator, developed by Uppal et al. (2017)(Uppal et al., 2017), was employed to perform the accurate mass searches in online compound databases (HMDB (Wishart et al., 2018), LipidMaps (Fahy et al., 2007), and KEGG (Kanehisa et al., 2017). The adduct list used for database matching comprised "M+2H", "M+H+NH4", "M+ACN+2H", "M+2ACN+2H", "M+H", "M+NH4", "M+Na", and "M+ACN+H" in positive ionization mode, and "M+ACN+Na", "M+2ACN+H", "2M+H", "2M+Na", "2M+ACN+H", "M-2H", "M-3H", "M-H2O-H", "M+Na-2H", "M-H", "M+Cl", "M+FA-H", "M+K-2H", and "2M-H" in negative ionization mode. Detected candidate metabolites were filtered by metabolite status (Detected). Confirmation of the selected biomarkers was achieved by comparing retention time and fragmentation patterns with authentic analytical standards from the in-house library or with MS/MS spectra available in databases such as HMDB, Metlin, and LipidMaps (Guijas et al., 2018).

#### Statistical analysis

The significantly different metabolites were identified using the ANOVA unequal-variance test in R, followed by the Benjamini-Hochberg false discovery rate (FDR) correction for multiple comparisons to minimise false positives. A p-value cut-off of 0.05 was considered. The Fold change was calculated as the ratio of control samples to exposed samples. The resulted metabolites were filtered considering a fold-change cut-off of 2.0

# EndoC-βH1

## Chemicals

Di(2-ethylhexyl) phthalate (DEHP, Cat No 36735 was purchased from Sigma-Aldrich (Saint Louis, MO, USA). DEHP was dissolved in dimethyl sulfoxide (DMSO) to prepare the stock solution. The amount of DMS) was maintained at less than 0.03%.

## EndoC-βH1 cell culture and treatment

EndoC-βH1 cells (Univercell Biosolutions (now named Human Cell Design), Paris, France) were cultured and passaged as previously described (Ravassard et al., 2011). Briefly, cells were cultured in DMEM containing 5.6 mM glucose, 2% BSA fraction V fatty acid free (Sigma-Aldrich, Saint Louis, MO, USA), 50 μM 2-β-mercaptoethanol (Gibco, Paisley, UK), 10 mM nicotinamide, 5.5 μg/mL human transferrin, 6.7 ng/mL sodium selenite (Sigma-Aldrich, Saint Louis, MO, USA), 100 units/mL penicillin and 100 μg/mL streptomycin (Thermo Fischer Scientific, Waltham, MA, USA). Cells were seeded at a density of 200 × 10^3^ cells/well cells/cm^2^ (for transcriptomic analysis) and at a density of 1× 10^6^ cells/well cells/cm^2^ (for metabolomic analysis) on ECM (1%) and fibronectin (2 μg/mL) (Sigma-Aldrich, Saint Louis, MO, USA) -coated plates, and cultured at 37 °C in 5% CO_2_. For treatment with DEHP, DMEM was replaced with DMEM without phenol red (Sigma-Aldrich, Saint Louis, MO, USA) and BSA was replaced by 2% charcoal dextran-treated FBS (HyClone, GE Healthcare Life Sciences, Logan, UT, USA). The selection of treatment durations and concentrations of EDCs tested (7 day treatment, vehicle control, 1 nM, 10 nM, and 100 nM) were selected as previously described by (Al-Abdulla et al., 2022).

## Transcriptomics

### RNA extraction

Briefly, cells were harvested and stored in 25 μL of RNAlater at -80°C until extraction. Total RNA was isolated using the RNeasy Mini Kit (Qiagen, Venlo, Netherlands) as per the supplier protocol. In general, RLT Buffer and 70% ethanol were added to each samples and filtered via centrifugation using the supplied RNeasy Mini spin column. Samples were clarified using one wash step with Buffer RW1 and two with Buffer RPE. The final sample was eluted using RNase-free water. RNA quantity and purity were assessed using a NanoDrop 2000 spectrometer (ThermoScientific) (Table S2.4).

*Table S2.4: Sample measurements of RNA concentration and associated A260/280 and A260/230 for each sample.*

| **Sample ID** | **Sample Type** | **NanoDrop™ Conc. (ng/µl)** | **A260/280** | **A260/230** |
| --- | --- | --- | --- | --- |
| **DEHP 1A** | Control | 53.7 | 2.01 | 129.31 |
| **DEHP 2** | 1 nM | 47.2 | 2.22 | 1.91 |
| **DEHP 3** | 10 nM | 38.1 | 2.29 | 1.32 |
| **DEHP 4** | 100 nM | 36 | 2.29 | 0.37 |
| **DEHP 5** | Control | 59.7 | 2.2 | 1.89 |
| **DEHP 6** | 1 nM | 79.8 | 2.16 | 2.13 |
| **DEHP 7** | 10 nM | 75.2 | 2.19 | 2.21 |
| **DEHP 8** | 100 nM | 125.7 | 2.08 | 2.11 |
| **DEHP 1B** | Control | 113.5 | 1.93 | 1.49 |
| **DEHP 10** | 1 nM | 24.5 | 2.08 | 1.18 |
| **DEHP 11** | 10 nM | 33.4 | 1.99 | 0.23 |
| **DEHP 12** | 100 nM | 20.3 | 1.85 | 1.08 |

### Microarray analysis and Data Acquisition

Gene expression profiles of treated samples were determined using Agilent SurePrint G3 human microarrays. The whole procedure followed the One-Color Microarray-Based Gene Expression Analysis (Low Input Quick Amp Labelling) Protocol version 6.9.1 provided by Agilent Technologies. To monitor labelling reactions and microarray performance, samples were spiked with the Agilent One Color RNA Spike-In mix (Agilent 5188-5282).

All steps followed the One-Color Microarray-Based Gene Expression Analysis (Low Input Quick Amp Labeling) Protocol version 6.9.1 supplied by Agilent Technologies. Briefly, samples were spiked with quality control Agilent RNA Spike-In mix in 1.5 µL of sample ranging from 10 to 200 ng total RNA and following the One-Color RNA Spike-In Kit protocol (Agilent 5188-5282) to monitor labeling reactions and microarray performance. Subsequently, samples were labeled using the Low Input Quick Amp Labeling kit (Agilent 5190-2305) by generating cDNA and then cRNA labeled with Cy-3 fluorescent dye. Samples were stored at -80°C until purification.  Using the QIAGEN RNeasy Mini Kit (74136), cRNA samples were purified and eluted to a total volume of 30 µL with RNase-free water. A NanoDrop 2000™ spectrometer was used to measure sample purity and contamination (A260, 260/280), concentration, efficacy of the fluorescent dye incorporation (Concentration of Cy3), total cRNA yield (µg cRNA), and specific activity. According to the manufacturer's recommendations, the criteria for the specific activity (>6 pmol Cy3 per μg cRNA) and yield (>0.825 µg cRNA) should be met to ensure optimal microarray results.

Samples were hybridized using Agilent’s Gene Expression Hybridization Kit to Agilent SurePrint Human Gene Expression v3 8 x 60 k Microarray Kit, design ID: 072363 (Agilent Technologies Inc., CA) following the manufacturer protocol. Hybridization assemblies were assessed for the presence of bubbles that could inhibit hybridization and then transferred to the hybridization oven (Agilent G2545A) and hybridized at 65°C for ~17 hours. Samples were removed after hybridization and washed with Gene Expression wash buffers with added Triton X-102 (Agilent 5185-5975). Slides were inspected to ensure adequate washing and removal of debris that could interfere with reading and placed in a SureScan slide holder (Agilent G4900-60035). Slide holders with microarrays were transferred to the Agilent SureScan Microarray Scanner™ (G2600, Agilent Technologies, Inc., CA) and analyzed using GE 1200 one-color protocol.

### Data Preprocessing and Statistical Analysis

After scanning, results underwent quality control and background correction with Feature Extraction Software (Agilent version 11.5.1.1). Raw data were exported as .txt files and imported into R. Subsequently, data were analyzed using the *limma* package with built-in analyses specific for One-Colour Agilent microarray data. Raw intensitites were visually assessed and background correction was undertaken (“normexp”, offset = 50) as well as normalization (“quantile”). Probes coding for replicate genes were averaged. Batch effects were visually analyzed using multiple packages (stats, tidyverse, ggplot2, pca3d). Experimental replicate appeared to be the most significant factor that contributed a noticeable degree of variance to the data and therefore, this variable was used for batch effect estimations and removal, where possible. Raw and pre-processed data were compared using boxplots (graphics), hierarchical clustering, principal component analysis, log-intensity ratio to log-intensity average (MA plot), and expression densities between arrays to visualize the efficacy of the preprocessing methods. Relative quality weights for each array were estimated and applied based on magnitude of residuals.

Statistical analysis was conducted using the linear modeling for microarray (*limma)* analysis method (Ritchie et al., 2015). *Limma* utilizes linear model fitting to control for study design and implements empirical Bayesian functions to calculate the gene-wise test statistic (moderated t-test statistic). This method provides more statistical power as it borrows information from inter-gene comparisons*.* Next, the analysis was set up for all relevant comparisons using design and contrast matrices. Due to a low sample size, a robust regression was performed to address some outliers that remained after pre-processing and to provide more stable and accurate coefficient estimates. Multiple testing corrections were applied using the Benjamini-Hochberg method with an FDR cutoff of 0.05.

## Metabolomics

### Sample Preparation

First, 200μL from each sample were transferred to a new Eppendorf. Aliquots were dried under a gentle nitrogen flow using a Techne Sample concentrator (Time: 180 min, Temperature: 45 °C). Then, the samples were resuspended using 100 μL of solvent water/methanol (70:30), followed by vigorous vortex for 5 sec at 2000 rpm. The samples were centrifuged at 15000 rpm for 10 min, and the supernatants were transferred to autosampler vials with inserts. QC samples were prepared by transferring equal amounts from each sample to Eppendorf tubes, followed by vortexing at 2000 rpm for 5 sec. A mixture of multiple internal standards was added to the QC samples to assess system stability for each sample analysed. A similar procedure was followed for the non-polar samples, with the only difference in the reconstruction step, where a solvent of 75 μL of ACN/CHCl_3_ (70:30) was used.

### Data Acquisition

**Chromatographic separation:** For the analysis of polar samples, a Fortis Speed Core pH+ C18 (2.1x 100 mm, 2.6 μm) from Fortis Technologies (United Kingdom) preceded by a filter column was used. The column was thermostat at 40^o^C. In both positive and negative ionisation mode, the flow rate was set at 400 μL/min, the injection volume at 5μL, and the two mobile phases were 100% high-grade water with 0.1% formic acid (Mobile Phase A) and 100% methanol with 0.1% formic acid (Mobile Phase B). The following gradient was used: 0.1% B at 0 min, 0.1% B at 2 min, 25% B at 6 min, 80% B at 10 min, 90% B at 12 min, 99.9% B at 21 min, 99.9% B at 23 min, 0.1% B at 24 min and 0.1% B at 26 min. Beyond the full MS spectra for all the samples, 2 MS/MS spectra were recorded at the end of the run. (Table 3).

A Fortis HILIC (2.1x 100 mm, 2.6 μm) from Fortis Technologies (United Kingdom) preceded by a filter column was used for the analysis of the non-polar samples. The column was thermostated at 35 oC with a flow rate of 400 μL/min. The mobile phases for positive ionization mode were 95% ACN / 5% Water amended with 10 mM Ammonium Formate and 0.01% formic acid (solvent A) and 50% ACN / 50% Water, amended with 10 mM Ammonium Formate and 0.01% formic acid (solvent B). For negative ionization mode, was 95% ACN / 5% Water amended with 10 mM Ammonium Acetate and 0.01% acetic acid (solvent A) and 50% ACN / 50% Water, amended with 10 mM Ammonium Acetate and 0.01% acetic acid (solvent B). The adopted gradient elution program was the same for both ionization modes, starting with 0% B with a flow rate of 0.400 mL/min for 2 min, and then increasing to 100 % B in 17 min (flow rate 0.400 mL/min). Then it kept constant for 5 min (flow rate 0.400 mL/min), then initial conditions were restored within 2 min, kept for 2 min and then the flow rate decreased at 0.400 mL/min. The injection volume is set up to 5 μL. Beyond the full MS spectra for all the samples, 3 MS/MS spectra were recorded at the end of the run (Fig.S2.2.).

**Mass spectrometry:** Pancreatic cell samples were analysed with an Agilent 1290 infinity HPLC LC System coupled to an Agilent 6540 QTOF. The Q-TOF system was equipped with a Dual AJS ESI probe and operated in positive and negative modes in full MS scan mode. The data were acquired between 50 and 1700 m/z at a scan rate of 1.5 spectra/s in centroid mode at a resolution of 40,000 FWHM. The source conditions were as follows: gas temperature 300^o^C, drying gas 7 L/min, nebulizer 50psig, fragmentor 250 V, skimmer 65 V, and capillary voltage 3500V or -3500V in positive or negative mode, respectively. Reference masses 121.0509 (Purine) and 922.0098 (hexakis (1H, 1H, 3H-tetrafluoropropoxy) phosphazine, HP-0921) in positive mode and 112.9856 (TFA anion) and 980.0164 [HP-0921 (+ acetate)] in negative mode were used for internal mass calibration during the runs.

Figure S2.2. Sequence followed for the sample analysis for both ionizations. The order followed for the analysis of the problem samples was based on the concentrations of each exposure compound, to avoid any contamination during the analyses.

### Bioinformatics

The bioinformatics pipeline used for metabolomics analysis followed the same process as described previously with liver samples. The optimised parameters for peak detection, obtained using the R package IPO, are listed in the table (Table S2.5.).

Table S2.5 . Optimised parameters resulting from the IPO R package.

| **Experiment** | **ppm** | **peakwidth** | **snthresh** | **mzdiff** |
| --- | --- | --- | --- | --- |
| RP (+) ESI | 26 | (9.6, 50) | 13 | -0.00045 |
| RP (-) ESI | 30 | (10.4, 51) | 3 | 0.0155 |
| HILIC (+) ESI | 21.5 | (9.76, 65) | 11 | 0.00835 |
| HILIC (-) ESI | 24.5 | (9.6, 44) | 6 | 0.0155 |

# D. rerio 5dpf

### Chemicals

Reference DEHP was purchased from Sigma Aldrich. LC/MS grade extraction solvents, including methanol, water, acetonitrile, and CH_2_Cl_2_ were employed. Alongside LC/MS grade Formic Acid and Acetic Acid from Sigma Aldrich.

### Fish samples treatment

A new reference curve was not made for the fish samples, although an ACN solvent was used. A blank sample (DMSO) spiked with DEHP and DBP (as internal standard) was used for subsequent quantifications. Fish were homogenized with the Tissue master for complete extraction and homogenization. Then the extraction vial was centrifuged. From that an amount was taken into a vial insert as is undiluted (D1) or a 1/10 dilution with ACN was made into a vial (D2). D1 samples were the ones with expected lower or zero concentrations whereas the D2 coded samples were the ones that were expected to be found with higher concentrations.

## Transcriptomics analysis

### RNA extraction

Approximately 15 zebrafish larvae from each treatment group were received from the Institute of Research on Health and Environment (IRSET U1085, Rennes France) and stored at -80°C until further processing. All experiments were performed in quadruplicates. For RNA extractions, samples were first homogonized in TRIzol reagent™ (Invitrogen, 15596026) and then extracted following Invitrogen PureLink™ RNA Minikit (12183018A) and following manufacturer instructions. RNA product was quantified using NanoDrop 2000 spectrometer (ThermoScientific) equipment to measure extraction success and sample concentration and integrity (Table S2.6). Samples were stored at -80°C until microarray analysis.

Table S2.6: Sample measurements of RNA Concentration and associated A260/280 and A260/230.

| **Sample ID** | **Sample Type** | **NanoDrop™ Conc. (ng/µl)** | **A260/280** | **A260/230** |
| --- | --- | --- | --- | --- |
| **5.2** | DMSO | 350.8 | 2.02 | 2.21 |
| **5.5** | DEHP 10μM | 423.9 | 2.03 | 2.16 |
| **5.8** | DEHP 0.025μM | 461.5 | 2.03 | 2.11 |
| **6.2** | DMSO | 236.4 | 2.11 | 1.95 |
| **6.5** | DEHP 10μM | 333.9 | 2.12 | 1.99 |
| **7.2** | DMSO | 217.7 | 2.12 | 1.95 |
| **7.5** | DEHP 10μM | 204.2 | 2.11 | 1.68 |
| **7.8** | DEHP 0.025μM | 271.1 | 2.10 | 1.71 |
| **8.2** | DMSO | 412.8 | 2.10 | 2.16 |
| **8.5** | DEHP 10μM | 501.0 | 2.11 | 1.57 |
| **8.8** | DEHP 0.025μM | 542.6 | 2.08 | 2.02 |

### Microarray analysis

All steps followed the One-Color Microarray-Based Gene Expression Analysis (Low Input Quick Amp Labeling) Protocol version 6.9.1 supplied by Agilent Technologies. Briefly, samples were spiked with quality control Agilent RNA Spike-In mix in 1.5 µL of sample ranging from 10 to 200 ng total RNA and following the One-Color RNA Spike-In Kit protocol (Agilent 5188-5282) to monitor labeling reactions and microarray performance. Subsequently, samples were labeled using the Low Input Quick Amp Labeling kit (Agilent 5190-2305) by generating cDNA and then cRNA labeled with Cy-3 fluorescent dye. Samples were stored at -80°C until purification. Using the QIAGEN RNeasy Mini Kit (74136), cRNA samples were purified and eluted to a total volume of 30 µL with RNase-free water. A NanoDrop 2000™ spectrometer was used to measure sample purity and contamination (A260/230, 260/280), concentration, efficacy of the fluorescent dye incorporation (Concentration of Cy3), total cRNA yield (µg cRNA), and specific activity. According to the manufacturer's recommendations, the criteria for the specific activity (>6 pmol Cy3 per μg cRNA) and yield (>0.825 µg cRNA) should be met to ensure optimal microarray results.

Samples were hybridized using Agilent’s Gene Expression Hybridization Kit (Agilent 5188-5242) to Agilent SurePrint Zebrafish Gene Expression v3 4 x 44k Microarray Kit, design ID: 026437 (Agilent Technologies Inc., CA) following the manufacturer protocol. Hybridization assemblies were assessed for the presence of bubbles that could inhibit hybridization and then transferred to the hybridization oven (Agilent G2545A) and hybridized at 65°C for at least 17 hours. Samples were removed after hybridization and washed with Gene Expression wash buffers with added Triton X-102 (Agilent 5185-5975). Slides were inspected to ensure adequate washing and removal of debris that could interfere with reading, and were then placed in a SureScan slide holder (Agilent G4900-60035). Slide holders with microarrays were transferred to the Agilent SureScan Microarray Scanner™ (G2600, Agilent Technologies, Inc., CA) and analyzed using GE 1200 one-color protocol.

### Statistical Analysis

Scan results underwent quality control and background correction with Feature Extraction Software (Agilent version 11.5.1.1). For R: limma analysis, scan results underwent quality control and background correction with Feature Extraction Software (Agilent version 11.5.1.1). Raw data were exported as .txt files and imported into R. In R, data were analyzed using the limma package with built-in analyses specific for One-Colour Agilent microarray data. Raw intensitites were visually assessed and background correction was undertaken (“normexp”, offset = 50) as well as normalization (“quantile”). Probes coding for replicate genes were also averaged. Batch effects were visually analyzed using multiple packages (*stats, tidyverse, ggplot2, pca3d*) but did not appear to contribute to the data variance and hence batch effect correction was not performed. Raw and preprocessed were compared using boxplots (*graphics*), hierarchical clustering, principal component analysis, log-intensity ratio to log-intensity average (MA plot), and expression densities between arrays to visualize the efficacy of the preprocessing methods. Array quality weights could not be estimated and therefore were not applied. Select quality control measures and visuals are presented in the Supplementary Materials. Statistical analysis was conducted using the linear modeling for microarray analysis. Multiple testing corrections were accounted for using the Benjamini-Hochberg method with an adjusted p-value cutoff of 0.05 and a log fold change cutoff of 1.3. Resulting DEG lists were tidied by filtering unannotated genes and mapped to known EntrezID genes.

Additionally, raw data were imported into Genespring software version 14.9 (Agilent) for further analysis. Data for the GeneSpring™ was first batch corrected using a parametric ComBat algorithm with quantile normalization in the R Script Editor extension. General overlap of the red and black lines of the density plots generated from the algorithm indicates that batch correction analysis can be completed as parametric, as the difference between a parametric and non-parametric correction will have a low level of deviation (<5%). Following batch correction, background correction was applied to the median of all samples. Quality control (outlier analysis) was completed using Principal Component Analysis (PCA), sample correlation analysis and flags on parameters generated by GeneSpring™. After preprocessing, a gene-wise analysis was set up to combine multiple probes coding for the same gene and so replicate genes are not presented individually. Non-detected and compromised probes, as well as control probes, were removed. Control probes are utilized for grid alignment and quality control and are not informative past this point. Data with coefficient variation of less than 30% were considerd acceptable. Due to the fact that statistical analysis didn’t result any DEGs, a fold change analysis with a cutoff of 1.3 was performed. Because no individual transcript met the BH-FDR threshold of 0.05, an absolute log₂ fold-change threshold of |log₂FC| ≥ 1.3 was applied solely as a descriptive, hypothesis-generating filter; the retained transcripts were not considered statistically significant or used as independent inferential evidence, and pathway-level interpretation relied on the FDR-controlled set-level analysis reported in Supplementary Material 3.

## Metabolomic Analysis

### Sample Preparation

Briefly, 500 μl of prechilled MeOH/H_2_O (v:v 1:1) were added to each sample (each Eppendorf contained 40 larvae). The frozen embryos were homogenized using the electric homogenizer OMNI Tissue Master followed by gentle vortex. Samples were centrifuged at 15000 rpm for 10 min at 4^o^C, and the supernatants were transferred to new Eppendorfs, while the pellets will be used for the HILIC.

The aqueous extracts were dried under a gentle nitrogen flow using a Techne Sample concentrator (Time: 180 min, Temperature: 45 °C). Then, the samples were resuspended using 100 μl of solvent water/methanol (80:20), followed by vigorous vortex for 5 sec at 2000 rpm. Finally, the samples were transferred to autosampler vials with inserts.

For the preparation of the organic extracts, which will be analysed using a HILIC column, 500 μl of CH_2_Cl_2_/MeOH (v:v 3:1) were added to the pellet created after the first centrifugation described above. The pellet was dissolved using the electric homogenizer OMNI Tissue Master followed by gentle vortex. Samples were centrifuged at 15000 rpm for 10 min at 4^o^C, and the supernatants were transferred to autosampler vials.

For both analyses pooled QC samples were prepared to condition the used analytical platform, perform intra-study reproducibility measurements and to correct mathematically systematic errors. For the preparation of the QC samples equal volumes of 70 μL from each of the samples were mixed. The preparation of the QCs was the same as described above. Internal standards were added to the QC samples to assess system stability for each sample analysed (Table S2.7.).

Table S2.7: List of the internal standards used to assess system stability. The molecular formula, mass, and the respective masses of the adduct ions with the addition or removal of a hydrogen are included.

| Internal Standards | Molecular formula | Mass | [M+H] + | [M-H] - |
| --- | --- | --- | --- | --- |
| Fumarate | C_4_ D_2_ H_2_ O_4_ | 118.0235 | 119.0498 | 117.016 |
| Paracetamol | C_8_H_9_NO_2_ | 151.163 | 152.0706 | - |
| caffeic | C_9_H_8_O_4_ | 180.16 | - | 179.035 |
| Citrate | C_4_ [^13^C]_2_ O_7_ H_8_ | 194.0337 | 195.041 | 193.026 |
| Tryptophan | C_11_ D_5_ H_7_ N_2_ O_2_ | 209.1213 | 210.1285 | 208.114 |
| Resperidone | C_23_H_27_FN_4_O_2_ | 410.5 | 411.2191 | - |
| Tetrabromobisphenol | C_15_H_12_Br_4_O_2_ | 543.9 | - | 542.746 |
| Roxithomicine | C_41_H_76_N_2_O_15_ | 837.047 | 837.5318 | - |
| Reserpine | C_33_H_40_N_2_O_9_ | 608.68 | 609.2807 | 607.266 |
| Caffeine | C_8_H_10_N_4_O_2_ | 194.19 | 195.0877 | - |

.

### Sample Data Acquisition

**Chromatographic separation**: A Fortis Speed Core pH+ C18 (2.1x 100 mm, 2.6 μm) from Fortis Technologies (United Kingdom) preceded by a filter column was used for the analysis of polar samples. The column was thermostat at 40 ^o^C. In both positive and negative mode, the flow rate was set at 200 μl/min, the injection volume at 5 μL, and the two mobile phases were high-grade MeOH in H_2_O with 0.1% formic acid (Mobile Phase A) and 100% methanol with 0.1% formic acid (Mobile Phase B). The following gradient was used: 1% B at 0 min, 1% B at 1 min, 39% B at 3 min, 99.9% B at 14 min, 99.9% B at 16 min, 1% B at 16.01 min, 1% B at 19 min, 1% B at 22 min.

For the organic extracts, chromatographic separations were achieved using a Fortis HILIC (2.1x 100 mm, 2.6 μm) from Fortis Technologies (United Kingdom) preceded by a filter column is used. The column was thermostat at 40^o^C. The flow rate was 200 μl/min and the injection volume was 5 μL in both negative and positive ionization modes. The mobile phases for positive ionization mode (PI) were ACN/Water (95:5) amended with 10 mM ammonium formate and 0.01% formic acid (Mobile Phase A), and ACN/Water (50:50) amended with 10 mM ammonium formate and 0.01% formic acid (Mobile Phase B). For negative ionization mode (NI), ACN/Water (95:5) amended with 10 mM Ammonium Acetate and 0.01% acetic acid (Mobile Phase A), and ACN/Water (50:50) amended with 10 mM ammonium acetate and 0.01% acetic acid (Mobile Phase B). The optimised gradient elution program was the same for both ionization modes: 100% B at 0 min, 100% B at 2 min, 5% B at 12 min, 5% B at 17 min, 100% B at 17.01 min, 100% B at 25 min.

**Mass spectrometry:** Samples were analysed with an Agilent 1290 infinity HPLC LC System coupled to an Agilent 6540 HRMS-QTOF/ LCMS system. The Q-TOF system was equipped with A Dual AJS ESI probe and operated in positive and negative modes using the full MS scan mode. The data were acquired between 50 and 1000 m/z at a scan rate of 1.0 spectra/s in centroid mode at a resolution of 40,000 FWHM. The source conditions were as follows: gas temperature 330^o^C, drying gas 8 L/min, nebulizer 40psig, fragmentor 150 V, and capillary voltage 3500V or -3500V in positive or negative mode, respectively. Reference masses of 121.0509 (purine) and 922.0098 (hexakis(1H, 1H, 3H-tetrafluoropropoxy) phosphazine, HP-0921) in positive and 112.9856 (TFA anion) and 980.0164 (acetate) in negative mode were used for internal mass calibration during the runs. Two blank samples were injected at the beginning of each experiment followed by 10 runs of pooled QC sample to calibrate the column. The second blank sample also contained the mixture of the internal standards. After the analysis of four randomized problem samples (one sample from each condition was included in each batch), two blanks and one QC samples were analysed for the monitoring and the baseline stabilization of the LC–MS system (Figure S2.3).

Figure S2.3.: Sequence followed for the sample analysis for both ionizations. The order for analysing the problem samples was based on the concentrations of each exposure compound to avoid contamination during analysis.

### Bioinformatics

The bioinformatics pipeline used for metabolomics analysis followed the same process as described previously with liver samples. The optimised parameters for peak detection, obtained using the R package IPO, are listed in 8.

Table S28: Optimized parameters for the chromatographic peak detection step using the CentWave algorithm.

| Experiment | ppm | peakwidth | snthresh | mzdiff |
| --- | --- | --- | --- | --- |
| RP (+) ESI | 21.5 | (16, 62) | 8 | 0.00285 |
| RP (-) ESI | 29.75 | (16, 75.5) | 9.5 | -0.0065 |
| HILIC (+) ESI | 30 | (15.2, 41) | 7.8 | -0.00045 |
| HILIC (-) ESI | 30 | (15.1, 89.3) | 13 | 0.021946 |

## References

Al-Abdulla, R., Ferrero, H., Soriano, S., Boronat-Belda, T., & Alonso-Magdalena, P. (2022). Screening of Relevant Metabolism-Disrupting Chemicals on Pancreatic β-Cells: Evaluation of Murine and Human In Vitro Models. *International Journal of Molecular Sciences*, *23*(8), 1–29. https://doi.org/10.3390/ijms23084182

Brunius, C., Shi, L., & Landberg, R. (2016). Large-scale untargeted LC-MS metabolomics data correction using between-batch  feature alignment and cluster-based within-batch signal intensity drift correction. *Metabolomics : Official Journal of the Metabolomic Society*, *12*(11), 173. https://doi.org/10.1007/s11306-016-1124-4

Chambers, M. C., Maclean, B., Burke, R., Amodei, D., Ruderman, D. L., Neumann, S., Gatto, L., Fischer, B., Pratt, B., Egertson, J., Hoff, K., Kessner, D., Tasman, N., Shulman, N., Frewen, B., Baker, T. A., Brusniak, M.-Y., Paulse, C., Creasy, D., … Mallick, P. (2012). A cross-platform toolkit for mass spectrometry and proteomics. In *Nature biotechnology* (Vol. 30, Number 10, pp. 918–920). https://doi.org/10.1038/nbt.2377

Fahy, E., Sud, M., Cotter, D., & Subramaniam, S. (2007). LIPID MAPS online tools for lipid research. *Nucleic Acids Research*, *35*(suppl_2), W606–W612. https://doi.org/10.1093/nar/gkm324

Guijas, C., Montenegro-Burke, J. R., Domingo-Almenara, X., Palermo, A., Warth, B., Hermann, G., Koellensperger, G., Huan, T., Uritboonthai, W., Aisporna, A. E., Wolan, D. W., Spilker, M. E., Benton, H. P., & Siuzdak, G. (2018). METLIN: A Technology Platform for Identifying Knowns and Unknowns. *Analytical Chemistry*, *90*(5), 3156–3164. https://doi.org/10.1021/acs.analchem.7b04424

Kanehisa, M., Furumichi, M., Tanabe, M., Sato, Y., & Morishima, K. (2017). KEGG: New perspectives on genomes, pathways, diseases and drugs. *Nucleic Acids Research*, *45*(D1), D353–D361. https://doi.org/10.1093/nar/gkw1092

Katajamaa, M., & Orešič, M. (2005). Processing methods for differential analysis of LC/MS profile data. *BMC Bioinformatics*, *6*(1), 179. https://doi.org/10.1186/1471-2105-6-179

Kessner, D., Chambers, M., Burke, R., Agus, D., & Mallick, P. (2008). ProteoWizard: open source software for rapid proteomics tools development. *Bioinformatics*, *24*(21), 2534–2536. https://doi.org/10.1093/bioinformatics/btn323

Kuhl, C., Tautenhahn, R., Böttcher, C., Larson, T. R., & Neumann, S. (2012). CAMERA: An Integrated Strategy for Compound Spectra Extraction and Annotation of Liquid Chromatography/Mass Spectrometry Data Sets. *Analytical Chemistry*, *84*(1), 283–289. https://doi.org/10.1021/ac202450g

Libiseller, G., Dvorzak, M., Kleb, U., Gander, E., Eisenberg, T., Madeo, F., Neumann, S., Trausinger, G., Sinner, F., Pieber, T., & Magnes, C. (2015). IPO: a tool for automated optimization of XCMS parameters. *BMC Bioinformatics*, *16*(1), 118. https://doi.org/10.1186/s12859-015-0562-8

Prince, J. T., & Marcotte, E. M. (2006). Chromatographic alignment of ESI-LC-MS proteomics data sets by ordered bijective  interpolated warping. *Analytical Chemistry*, *78*(17), 6140–6152. https://doi.org/10.1021/ac0605344

Ravassard, P., Hazhouz, Y., Pechberty, S., Bricout-Neveu, E., Armanet, M., Czernichow, P., & Scharfmann, R. (2011). A genetically engineered human pancreatic β cell line exhibiting glucose-inducible insulin secretion. *The Journal of Clinical Investigation*, *121*(9), 3589–3597. https://doi.org/10.1172/JCI58447

Richardson, A. D., Yang, C., Osterman, A., & Smith, J. W. (2008). Central carbon metabolism in the progression of mammary carcinoma. *Breast Cancer Research and Treatment*, *110*(2), 297–307. https://doi.org/10.1007/s10549-007-9732-3

Ritchie, M. E., Phipson, B., Wu, D., Hu, Y., Law, C. W., Shi, W., & Smyth, G. K. (2015). *limma powers differential expression analyses for RNA-sequencing and microarray studies*. *43*(7). https://doi.org/10.1093/nar/gkv007

Smith, C. A., Want, E. J., O’Maille, G., Abagyan, R., & Siuzdak, G. (2006). XCMS:  Processing Mass Spectrometry Data for Metabolite Profiling Using Nonlinear Peak Alignment, Matching, and Identification. *Analytical Chemistry*, *78*(3), 779–787. https://doi.org/10.1021/ac051437y

Smyth, G. K. (2004). Linear models and empirical bayes methods for assessing differential expression in microarray experiments. *Statistical Applications in Genetics and Molecular Biology*, *3*(1). https://doi.org/10.2202/1544-6115.1027

Tautenhahn, R., Böttcher, C., & Neumann, S. (2008). Highly sensitive feature detection for high resolution LC/MS. *BMC Bioinformatics*, *9*(1), 504. https://doi.org/10.1186/1471-2105-9-504

Uppal, K., Walker, D. I., & Jones, D. P. (2017). xMSannotator: An R Package for Network-Based Annotation of High-Resolution  Metabolomics Data. *Analytical Chemistry*, *89*(2), 1063–1067. https://doi.org/10.1021/acs.analchem.6b01214

Wei, R., Wang, J., Su, M., Jia, E., Chen, S., Chen, T., & Ni, Y. (2018). Missing Value Imputation Approach for Mass Spectrometry-based Metabolomics Data. *Scientific Reports*, *8*(1), 663. https://doi.org/10.1038/s41598-017-19120-0

Wishart, D. S., Feunang, Y. D., Marcu, A., Guo, A. C., Liang, K., Vázquez-Fresno, R., Sajed, T., Johnson, D., Li, C., Karu, N., Sayeeda, Z., Lo, E., Assempour, N., Berjanskii, M., Singhal, S., Arndt, D., Liang, Y., Badran, H., Grant, J., … Scalbert, A. (2018). HMDB 4.0: The human metabolome database for 2018. *Nucleic Acids Research*, *46*(D1), D608–D617. https://doi.org/10.1093/nar/gkx1089
