# Supplementary material for: Mapping DEHP-induced metabolic dysregulation through multi-omics and systems biology: an adverse outcome pathway network approach
Source: Front Toxicol. 2026 Sep 30;8:1930835. doi: 10.3389/ftox.2026.1930835 (PMC13661021; doi:10.3389/ftox.2026.1930835)
Supplement: Supplementary file 3 [file Supplementaryfile3.docx]

# Supplementary Material 3

**FDR-Controlled Sensitivity Reanalysis and Cross-System Gene-Set Comparison**

*Manuscript: Mapping DEHP-Induced Metabolic Dysregulation Through Multi-Omics and Systems Biology: An Adverse Outcome Pathway Network Approach*

Manuscript ID 1930835 · Frontiers in Toxicology · Original Research

Data: doi:10.5281/zenodo.20376941 · Analysis code: Section 9 of this document

## 1. Purpose and scope

This Supplementary Material reports an FDR-controlled sensitivity analysis, in which the feature-selection strategy used in the main analysis is re-examined under strict false-discovery-rate (FDR) control. The main text applies a dual-criterion filter in which a transcript or metabolite is retained on the magnitude of its response, with or without accompanying statistical significance. This analysis examines whether the key events and key event relationships of the adverse outcome pathway network remain supported when only statistically supported features are considered.

To answer this we returned to the deposited per-sample expression matrices and repeated the entire differential-expression analysis under Benjamini-Hochberg (BH) control, at three levels: the individual transcript, the gene set, and the joint transcriptomic-metabolomic pathway analysis. This document reports the outcome in full, including the attrition of features at each level of stringency, and it does so whether or not the outcome favours the original analysis. The principal finding is that it does not favour it at the level of the individual transcript but does favour it at the pathway level. The evidential interpretation therefore rests primarily on pathway-level statistics.

## 2. Data and methods

### 2.1 Input data

Normalised log₂ expression matrices for all three experimental systems were taken from the deposited dataset (doi:10.5281/zenodo.20376941, file OBERON_DEHP_Metbolites_genes_summary.xlsx), which contains the per-sample values underlying the analyses reported in the main text. The matrices comprise 21,718 annotated transcripts for the 3D HepaRG system, 15,532 for EndoC-βH1 pancreatic β-cells and 14,685 for zebrafish larvae. Probes mapping to the same gene had already been averaged during the original preprocessing, so each row corresponds to one Entrez gene identifier.

Eight DEHP contrasts were refitted, each against its matched vehicle control: HepaRG 3D at 100 pM, 100 nM and 10 µM; EndoC-βH1 at 1, 10 and 100 nM; and zebrafish larvae at 25 nM and 10 µM. The human cell systems provide three biological replicates per condition and the zebrafish experiment four, giving the replicate structure stated in Supplementary Material 2.

### 2.2 Differential expression

Each contrast was refitted in limma (version 3.62.1) using linear models with robust empirical-Bayes moderation and an intensity-dependent prior variance trend (eBayes with robust = TRUE and trend = TRUE). Because control and treated arms were processed as matched experimental replicates, and because Supplementary Material 2 identifies experimental replicate as the dominant component of variance in these datasets, replicate was included as a blocking factor in the design matrix. This is a material methodological point: omitting the block leaves replicate-level offsets in the residual variance and, in the zebrafish data in particular, produces thousands of apparently FDR-significant transcripts at very small effect sizes. The blocked analysis reported here is the appropriate one and is also the analysis consistent with the statement in Supplementary Material 2 that no individual zebrafish transcript survived correction.

Benjamini-Hochberg adjustment was applied within each contrast, over all genes tested in that contrast. No cross-contrast pooling of p-values was performed at the gene level.

### 2.3 Competitive gene-set testing

Set-level testing used CAMERA, which tests whether the genes of a set are differentially expressed relative to all other genes in the same experiment while accounting for inter-gene correlation. A fixed inter-gene correlation of 0.01 was used throughout, the conventional default when a correlation cannot be estimated reliably from a small number of replicates. CAMERA is appropriate at this sample size precisely because it aggregates evidence across the tens to hundreds of genes constituting a pathway rather than relying on any single gene reaching significance.

Gene sets were the KEGG pathway definitions carrying the key events of the network, retrieved for Homo sapiens and Danio rerio from the MSigDB C2:CP:KEGG_LEGACY collection (release 2026.1.Hs). Nine pathways were tested in each of the eight contrasts, giving 72 model-by-dose-by-pathway tests. BH correction was applied both within each contrast and globally across the full family of 72 tests; both are reported.

Note on gene-set provenance: the main-text analysis used KEGG definitions retrieved directly from the KEGG database at the time of analysis, whereas the reanalysis reported here used the MSigDB distribution of the same KEGG pathways. The two sources differ slightly in pathway membership and in pathway naming (for example, MSigDB provides a single "fatty acid metabolism" set rather than separate degradation and biosynthesis sets). Absolute z-scores and adjusted p-values therefore differ in detail from those quoted in the main text, while the qualitative pattern — which pathways are significant, in which systems, and in which direction — is unchanged. Users reproducing this analysis against live KEGG should expect the same pattern with slightly different numerical values.

### 2.4 Directional pathway score

CAMERA returns a p-value and a direction but not a magnitude that can be compared across contrasts. To provide one, a directional pathway score was computed for each pathway in each contrast as the standardised mean moderated t-statistic of its member genes, z = √k · mean(t_set) / sd(t_all), where k is the number of set members represented in that contrast. Positive values denote coordinated induction and negative values coordinated suppression. This score is the quantity displayed in the cross-system comparison of the main text and in Figure S3.3 below.

### 2.5 Joint pathway analysis

The joint transcriptomic-metabolomic pathway analysis reported in the main Results was not refitted, since it derives from the MetaboAnalystR workflow described in Methods. Instead, the FDR column of the deposited joint-pathway output (file OBERON_JointPathwayAnalysis_DEHP.xlsx) was re-examined to determine how many of the enriched pathways retain FDR < 0.05. These counts are reported in Section 5.

## 3. Transcript-level results

### 3.1 Attrition under successively stricter criteria

Table S3.1 reports, for each contrast, the number of transcripts retained by the magnitude threshold alone (|log₂FC| ≥ 0.58 for the human cell models, ≥ 1.3 for zebrafish), by magnitude together with a nominal p < 0.05, and by magnitude together with BH-FDR < 0.05. The final column gives the total number of transcripts reaching FDR significance in that contrast irrespective of magnitude.

Table S3.1. Attrition of transcripts under successively stricter selection criteria. Three biological replicates per condition in the human cell systems and four in zebrafish.

| **Contrast** | **Transcripts tested** | **\|log₂FC\| threshold** | **Pass magnitude** | **Pass magnitude + p < 0.05** | **Pass magnitude + FDR < 0.05** | **Any transcript at FDR < 0.05** |
| --- | --- | --- | --- | --- | --- | --- |
| HepaRG 3D 100 pM | 21,718 | 0.58 | 1,266 | 572 | 3 | 3 |
| HepaRG 3D 100 nM | 21,718 | 0.58 | 1,153 | 519 | 3 | 3 |
| HepaRG 3D 10 µM | 21,718 | 0.58 | 3,497 | 66 | 0 | 0 |
| EndoC-βH1 1 nM | 15,532 | 0.58 | 254 | 125 | 0 | 0 |
| EndoC-βH1 10 nM | 15,532 | 0.58 | 125 | 54 | 0 | 0 |
| EndoC-βH1 100 nM | 15,532 | 0.58 | 182 | 47 | 0 | 0 |
| Zebrafish 25 nM | 14,685 | 1.3 | 245 | 220 | 0 | 0 |
| Zebrafish 10 µM | 14,685 | 1.3 | 254 | 228 | 0 | 0 |

Across the eight contrasts, between 125 and 3,497 transcripts pass the magnitude threshold and between 47 and 572 additionally pass a nominal p < 0.05, but only three transcripts in the entire study survive BH-FDR < 0.05. These three are the same three genes in two contrasts — CYP1A1, CYP1A2 and CYP1B1 in HepaRG spheroids at 100 pM and at 100 nM — and they are xenobiotic-metabolising enzymes responding to treatment rather than members of any key-event pathway. No transcript reaches FDR significance in the HepaRG 10 µM contrast, in any EndoC-βH1 contrast, or in either zebrafish contrast.

Figure S3.1 displays this attrition. The scale is symmetrical-logarithmic so that zero counts remain visible.

Figure S3.1. Sensitivity of feature selection to false-discovery-rate control. Transcripts retained in each DEHP contrast under three successively stricter criteria: the magnitude threshold alone, magnitude plus nominal p < 0.05, and magnitude plus BH-FDR < 0.05. Numerals above the third bar give the count surviving FDR. n = 3 biological replicates per condition in the human cell systems and n = 4 in zebrafish.

### 3.2 The genes named in the Results

The transcripts discussed individually in the main Results were examined specifically, since these features are cited individually in the main Results. Table S3.2 gives, for each such gene, the contrast in which it achieves its smallest adjusted p-value and the value attained.

Table S3.2. Best BH-adjusted p-value attained by each gene named in the main Results, across all eight contrasts. None reaches FDR significance.

| **Gene** | **Best contrast** | **log₂FC** | **Nominal p** | **BH-FDR** |
| --- | --- | --- | --- | --- |
| ATP5MC3 | HepaRG 3D 100 nM | -0.17 | 0.187 | 0.67 |
| CIDEC | HepaRG 3D 100 nM | +0.46 | 0.0367 | 0.67 |
| CS | HepaRG 3D 100 nM | -0.16 | 0.174 | 0.67 |
| G6PC1 | HepaRG 3D 100 nM | +0.62 | 0.00499 | 0.67 |
| PPARG | HepaRG 3D 100 nM | +0.36 | 0.127 | 0.67 |
| UCP3 | HepaRG 3D 100 nM | +2.10 | 0.024 | 0.67 |
| FH | EndoC-βH1 1 nM | -0.38 | 0.0354 | 0.68 |
| IRS1 | EndoC-βH1 1 nM | -0.38 | 0.00592 | 0.68 |
| PLIN2 | HepaRG 3D 100 pM | +0.27 | 0.126 | 0.68 |
| SDHC | HepaRG 3D 100 pM | +0.30 | 0.081 | 0.68 |
| PCK1 | HepaRG 3D 100 pM | +0.26 | 0.294 | 0.69 |
| CPT1A | HepaRG 3D 100 pM | +0.18 | 0.298 | 0.70 |
| PLIN1 | HepaRG 3D 100 pM | +0.20 | 0.44 | 0.76 |
| CIDEB | HepaRG 3D 10 µM | -0.71 | 0.127 | 0.79 |
| PHGDH | HepaRG 3D 10 µM | -0.38 | 0.332 | 0.79 |
| PPARGC1A | HepaRG 3D 10 µM | -0.33 | 0.262 | 0.79 |

Not one of these genes reaches gene-level FDR significance in any contrast. The smallest adjusted p-value attained by any of them is 0.67. Accordingly, individual transcripts are reported to document the direction and magnitude of response and are not presented as independently significant findings.

Figure S3.2. Best BH-adjusted p-value achieved by each gene named in the main Results, taken across all eight DEHP contrasts. The dashed line marks FDR = 0.05; every gene lies far to its right.

## 4. Gene-set level results

### 4.1 Competitive gene-set testing

Set-level testing gives a materially different picture from the transcript level. Of the 72 model-by-dose-by-pathway tests, 24 are significant at BH-FDR < 0.05 with correction applied within each contrast, and 23 remain significant when correction is applied globally across the entire family of 72 tests. Table S3.3 lists every significant test.

Table S3.3. Competitive gene-set tests (CAMERA) significant at BH-FDR < 0.05 within contrast. Direction is relative to matched vehicle control; z is the directional pathway score defined in Section 2.4.

| **Contrast** | **Pathway** | **Genes** | **Direction** | **z** | **p** | **BH-FDR (within)** | **BH-FDR (global)** |
| --- | --- | --- | --- | --- | --- | --- | --- |
| EndoC-βH1 100 nM | Citrate cycle (TCA cycle) | 27 | Down | -3.98 | 5.9e-4 | 0.00531 | 0.00708 |
| EndoC-βH1 100 nM | Glycolysis / gluconeogenesis | 40 | Down | -4.02 | 0.00288 | 0.013 | 0.0173 |
| EndoC-βH1 10 nM | Citrate cycle (TCA cycle) | 27 | Down | -3.67 | 0.00259 | 0.0174 | 0.0173 |
| EndoC-βH1 10 nM | Fatty acid metabolism | 32 | Down | -3.37 | 0.00388 | 0.0174 | 0.0199 |
| EndoC-βH1 10 nM | Glycolysis / gluconeogenesis | 40 | Down | -3.45 | 0.00752 | 0.0174 | 0.0279 |
| EndoC-βH1 10 nM | Oxidative phosphorylation | 97 | Down | -4.11 | 0.00775 | 0.0174 | 0.0279 |
| EndoC-βH1 10 nM | PPAR signalling pathway | 44 | Down | -3.52 | 0.0258 | 0.0465 | 0.0715 |
| HepaRG 3D 100 nM | Glycine, serine, threonine metabolism | 31 | Up | +4.10 | 0.00274 | 0.0246 | 0.0173 |
| HepaRG 3D 100 pM | Glycine, serine, threonine metabolism | 31 | Up | +5.28 | 2.2e-4 | 0.00194 | 0.0031 |
| HepaRG 3D 100 pM | Fatty acid metabolism | 42 | Up | +4.93 | 0.00312 | 0.0141 | 0.0173 |
| HepaRG 3D 10 µM | Fatty acid metabolism | 42 | Down | -6.50 | 8.1e-9 | 7.3e-8 | 2.5e-7 |
| HepaRG 3D 10 µM | PPAR signalling pathway | 62 | Down | -5.60 | 1.4e-6 | 6.2e-6 | 2.5e-5 |
| HepaRG 3D 10 µM | Glycine, serine, threonine metabolism | 31 | Down | -3.66 | 0.00105 | 0.00314 | 0.00942 |
| HepaRG 3D 10 µM | Citrate cycle (TCA cycle) | 29 | Down | -2.64 | 0.00439 | 0.00989 | 0.0211 |
| HepaRG 3D 10 µM | Glycolysis / gluconeogenesis | 56 | Down | -2.78 | 0.00705 | 0.0127 | 0.0279 |
| Zebrafish 10 µM | Oxidative phosphorylation | 100 | Down | -10.90 | 4.8e-11 | 4.3e-10 | 3.5e-9 |
| Zebrafish 10 µM | Citrate cycle (TCA cycle) | 26 | Down | -4.42 | 0.00118 | 0.00353 | 0.00942 |
| Zebrafish 10 µM | Glycolysis / gluconeogenesis | 43 | Down | -4.65 | 0.00116 | 0.00353 | 0.00942 |
| Zebrafish 10 µM | Fatty acid metabolism | 34 | Down | -3.64 | 0.00549 | 0.0124 | 0.0247 |
| Zebrafish 10 µM | PPAR signalling pathway | 56 | Down | -3.94 | 0.0136 | 0.0245 | 0.0426 |
| Zebrafish 25 nM | Oxidative phosphorylation | 100 | Down | -9.05 | 1.1e-8 | 9.5e-8 | 2.5e-7 |
| Zebrafish 25 nM | Citrate cycle (TCA cycle) | 26 | Down | -3.92 | 0.00614 | 0.0276 | 0.026 |
| Zebrafish 25 nM | Fatty acid metabolism | 34 | Down | -3.32 | 0.0123 | 0.0277 | 0.0403 |
| Zebrafish 25 nM | Glycolysis / gluconeogenesis | 43 | Down | -3.86 | 0.0109 | 0.0277 | 0.0375 |

### 4.2 Key-event pathways

The question that matters for the network is whether each pathway carrying a key event retains significance somewhere after correction. Table S3.4 answers this pathway by pathway.

Table S3.4. Best result attained by each key-event pathway across the eight contrasts.

| **KEGG pathway** | **Key event(s)** | **Best contrast** | **z** | **Best BH-FDR** | **Significant?** | **n contrasts sig.** |
| --- | --- | --- | --- | --- | --- | --- |
| Oxidative phosphorylation | KE4, KE6, KE7 | Zebrafish 10 µM | -10.90 | 4.3e-10 | yes | 3 |
| Citrate cycle (TCA cycle) | KE3 | Zebrafish 10 µM | -4.42 | 0.00353 | yes | 5 |
| Glycolysis / gluconeogenesis | KE14 | Zebrafish 10 µM | -4.65 | 0.00353 | yes | 5 |
| Fatty acid metabolism | KE1, KE2 | HepaRG 3D 10 µM | -6.50 | 7.3e-8 | yes | 5 |
| PPAR signalling pathway | MIE | HepaRG 3D 10 µM | -5.60 | 6.2e-6 | yes | 3 |
| Glycine, serine, threonine metabolism | KE8 | HepaRG 3D 100 pM | +5.28 | 0.00194 | yes | 3 |
| Sphingolipid metabolism | KE11 | HepaRG 3D 100 pM | +2.80 | 0.146 | no | 0 |
| Insulin signalling pathway | KE10, KE12 | Zebrafish 10 µM | -4.20 | 0.0529 | no | 0 |
| Type II diabetes mellitus | KE12, KE14 | Zebrafish 10 µM | -1.97 | 0.373 | no | 0 |

Six of the nine key-event pathways reach FDR significance in at least one system: oxidative phosphorylation, the citrate (TCA) cycle, glycolysis/gluconeogenesis, fatty acid metabolism, PPAR signalling, and glycine/serine/threonine metabolism. Together these carry the MIE and key events KE1 to KE8 and KE14 — that is, the mitochondrial and lipid-handling core of the network — and each is significant in three to five independent contrasts rather than in a single one.

Three pathways do not reach the corrected threshold, and we state this plainly rather than presenting the network as uniformly supported. Sphingolipid metabolism shows consistent low-dose hepatic induction (z = +2.80 at 100 pM, +2.78 at 100 nM; best BH-FDR = 0.15) but does not survive correction in this gene-set formulation; the evidence for S1P disruption (KE11) is primarily metabolomic rather than transcriptomic and rests on the directly measured S1P and ceramide species reported in the main text, not on this test. The insulin signalling pathway falls just short of the threshold, with a coherent and consistent zebrafish suppression that reaches BH-FDR = 0.053 at both concentrations and a hepatic low-dose induction at 0.063; this is suggestive but is not a significant result and is not treated as one. Type II diabetes mellitus is a composite disease annotation rather than a mechanistic pathway and is not diagnostic of a single key event. Accordingly, KE10, KE11 and KE12 are supported in the network by metabolomic and functional evidence, including the insulin-secretion endpoints measured within OBERON, rather than by transcriptomic set-level significance. The main text and Supplementary Material 1 identify these evidence sources explicitly.

### 4.3 Conserved and divergent responses across systems

Figure S3.3 shows the full directional pathway score matrix underlying the cross-system comparison of the main text. Three patterns are evident.

First, suppression of oxidative-energy metabolism is the only module conserved across all three systems. Oxidative phosphorylation, the TCA cycle and glycolysis/gluconeogenesis are suppressed in every EndoC-βH1 and zebrafish contrast and in HepaRG at 10 µM, with the strongest effect in zebrafish (z = −9.05 at 25 nM and −10.90 at 10 µM, BH-FDR = 9.5 × 10⁻⁸ and 4.3 × 10⁻¹⁰). This is the mitochondrial core of the network, and it is the part supported concordantly at all three levels of biological organisation.

Second, PPAR signalling and fatty-acid handling are liver-dominant and non-monotonic with concentration. In HepaRG spheroids both are induced at 100 pM (z = +3.53 and +4.93) and strongly suppressed at 10 µM (z = −5.60 and −6.50, BH-FDR = 6.2 × 10⁻⁶ and 7.3 × 10⁻⁸). The reversal of sign between the lowest and highest concentration is the statistically supported non-monotonicity discussed in the main text. Neither pathway shows coherent induction in zebrafish.

Third, the serine arm is detected principally in the hepatic system: glycine, serine and threonine metabolism is significantly induced in HepaRG at 100 pM and 100 nM (z = +5.28 and +4.10) and is not significant in either other system. The insulin arm shows the same hepatic low-dose induction and a zebrafish suppression, neither reaching the corrected threshold as noted above.

Figure S3.3. Conserved versus divergent pathway responses across the three experimental systems. Cell values are directional pathway scores z (Section 2.4); blue denotes coordinated suppression and red coordinated induction, with the colour scale centred at z = 0. Asterisks mark contrasts significant at BH-FDR < 0.05 within contrast in competitive gene-set testing. n = 3 biological replicates per condition in the human cell systems and n = 4 in zebrafish.

## 5. Joint transcriptomic-metabolomic pathway analysis

The joint pathway analysis reported in the main Results comprises 408 enriched pathway results across the three models and the univariate and multivariate workflows. Of these, 48 retain FDR < 0.05. Table S3.5 summarises the distribution and Table S3.6 lists the pathways of direct relevance to the network.

Table S3.5. Joint pathway analysis results retaining FDR < 0.05, by model and workflow.

| **Model** | **Multi Omics Method** | **Total tested** | **Pathways at FDR < 0.05** |
| --- | --- | --- | --- |
| Liver (3D HepaRG cells) | GeneSpring - Multivariate | 6 | 0 |
| Liver (3D HepaRG cells) | GeneSpring - Univariate | 148 | 14 |
| Liver (3D HepaRG cells) | Limma - Multivariate | 6 | 0 |
| Liver (3D HepaRG cells) | Limma - Univariate | 111 | 16 |
| Pancreas (EndoC-βH1 cells) | GeneSpring - Multivariate | 7 | 0 |
| Pancreas (EndoC-βH1 cells) | GeneSpring - Univariate | 46 | 0 |
| Pancreas (EndoC-βH1 cells) | Limma - Multivariate | 11 | 0 |
| Pancreas (EndoC-βH1 cells) | Limma - Univariate | 35 | 5 |
| Zebrafish 5dpf | GeneSpring - Multivariate | 7 | 2 |
| Zebrafish 5dpf | GeneSpring - Univariate | 2 | 0 |
| Zebrafish 5dpf | Limma - Multivariate | 6 | 1 |
| Zebrafish 5dpf | Limma - Univariate | 23 | 10 |

Table S3.6. Key-event-relevant pathways from the joint transcriptomic-metabolomic analysis that retain FDR < 0.05.

| **Model** | **Treatment** | **Workflow** | **Pathway** | **Hits** | **Raw p** | **FDR** |
| --- | --- | --- | --- | --- | --- | --- |
| Zebrafish 5dpf | All | Limma - Univariate | Citrate cycle (TCA cycle) | 11 | 0.00115 | 0.0208 |
| Zebrafish 5dpf | All | Limma - Univariate | Oxidative phosphorylation | 55 | 3.8e-25 | 3.1e-23 |

The zebrafish oxidative phosphorylation result (FDR = 3.1 × 10⁻²³) and the zebrafish TCA cycle result (FDR = 0.021) are the two joint-analysis findings quoted in the main text, and both are confirmed here against the deposited output. Consistent with the gene-set analysis of Section 4, the surviving joint-analysis pathways are concentrated on the mitochondrial and lipid-handling core of the network.

## 6. Interpretation and consequences for the network

The reanalysis supports three main conclusions.

First, statistical support is limited at the level of the individual feature. At three to four biological replicates in a testing problem of 15,000 to 22,000 dimensions, gene-level FDR control is essentially unattainable for effects of the size observed here. Only three transcripts in the whole study survive correction, and none of them is a gene cited in the mechanistic narrative. Individual log₂FC values reported in the main text and in Supplementary Material 1 are therefore descriptive annotations of direction and magnitude, not independent evidence, and should be interpreted accordingly.

Second, the concern does not extend to the pathway level. Competitive gene-set testing aggregates evidence across the tens to hundreds of genes constituting a pathway and is adequately powered at this replicate number. Six of the nine key-event pathways — those carrying the MIE and the mitochondrial and lipid-handling key events that form the spine of the network — are significant after FDR correction, most of them in three to five independent contrasts. The evidential architecture therefore rests on these set-level statistics.

Third, the analysis shows identifies the less strongly supported components of the network. The sphingolipid and insulin arms do not reach set-level transcriptomic significance, and their key events are supported instead by metabolomic measurement and by functional endpoints generated within OBERON. Supplementary Material 1 now carries an explicit evidence-type declaration for every key event, distinguishing endpoints measured in this study, endpoints measured within OBERON, endpoints inferred from molecular abundance, endpoints predicted by the systems biology model, and events supported by literature alone.

Taken together, these results support describing the framework as hypothesis-generating and evidence-integrating rather than quantitatively validated and support anchoring the key events are anchored at pathway rather than single-feature level. They also identify the single most valuable addition to the experimental design: replication at n ≥ 6 per condition, which would bring gene-level FDR control within reach for effects of the magnitude observed here.

## 7. Limitations of this reanalysis

The reanalysis inherits the normalisation and preprocessing of the original workflow, since it starts from the deposited normalised matrices rather than from raw intensity files; it is a sensitivity analysis of feature selection and inference, not an independent reprocessing from raw data.

Gene sets were taken from the MSigDB distribution of KEGG rather than from live KEGG, for the reason stated in Section 2.3, so individual z-scores and adjusted p-values differ in detail from those in the main text while the qualitative pattern is unchanged.

Metabolomic features were not refitted here. The metabolomics workflow described in Methods already applies Benjamini-Hochberg correction with an adjusted p cut-off of 0.05, so metabolite-level findings were subject to FDR control in the original analysis; the joint pathway results of Section 5 incorporate both layers.

Finally, the directional pathway score of Section 2.4 is a descriptive summary of coordinated direction and magnitude. It is not itself a test statistic, and significance is always taken from the accompanying CAMERA result rather than from the score.

## 8. Data and code availability

The per-sample normalised expression matrices used as input are deposited at doi:10.5281/zenodo.20376941 (file OBERON_DEHP_Metbolites_genes_summary.xlsx, sheets "Liver - LIMMA - Universe", "Pancreas - LIMMA - Universe" and "ZF- Pilot - LIMMA - genes"). The joint pathway analysis output is in the same record (file OBERON_JointPathwayAnalysis_DEHP.xlsx, sheet "Pathways").

The complete machine-readable outputs of this reanalysis accompany this document as comma-separated files, listed in Table S3.7:

Table S3.7. Machine-readable files accompanying this Supplementary Material.

| **File** | **Rows** | **Content** |
| --- | --- | --- |
| SM3_TableS3.1_per_gene_statistics.csv | 141,120 | Refitted per-gene statistics (logFC, AveExpr, t, p, BH-FDR) for all eight contrasts |
| SM3_TableS3.2_attrition_counts.csv | 8 | Attrition counts under each selection criterion, per contrast |
| SM3_TableS3.3_camera_geneset_results.csv | 72 | Complete CAMERA gene-set results with within-contrast and global BH correction |
| SM3_TableS3.4_pathway_score_matrix.csv | 9 × 8 | Directional pathway score matrix underlying Figure S3.3 |
| SM3_TableS3.5_named_genes.csv | 84 | Per-contrast statistics for every gene named in the main Results |
| SM3_TableS3.6_joint_pathway_FDR.csv | 48 | Joint pathway analysis results retaining FDR < 0.05 |
| SM3_reanalysis.R | — | Reproducible analysis script generating all tables and figures in this document |
| sessionInfo.txt | — | R session and package versions used |

The analysis script is reproduced in Section 9 and is also provided as a standalone file. Running it against the deposited matrices regenerates every number and figure in this document.

## 9. Analysis code

R 4.4 with limma 3.62.1 and msigdbr 26.1.0. The script expects the four per-sample matrices exported from the deposited workbook as CSV in the working directory.

```
## ---------------------------------------------------------------------------
## Supplementary Material 3 - FDR-controlled sensitivity reanalysis
## Manuscript: Mapping DEHP-Induced Metabolic Dysregulation Through Multi-Omics
##             and Systems Biology: An AOP Network Approach
##
## Input : per-sample normalised log2 expression matrices deposited at
##         doi:10.5281/zenodo.20376941 (OBERON_DEHP_Metbolites_genes_summary.xlsx)
## Output: per-gene refitted statistics, attrition counts, CAMERA gene-set
##         results and the cross-system directional pathway score matrix.
## ---------------------------------------------------------------------------

suppressMessages({library(limma); library(msigdbr)})
set.seed(1)
OUT <- "out"; dir.create(OUT, showWarnings = FALSE)

## --- 1. Load per-sample matrices -------------------------------------------
liver <- read.csv("liver_expr.csv",    check.names = FALSE)
panc  <- read.csv("panc_expr.csv",     check.names = FALSE)
zf    <- read.csv("zf_pilot_expr.csv", check.names = FALSE)

## --- 2. Contrast definitions (8 DEHP contrasts) ----------------------------
## Each entry: matrix, control columns, treated columns, magnitude threshold.
contrasts <- list(
  list(id="HepaRG_100pM",   model="HepaRG 3D",   dose="100 pM",  org="Hs", d=liver,
       ctrl=c("Cntrl_4_1","Cntrl_4_2","Cntrl_4_3"),
       trt =c("DEHP_100pM_1","DEHP_100pM_2","DEHP_100pM_3"), thr=0.58),
  list(id="HepaRG_100nM",   model="HepaRG 3D",   dose="100 nM",  org="Hs", d=liver,
       ctrl=c("Cntrl_4_1","Cntrl_4_2","Cntrl_4_3"),
       trt =c("DEHP_100nM_1","DEHP_100nM_2","DEHP_100nM_3"), thr=0.58),
  list(id="HepaRG_10uM",    model="HepaRG 3D",   dose="10 uM",   org="Hs", d=liver,
       ctrl=c("Cntrl_4_1","Cntrl_4_2","Cntrl_4_3"),
       trt =c("DEHP_10uM_1","DEHP_10uM_2","DEHP_10uM_3"), thr=0.58),
  list(id="EndoC_1nM",      model="EndoC-bH1",   dose="1 nM",    org="Hs", d=panc,
       ctrl=c("Cntrl_1","Cntrl_2","Cntrl_3"),
       trt =c("DEHP_1nM_1","DEHP_1nM_2","DEHP_1nM_3"), thr=0.58),
  list(id="EndoC_10nM",     model="EndoC-bH1",   dose="10 nM",   org="Hs", d=panc,
       ctrl=c("Cntrl_1","Cntrl_2","Cntrl_3"),
       trt =c("DEHP_10nM_1","DEHP_10nM_2","DEHP_10nM_3"), thr=0.58),
  list(id="EndoC_100nM",    model="EndoC-bH1",   dose="100 nM",  org="Hs", d=panc,
       ctrl=c("Cntrl_1","Cntrl_2","Cntrl_3"),
       trt =c("DEHP_100nM_1","DEHP_100nM_2","DEHP_100nM_3"), thr=0.58),
  list(id="Zebrafish_25nM", model="Zebrafish",   dose="25 nM",   org="Dr", d=zf,
       ctrl=c("DMSO_1","DMSO_2","DMSO_3","DMSO_4"),
       trt =c("DEHP25_1","DEHP25_2","DEHP25_3","DEHP25_4"), thr=1.3),
  list(id="Zebrafish_10uM", model="Zebrafish",   dose="10 uM",   org="Dr", d=zf,
       ctrl=c("DMSO_1","DMSO_2","DMSO_3","DMSO_4"),
       trt =c("DEHP10_1","DEHP10_2","DEHP10_3","DEHP10_4"), thr=1.3)
)

## --- 3. Key-event pathway gene sets ----------------------------------------
## KEGG pathway definitions retrieved offline from MSigDB (C2:CP:KEGG_LEGACY).
KE_SETS <- c(
  "Oxidative phosphorylation"              = "KEGG_OXIDATIVE_PHOSPHORYLATION",
  "Citrate cycle (TCA cycle)"              = "KEGG_CITRATE_CYCLE_TCA_CYCLE",
  "Fatty acid metabolism"                  = "KEGG_FATTY_ACID_METABOLISM",
  "PPAR signalling pathway"                = "KEGG_PPAR_SIGNALING_PATHWAY",
  "Glycolysis / gluconeogenesis"           = "KEGG_GLYCOLYSIS_GLUCONEOGENESIS",
  "Glycine, serine, threonine metabolism"  = "KEGG_GLYCINE_SERINE_AND_THREONINE_METABOLISM",
  "Sphingolipid metabolism"                = "KEGG_SPHINGOLIPID_METABOLISM",
  "Insulin signalling pathway"             = "KEGG_INSULIN_SIGNALING_PATHWAY",
  "Type II diabetes mellitus"              = "KEGG_TYPE_II_DIABETES_MELLITUS"
)

get_sets <- function(org) {
  sp <- if (org == "Hs") "Homo sapiens" else "Danio rerio"
  m  <- msigdbr(species = sp, collection = "C2", subcollection = "CP:KEGG_LEGACY")
  m  <- m[m$gs_name %in% KE_SETS, ]
  s  <- split(as.character(m$ncbi_gene), m$gs_name)
  s  <- lapply(s, function(v) unique(v[!is.na(v) & v != ""]))
  names(s) <- names(KE_SETS)[match(names(s), KE_SETS)]
  s[!vapply(s, function(v) length(v) == 0, logical(1))]
}
sets <- list(Hs = get_sets("Hs"), Dr = get_sets("Dr"))

## --- 4. Per-contrast refit --------------------------------------------------
per_gene <- list(); attrition <- list(); camera_all <- list()

for (cs in contrasts) {
  d <- cs$d
  E <- as.matrix(d[, c(cs$ctrl, cs$trt)])
  rownames(E) <- as.character(d$ENTREZID)
  keep <- rowSums(is.na(E)) == 0 & apply(E, 1, function(x) var(x) > 0)
  E <- E[keep, , drop = FALSE]

  grp <- factor(c(rep("ctrl", length(cs$ctrl)), rep("trt", length(cs$trt))),
                levels = c("ctrl", "trt"))
  ## Paired design: control and treated arms were processed as matched
  ## experimental replicates, so replicate is included as a blocking factor
  ## (SM2 identifies experimental replicate as the dominant variance component).
  nrep  <- length(cs$ctrl)
  block <- factor(rep(seq_len(nrep), 2))
  design <- model.matrix(~ block + grp)
  coefname <- "grptrt"

  ## limma with robust empirical-Bayes moderation and intensity trend
  fit <- eBayes(lmFit(E, design), robust = TRUE, trend = TRUE)
  tt  <- topTable(fit, coef = coefname, number = Inf, sort.by = "none")
  tt$ENTREZID <- rownames(E)
  tt$contrast <- cs$id; tt$model <- cs$model; tt$dose <- cs$dose

  pg <- data.frame(contrast = cs$id, model = cs$model, dose = cs$dose,
                   ENTREZID = tt$ENTREZID, logFC = tt$logFC,
                   AveExpr = tt$AveExpr, t = tt$t,
                   P.Value = tt$P.Value, BH.FDR = tt$adj.P.Val,
                   stringsAsFactors = FALSE)
  per_gene[[cs$id]] <- pg

  ## attrition under three successively stricter criteria
  mag  <- abs(pg$logFC) >= cs$thr
  attrition[[cs$id]] <- data.frame(
    contrast = cs$id, model = cs$model, dose = cs$dose,
    n_tested = nrow(pg), magnitude_threshold = cs$thr,
    pass_magnitude          = sum(mag),
    pass_magnitude_nominalP = sum(mag & pg$P.Value < 0.05),
    pass_magnitude_BHFDR    = sum(mag & pg$BH.FDR  < 0.05),
    total_BHFDR_sig         = sum(pg$BH.FDR < 0.05),
    stringsAsFactors = FALSE)

  ## competitive gene-set testing (CAMERA, fixed inter-gene correlation 0.01)
  S  <- sets[[cs$org]]
  idx <- ids2indices(S, rownames(E), remove.empty = TRUE)
  if (length(idx) > 0) {
    cam <- camera(E, idx, design, contrast = coefname,
                  inter.gene.cor = 0.01, sort = FALSE)
    ## directional pathway score z = standardised mean moderated t of set members
    tstat <- tt$t; names(tstat) <- rownames(E)
    z <- vapply(idx, function(i) {
      ti <- tstat[i]; sqrt(length(ti)) * mean(ti) / sd(tstat)
    }, numeric(1))
    camera_all[[cs$id]] <- data.frame(
      contrast = cs$id, model = cs$model, dose = cs$dose,
      pathway = rownames(cam), NGenes = cam$NGenes,
      Direction = cam$Direction, z_score = as.numeric(z[rownames(cam)]),
      PValue = cam$PValue, BH.FDR = cam$FDR, stringsAsFactors = FALSE)
  }
}

per_gene   <- do.call(rbind, per_gene)
attrition  <- do.call(rbind, attrition)
camera_all <- do.call(rbind, camera_all)

## BH correction across the whole family of model-by-dose-by-pathway tests
camera_all$BH.FDR.global <- p.adjust(camera_all$PValue, method = "BH")

## --- 5. Cross-system directional score matrix (basis of Figure 9) ----------
zmat <- reshape(camera_all[, c("pathway", "contrast", "z_score")],
                idvar = "pathway", timevar = "contrast", direction = "wide")
names(zmat) <- sub("^z_score\\.", "", names(zmat))

## --- 6. Named-gene lookup (genes cited in the Results) ----------------------
NAMED <- c(PPARG=5468, PPARGC1A=10891, CIDEB=27141, CIDEC=63924, PLIN1=5346,
           PLIN2=123, CS=1431, FH=2271, SDHC=6391, IRS1=3667, UCP3=7352,
           ATP5MC3=518, NDUFA6=4700, PHGDH=26227, CPT1A=1374, PCK1=5105, G6PC1=2538)
named_tab <- per_gene[per_gene$ENTREZID %in% as.character(NAMED), ]
named_tab$symbol <- names(NAMED)[match(named_tab$ENTREZID, as.character(NAMED))]
named_tab <- named_tab[order(named_tab$symbol, named_tab$contrast), ]

## --- 7. Write outputs ------------------------------------------------------
write.csv(per_gene,   file.path(OUT, "SM3_TableS3.1_per_gene_statistics.csv"), row.names = FALSE)
write.csv(attrition,  file.path(OUT, "SM3_TableS3.2_attrition_counts.csv"),    row.names = FALSE)
write.csv(camera_all, file.path(OUT, "SM3_TableS3.3_camera_geneset_results.csv"), row.names = FALSE)
write.csv(zmat,       file.path(OUT, "SM3_TableS3.4_pathway_score_matrix.csv"), row.names = FALSE)
write.csv(named_tab,  file.path(OUT, "SM3_TableS3.5_named_genes.csv"),         row.names = FALSE)

sink(file.path(OUT, "sessionInfo.txt")); print(sessionInfo()); sink()

cat("=== ATTRITION ===\n"); print(attrition, row.names = FALSE)
cat("\n=== CAMERA (BH within contrast < 0.05) ===\n")
print(camera_all[camera_all$BH.FDR < 0.05,
                 c("contrast","pathway","NGenes","Direction","z_score","PValue","BH.FDR")],
      row.names = FALSE)
cat("\nCAMERA tests total:", nrow(camera_all),
    " significant (within-contrast BH):", sum(camera_all$BH.FDR < 0.05),
    " significant (global BH):", sum(camera_all$BH.FDR.global < 0.05), "\n")
cat("\n=== NAMED GENES: best BH-FDR ===\n")
bg <- aggregate(BH.FDR ~ symbol, data = named_tab, FUN = min)
print(bg[order(bg$BH.FDR), ], row.names = FALSE)

```

## 10. Session information

```
R version 4.4.3 (2025-02-28)
Platform: x86_64-conda-linux-gnu

Matrix products: default
BLAS/LAPACK: /home/denis/.claude-science/conda/envs/bioc-limma/lib/libopenblasp-r0.3.33.so;  LAPACK version 3.12.0

locale:
 [1] LC_CTYPE=C.UTF-8       LC_NUMERIC=C           LC_TIME=C.UTF-8
 [4] LC_COLLATE=C.UTF-8     LC_MONETARY=C.UTF-8    LC_MESSAGES=C.UTF-8
 [7] LC_PAPER=C.UTF-8       LC_NAME=C              LC_ADDRESS=C
[10] LC_TELEPHONE=C         LC_MEASUREMENT=C.UTF-8 LC_IDENTIFICATION=C

time zone: NA
tzcode source: system (glibc)

attached base packages:
[1] stats     graphics  grDevices utils     datasets  methods   base

other attached packages:
[1] msigdbr_26.1.0 limma_3.62.1

loaded via a namespace (and not attached):
 [1] babelgene_22.9   assertthat_0.2.1 R6_2.6.1         tidyselect_1.2.1
 [5] magrittr_2.0.5   splines_4.4.3    glue_1.8.1       tibble_3.3.1
 [9] pkgconfig_2.0.3  generics_0.1.4   dplyr_1.2.1      lifecycle_1.0.5
[13] cli_3.6.6        vctrs_0.7.3      withr_3.0.3      statmod_1.5.2
[17] compiler_4.4.3   tools_4.4.3      curl_7.1.0       pillar_1.11.1
[21] rlang_1.3.0

```
